# Uncovering multi-faceted taxonomic and functional diversity of soil bacteriomes in tropical Southeast Asian countries

**DOI:** 10.1038/s41598-020-79786-x

**Published:** 2021-01-12

**Authors:** Somsak Likhitrattanapisal, Paopit Siriarchawatana, Mintra Seesang, Suwanee Chunhametha, Worawongsin Boonsin, Chitwadee Phithakrotchanakoon, Supattra Kitikhun, Lily Eurwilaichitr, Supawadee Ingsriswang

**Affiliations:** Thailand Bioresource Research Center (TBRC), National Center for Genetic Engineering and Biotechnology (BIOTEC), National Science and Technology Development Agency (NSTDA), Pathumthani, Thailand

**Keywords:** Computational biology and bioinformatics, Ecology, Microbiology

## Abstract

Environmental microbiomes encompass massive biodiversity and genetic information with a wide-ranging potential for industrial and agricultural applications. Knowledge of the relationship between microbiomes and environmental factors is crucial for translating that information into practical uses. In this study, the integrated data of Southeast Asian soil bacteriomes were used as models to assess the variation in taxonomic and functional diversity of bacterial communities. Our results demonstrated that there were differences in soil bacteriomes across different geographic locality with different soil characteristics: soil class and pH level. Such differences were observed in taxonomic diversity, interspecific association patterns, and functional diversity of soil bacteriomes. The bacterial-mediated biogeochemical cycles of nitrogen, sulfur, carbon, and phosphorus illustrated the functional relationship of soil bacteriome and soil characteristics, as well as an influence from bacterial interspecific interaction. The insights from this study reveal the importance of microbiome data integration for future microbiome research.

## Introduction

Recent advancements in high-throughput sequencing and metagenomic technologies attract wide academic interests in microbiome studies, leading to accumulation of public microbiome data. These troves of accumulated microbiome data confer the opportunities to study microbiomes at the landscape, regional, and global scales^1–3^. Although Southeast Asia is known as one of the global biodiversity hotpots, the information about microbial diversity in the region is relatively scarce and underutilised^4^. This study focused on the ecological and biological implication of the environmental microbiome data available in AmiBase (https://www.amibase.org), which is the Association of Southeast Asian Nations (ASEAN)’s comprehensive database with integrated microbial biodiversity information from metagenome and microbiome research projects in the ASEAN countries. The AmiBase project was initiated by Thailand Bioresource Research Center (TBRC) and supported by ASEAN Science, Technology and Innovation Fund (ASTIF), under the collaboration of ASEAN network on Microbial Utilisation (AnMicro) and ASEAN Centre for Biodiversity (ACB).

In this study, soil microbiomes from Thailand, the Philippines, Malaysia, and Indonesia were selected as models since these four tropical Southeast Asian countries are located in the area with unique geographic locality and climates. In terms of geographic locality, Thailand and Malaysia are situated in mainland Southeast Asia, while Indonesia and the Philippines are archipelagos lying along the Ring of Fire. In terms of climate, even though these four countries are in the tropical region, there are discernible variations. Thailand have relatively drier, tropical savanna climate. The major land areas of Malaysia and Indonesia straddle near or on the equator, so their dominant climate types are tropical rainforest and tropical oceanic climates. Philippines have tropical climates, highly dominated by monsoon. Soil habitats encompass diverse microbial communities with huge potential for utilisation in industrial and agricultural applications as well as conservation effort^5^. The tropical ecosystem in Southeast Asia is delicately intricate and has been influenced by climatic changes and geographic factors^6,7^. Comparative studies of the relationship between soil microbiomes and environmental factors are of critical importance for translating information into practical uses. However, microbial communities and population dynamics are immensely complex and can involve various factors across environmental landscapes^8–10^. Investigating every individual microbe in its natural habitats is practically unattainable because most microbes may exhibit different phenotypic characteristics in different environments^11^. Therefore, the integrated analysis was employed on the integrated microbiome data to obtain an information snapshot which enabled us to unveil the interspecific interaction patterns within the soil bacteriomes.

The purpose of this study was not only to explore the diversity and interspecific associations in the soil bacteriomes across different tropical Southeast Asian countries, but also to investigate relationship of bacterial functional diversity with soil characteristics. In particular, the functional diversity of soil bacteriome is known to play a significant role in biogeochemical cycles of four major elements, including carbon (C), nitrogen (N), phosphorus (P), and sulfur (S)^12,13^. N and S biogeochemical cycles mainly consist of redox reactions catalyzed by microbial activities^14,15^. C is utilised by microbial communities in various metabolic processes^16,17^. P is one of the major growth-limiting factors for all biota and P availability is also intertwined with the soil C cycling via P mineralization and solubilization reactions mediated by microbial enzymes^18^. Stoichiometric regulation of these key elements is responsible for maintaining soil fertility and long-term quality of terrestrial ecosystems^13,16^. Therefore, understanding the functional diversity of soil microbiomes is vital in unveiling the mechanisms that govern the local and regional ecosystems^19,20^.

In this study, bioinformatics approaches were employed to uncover the influence of geographic locality and soil characteristics in the taxonomic and functional composition of soil bacterial communities from the selected Southeast Asian countries. The differentiation of soil characteristics (soil classes and pH levels) which observed in different geographic locality demonstrated the implications on taxonomic and functional diversity profiles of soil bacteriomes. Moreover, the interspecific associations in soil bacteriomes showed some relations to the bacterial-mediated biogeochemical cycles of N, S, C and P.

## Results

### The soil bacterial diversity

**Figure 1:**
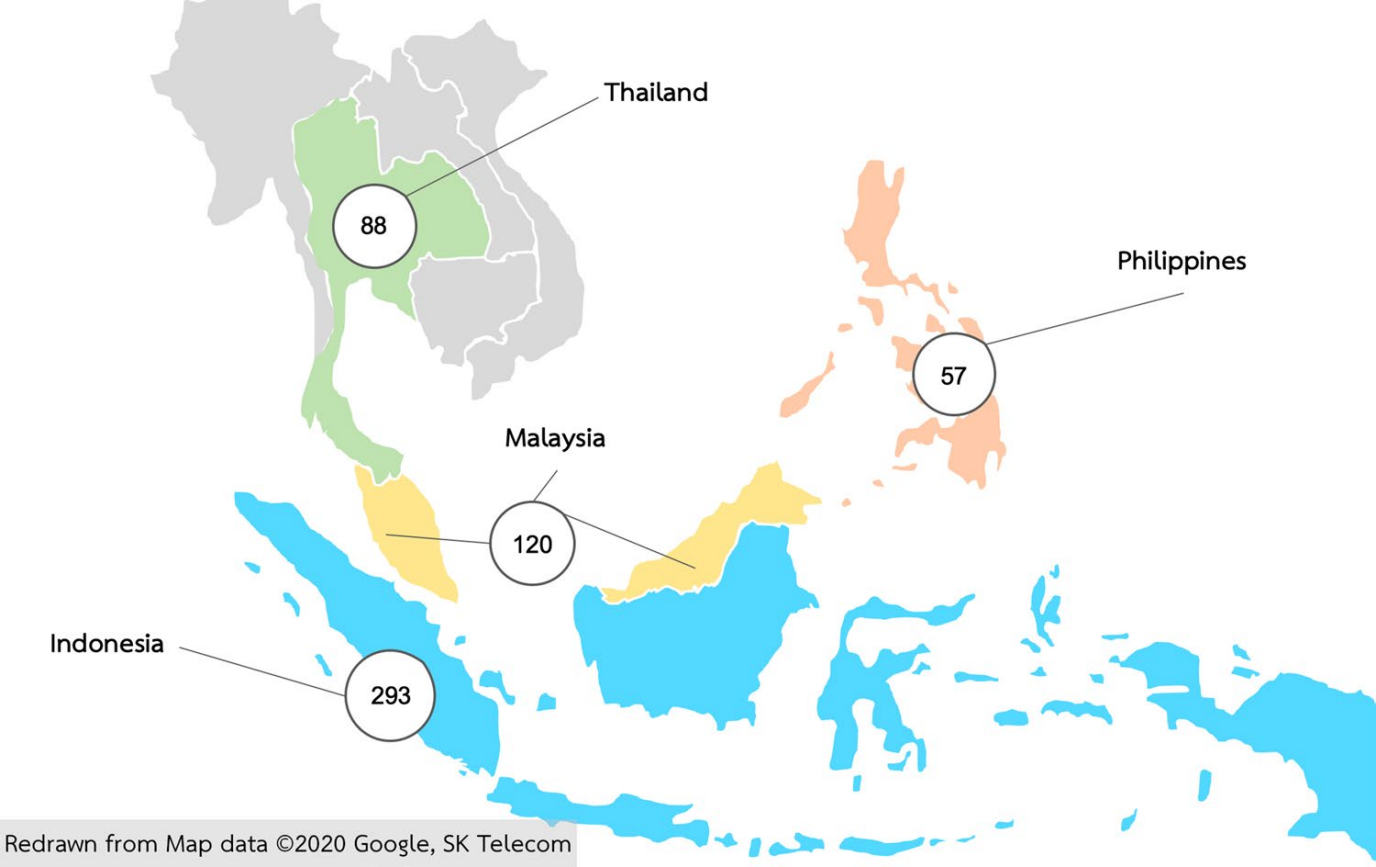
The number of soil samples from the selected Southeast Asian countries which were included in this study. The number in each circle represented the number of samples from each country. The Southeast Asia map was redrawn from “Southeast Asia” map (*Google Maps* retrieved 7 May 2020, from https://www.google.com/maps/@8.2763609,98.123781,4z).

The soil bacteriome dataset in this study included 558 soil samples collected from Thailand, the Philippines, Malaysia, and Indonesia (Fig. 1).

**Figure 2:**
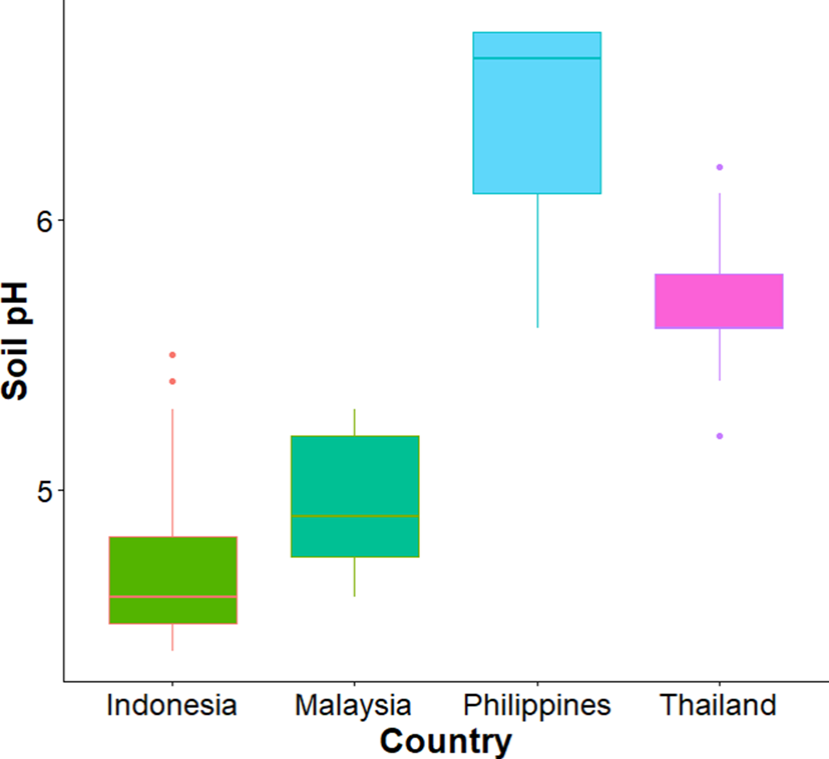
The soil pH level (at the 0-5 cm depth) of the soil samples from the four countries were significantly different.

**Figure 3:**
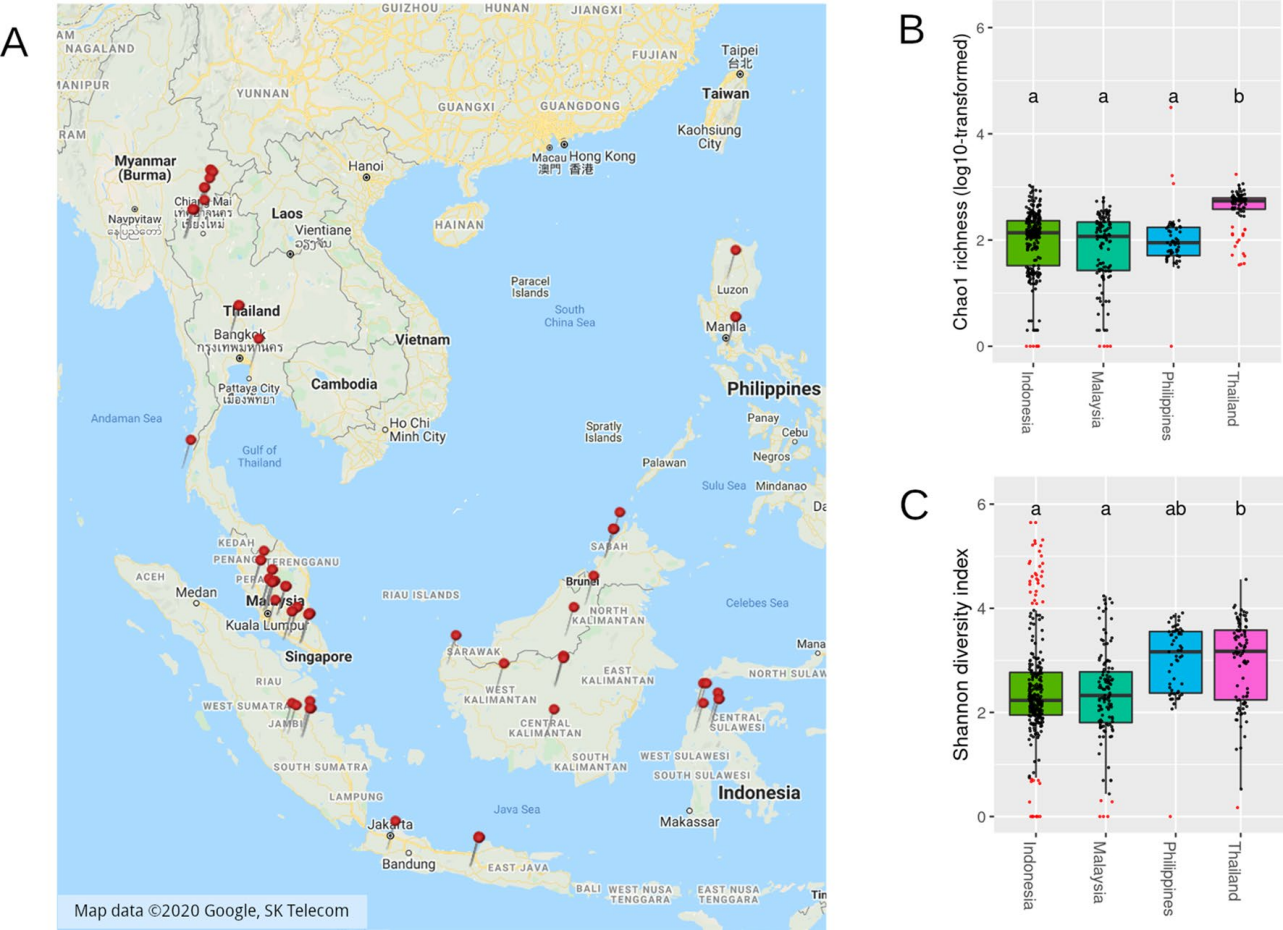
The soil bacterial diversity in relation to geographic locality. The geographic distribution of soil microbiome samples included in this study was shown (A). The map and sample coordinates were plotted using *Google Maps* data and API retrieved 7 May 2020, from https://www.google.com/maps/@8.2763609,98.123781,4z. The alpha diversity of biosamples was estimated by Chao1 species richness estimator (B) and Shannon’s diversity index (C). The letters over each box plot showed the significant difference groups tested by ANOVA with Tukey HSD post-hoc comparison (*P-*value < 0.001).

Mapping to the global gridded soil information system: SoilGrids^21^, the soil samples of each selected country encompassed different soil classes (Supplementary Figure S2). The soil from Thailand samples were mostly Acrisols, which comprise clay-rich subsoil with low fertility and high aluminium content. The soil from the Philippines samples were mostly Gleysols, iron-rich wetland soil saturated with groundwater or underwater or in tidal areas. The soil from Malaysia samples were mostly Ferralsols. The soils from Indonesia samples were of mixed soil classes; nearly half (45%) of them belonged to Nitisols, well-drained soil with a moderate-to-high clay content and limited phosphorus availability. Ferralsols took up about 20% of the Indonesia soil samples while another 18% were Histosols (moist soils with thick organic layers). The soil pH levels were significantly different among the soil samples of 4 selected countries (ANOVA, *P* value < 0.01) (Fig. 2). The location of soil samples included in this study were shown in Fig. 3A.

Alpha diversity of bacterial species in the soil samples were assessed using Chao1 species richness estimator (Fig. 3B) and Shannon’s diversity index (Fig. 3C). Thailand soil bacteriomes harbored significantly higher species richness and Shannon’s diversity index than the others (*P-*value < 0.001). The Shannon’s diversity indices of Indonesia soil samples had wider range than those of other countries’ samples. In total the soil bacteriomes of the soil samples from the selected four countries contained 11,893 bacterial species, which predominantly belonged to Proteobacteria, Actinobacteria, Firmicutes, Bacteroidetes, and Cyanobacteria (Supplementary Figure S1).

### Differential taxonomic richness in the soil bacteriomes

The taxonomic richness comparison showed that, when compared the taxonomic richness based on the number of genera, there were 350 differential families among the 4 countries (Supplementary Table S1). On the other comparisons which based on the number of species, significant species richness shifts were detected in 18 differential families and 117 differential genera (Supplementary Table S1-S2). The differential taxon was a family or a genus that demonstrated a significant differential shift in richness (number of member genera or species within that family or genus) across 4 countries.

Based on genus richness (number of member genera within each family), Flavobacteriaceae was found to be one of the most diverse bacterial families (i.e., families with high numbers of member genera) in the soil bacteriomes and differentially exhibited the highest genus richness in Thailand with 24 genera (Supplementary Table S1). Moraxellaceae and Chitinophagaceae were the differential bacterial families that harbored a high genus richness specifically to Malaysia. Several families including Peptococcaceae, Paenibacillaceae, Spirochaetaceae, Desulfobulbaceae, Syntrophobacteraceae, and Alteromonadaceae, exhibited significantly higher genus richness in the Philippines soil bacteriome than in the other countries. On the other hand, Bradyrhizobiaceae exhibited differentially high richness in Indonesia.

But when comparing species richness (number of member species within each family), Micromonosporaceae was the differential family with the highest species richness. The soil bacteriome of each country contained more than 30 species of Micromonosporaceae (Philippines = 59, Indonesia = 49, and Thailand = 36), except in Malaysia’s where only 9 species of this family were found (Supplementary Table S1). Several families including Anaerolineaceae, Nostocaceae, Sporomusaceae, Desulfobulbaceae, Syntrophobacteraceae, and Syntrophobacteraceae, exhibited differentially higher species richness in the Philippines soil bacteriomes than in the other countries.

At the genus level, it was noted that differential genera were small genera (i.e., genera with small numbers of member species). The most diverse differential genus was *Legionella*, which differentially harbored higher species richness in Thailand than in the other countries (Supplementary Table S2).

### Species abundance patterns in the soil bacteriomes

**Figure 4:**
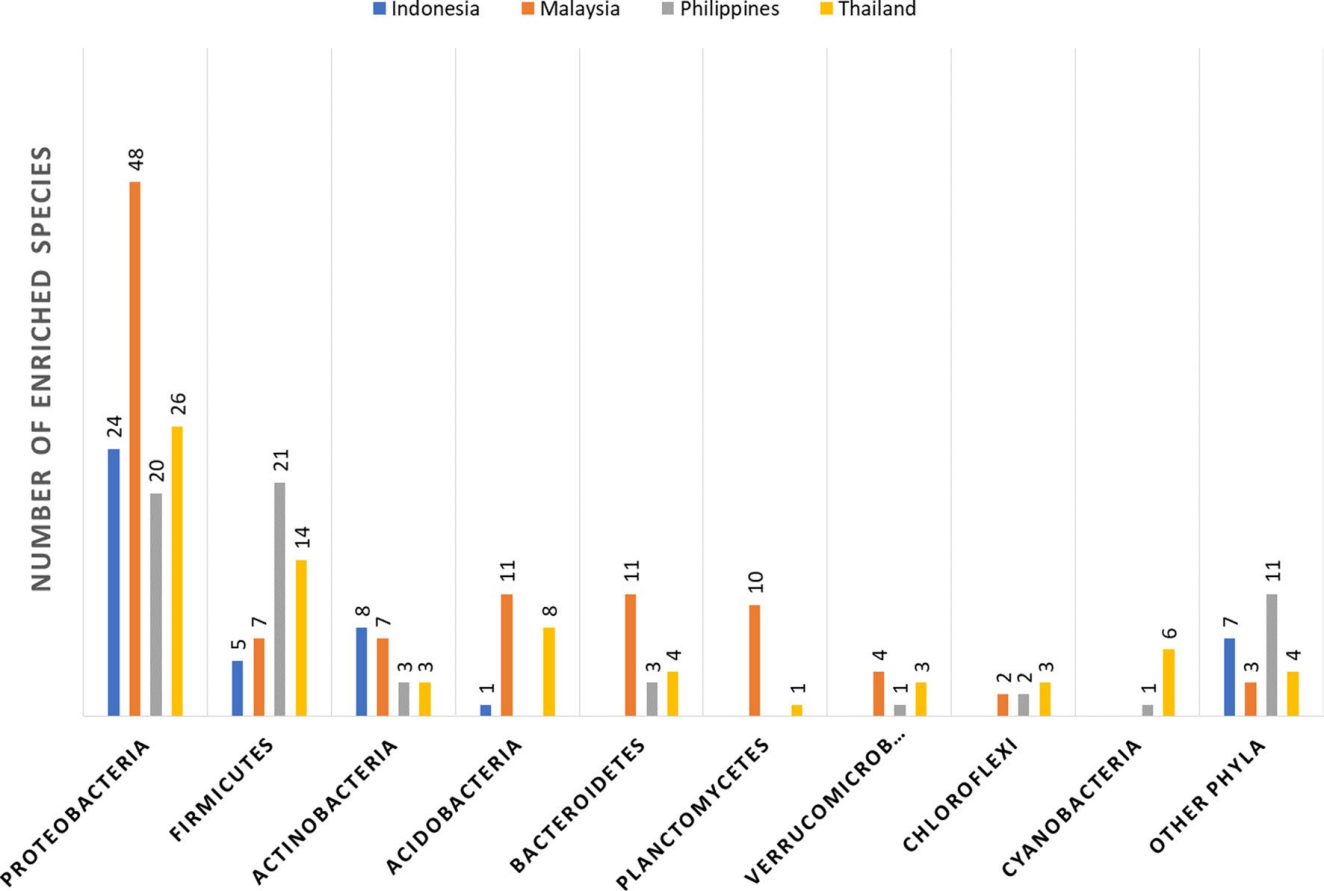
The taxonomic species richness of enriched soil bacterial species in the 4 ASEAN countries. The numbers on the bar chart represented the number of enriched soil species in different bacterial phyla.

An enriched species was defined as a species with an observed abundance in a particular sample group significantly higher than expected by random chance^22^. In this study, a total of 282 enriched soil bacterial species was found among the 4 countries. The enriched bacterial species taxonomically belonged to 25 phyla, 40 classes, 72 orders, and 125 families (Supplementary Table S3). Overall, Proteobacteria and Firmitcutes was the largest taxon groups of the enriched bacterial species in all 4 countries (Fig. 4), constituting 41.84% (118 out of 282 species) and 16.67% (47 out of 282 species), respectively.

The numbers and taxonomic distribution of the enriched species differed from country to country. Some bacterial taxa were specifically prevalent in the enriched species set of particular countries. For instance, there were 7 enriched species of Chitinophagaceae, 5 enriched species of Paenibacillaceae, and 5 enriched species of Isosphaeraceae in Malaysia while the members of these families were not present in the enriched species set of the other 3 countries (Supplementary Table S3). The uneven taxonomic proportion of the enriched species set was also observed at the phylum level as shown in Fig. 4.

### Interspecific association of bacteria in the soil bacteriomes

**Figure 5:**
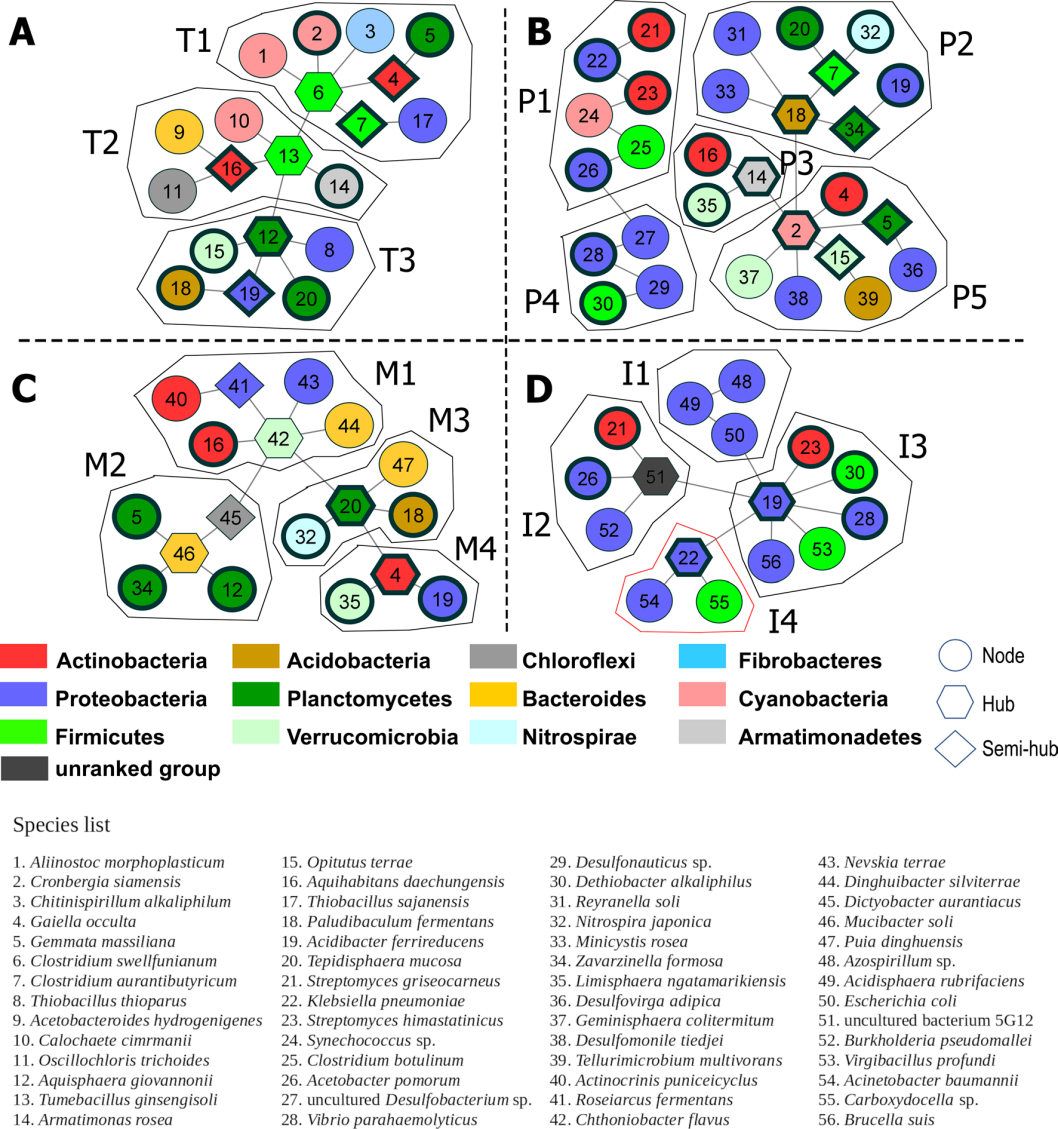
The association network of the most abundant bacterial species in the soil samples from in Thailand (A), Philippines (B), Malaysia (C), and Indonesia (D). The hexagon-shaped node represented the hub species and the diamond-shaped ones represented semi-hub species. The nodes with thick border indicated common species that appeared in more than one country. The list under the networks show the list of the bacterial species and their corresponding numbers in the networks.

For each of the selected country, the communities in the association networks of the top abundant bacterial species were identified as shown in Fig. 5.

The total 56 soil bacterial species in the association networks of all 4 countries belonged to diverse taxa (12 phyla, 24 classes, 35 orders, 42 families) (Supplementary Table S4-S7). Out of these 56 species, Proteobacteria was the most prevalent taxon group with 19 species, followed by Firmicutes (7 species). At the family level, in Thailand, Malaysia, and the Philippines, some of the bacterial families to which the species in the association networks belong were the differential families (based on species richness): 6 out of 17 families in Thailand network, 4 out of 14 families in Malaysia, and 10 out of 22 families in the Philippines (Supplementary Table S1, S4-S6). However, none of 14 bacterial families in the association network of Indonesia were differential taxa (Supplementary Table S1, S7). These differentiated taxonomic distribution may result from the heterogeneity of soil class in Indonesia soil samples while the soil samples in other three countries were more homogeneous, i.e., dominated by a single soil class for each country.

Based on the interaction degrees of the species as described in the method section, the association networks included hub and/or semi-hub species depending on the community structure.

However, the interaction degree of species did not relate to the taxonomic group to which that species belongs. Neither significant correlations between interaction degree and local abundance of the species could be found. Even when considering the same species, the interaction degree in the association network seemed to vary from country to country. For example, *Acidibacter ferrireducens* (Gammaproteobacteria), which was the only species commonly found in all 4 countries, was the hub species of the community I3 in Indonesia (degree = 8, betweenness = 93) and the semi-hub of the community T3 in Thailand (degree = 2, betweenness = 18), but had low interaction degrees in Malaysia’s community M4 and Philippines’ community P2 (degree = 1, betweenness = 0) (Fig. 5). This pattern indicates that the interaction of bacteria in the association network along their distribution range can be adaptively altered by external factors, such as surrounding environment and co-occurring microbial species.

### The soil bacteriomes and biogeochemical cycles

**Figure 6:**
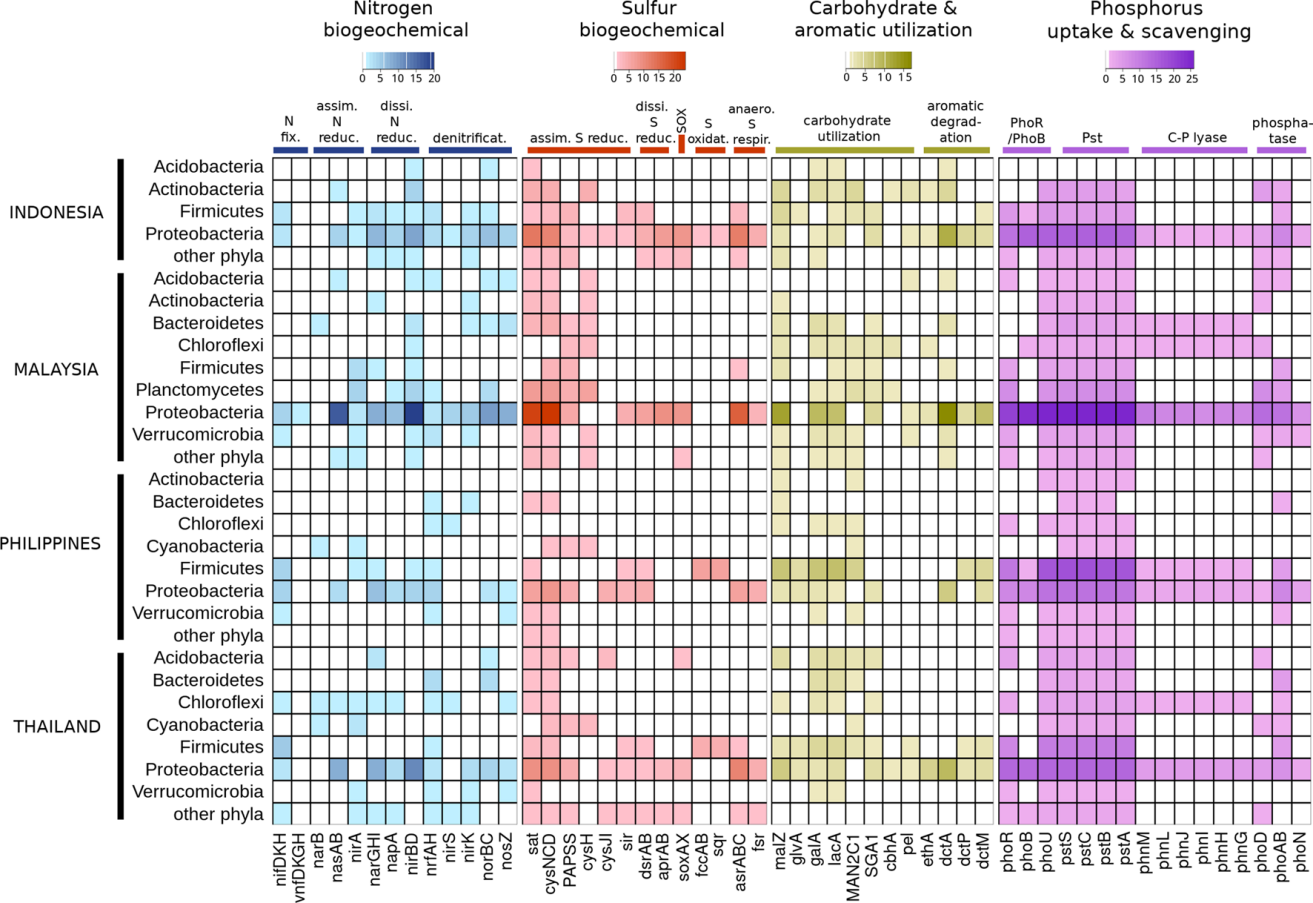
The heat map showing the numbers of soil enriched bacterial species harboring necessary genes in the metabolic processes of nitrogen, sulfur biogeochemical pathways, carbohydrate and aromatic compound utilisation, and phosphorus uptake in each of the selected country grouped by phylum. The color intensity of each cell represented the number of species.

Using the genome data of 164 enriched soil bacterial species available in the NCBI Genome Database, the functional profiles related to biogeochemical cycles of N, S, C, and P were analysed as displayed in Fig. 6.

#### Nitrogen biogeochemical pathway

The variation was observed in the association networks of the top abundant soil species (Fig. 5 and Supplementary Table S8). Most of the nitrogen-fixing bacterial species in the association networks of top abundant species belonged to Firmicutes (Supplementary Table S8). Exceptionally, in the Malaysia’s association network, there were no Firmicutes species present (Fig. 5). The only nitrogen-fixing species in the Malaysia’s association network was *Roseiarcus fermentans*, which belongs to the phylum Proteobacteria.

According to Fig. 6, nitrogen metabolic profiles also varied depending on the enriched species in the soil bacteriomes of the countries. There were 27 enriched species of nitrogen-fixing bacteria in this study, all of which encompassed the molybdenum-dependent nitrogenase genes (*nifD*, *nifK*, *nifH*, and *anfG*) (Supplementary Table S8). Thus, it is clear that the molybdenum is crucial in bacterial nitrogen fixation^23^. However, one nitrogen-fixing species, *Rhodopila globiformis* (Proteobacteria)*,* also contained the genes that encoded vanadium-dependent nitrogenase (*vnfD*, *vnfK*, *vnfG*, *vnfH*). The presence of the alternative nitrogenase might result from the limited amount of molybdenum in the environments^24,25^.

In the assimilatory nitrate reduction process, the primary nitrate reductase gene, *nasAB* was found in 36 enriched soil bacterial species, most of which belonged to Proteobacteria (Supplementary Table S8), while the alternative nitrate reductase, *narB* were found in 4 non-Proteobacteria species. Likewise, the nitrite reductase gene, *nirA* were found in 20 enriched bacterial species (Supplementary Table S8), while another nitrite reductase gene, NIT-6, was not found in any enriched bacterial species in this study (Fig. 6). In contrast, every community of the top-abundant soil networks encompassed species containing genetic capabilities to produce essential enzymes for dissimilatory nitrate and nitrite reduction (Fig. 5 and Supplementary Table S8). Even in the cases where a single species had genetic machinery to carry out only dissimilatory nitrate or nitrite reduction, the missing genes would be compensated by another co-occurring species. For example, *Gaiella occulta*, which had the dissimilatory nitrate reductase genes (*narGHI*) but lacked the dissimilatory nitrite reductase genes (*nirBD* and *nrfAH*), co-occurred in the same communities with other soil bacterial species with nitrite reductase genes, namely *Limisphaera ngatamarikiensis* (Verrucomicrobia) in the community M4 in Malaysia and *Geminisphaera colitermitum* (Verrucomicrobia) in the community P5 in the Philippines.

Only a few communities in the top abundant soil networks encompassed complete gene sets for the denitrification, in which nitrite is successively converted to nitric oxide, nitrous oxide, and ultimately nitrogen gas. The denitrification in each community was observed to proceed by a single species with all essential denitrification enzymes; for example, *Thiobacillus thioparus* (Proteobacteria) in Thailand’s community T3, *Burkholderia pseudomallei* (Proteobacteria) and *Brucella suis* (Proteobacteria) in Indonesia’s communities I2 and I3. In the Philippines’ network, denitrifying bacteria in the community P5 did not possess all key genes in denitrification; some species possessed nitrite reductase (*nirS* and *nirK*) genes while another possessed nitrous-oxide reductase (*nosZ*) gene (Fig. 5 and Supplementary Table S8). In this community, there was *Desulfomonile tiedjei* (Proteobacteria), which harbored nitric oxide reductase (*norB* and *norC*) genes. The co-occurrence of complementary species seemed to indicate that *D. tiedjei* might function as a bridge in order to complete the denitrification process.

#### Sulfur biogeochemical pathway

In accordance with the nitrogen reduction pathway, the variation of sulfur metabolic profiles of the soil bacteriomes among the 4 selected Southeast Asian countries also apparently reflected the differences in enriched species and their taxonomy (Fig. 6). There were 154 enriched soil bacterial species which possessed at least one gene in an assimilatory sulfate reduction process (Supplementary Table S9). Assimilatory sulfate reduction is a pathway to convert inorganic sulfate to sulfide, which is a form of sulfur necessary to form cysteine and homo-cysteine^26^. While several Proteobacteria enriched species were found to be genetically capable of assimilatory sulfate reduction, the Alphaproteobacteria species enriched in Thailand uncharacteristically lacked the key ATP sulfurylase genes (*cysNC* and *cysH*).

125 enriched bacterial species possessed genes for dissimilatory sulfate reduction, which is a form of anaerobic respiration that uses sulfate as the terminal electron acceptor (Supplementary Table S9). The taxonomic diversity of these species was similar among the countries, as a majority belonged to Proteobacteria. However, in terms of abundance, the enriched species with those genes had significantly high abundance in Indonesia and Thailand, and significantly low abundance in Malaysia (ANOVA, *P-*value < 0.001).

Almost every community encompassed the genes necessary to carry out the assimilatory and dissimilatory sulfate reduction, except the community T2 in Thailand that lacked genes involving in sulfate reduction entirely (Fig. 5 and Supplementary Table S9). The only sulfur functional gene found in the available genomes in T2 was *soux*, which regulates sulfite detoxification. However, there were only 3 communities that encompassed the necessary gene set to complete dissimilatory sulfate reduction due to the low number of species possessing alkaline proteases (AprAB) among the association networks. The *aprA* and *aprB* genes were found in the genomes of *Thiobacillus thioparus* (Proteobacteria) in Thailand’s community T3, *Reyranella soli* (Proteobacteria) in the Philippines’ community P2, and *Acidisphaera rubrifaciens* (Proteobacteria) in Indonesia’s community I1 (Fig. 5 and Supplementary Table S9).

Among the enriched bacterial species in this study, 24 species possessed *soxA* and *soxX*, which are essential genes in the first step of the sulfur oxidation (SOX) system that converts sulfur to sulfate through sulfide or thiosulfate and SoxYZ-S-SH complex^27,28^ (Supplementary Table S9). The enriched species with *soxAX* was significantly more abundant in Indonesia than in Thailand and Malaysia (ANOVA, *P-*value = 0.0003). However, no enriched bacterial species in the Philippines harbored SOX system-related genes. Remarkably, no enriched bacterial species possessed *soxB*, *soxC*, and *soxD*, which are genes involving in the electron release process in the SOX system. Moreover, SOX system-related genes were considerably rare in the association networks of the top abundant soil species (Fig. 5 and Supplementary Table S9). Even in Indonesia’s network where every community possessed anaerobic sulfate-respiring species, only the community I1 harbored the bacterial species with SOX system-related genes, *Acidisphaera rubrifaciens*.

There were also 11 enriched soil bacterial species harboring *fccAB* and *fsr* genes in the sulfur oxidation pathway using ferricytochrome c or quinone as an electron acceptor (Supplementary Table S9). Although the abundance of sulfur-oxidizing species was not significantly different across Thailand, Indonesia, and Philippines (ANOVA, *P *value = 0.12), the taxonomic composition was considerably different across the countries (Fig. 6). In Thailand and the Philippines, most of the enriched sulfate-oxidizing species belonged to Order Eubacteriales (Phylum Firmicutes). In Indonesia, *Salmonella enterica* (Proteobacteria) was the only enriched sulfate-oxidizing species.

#### Utilisation of carbohydrates and aromatic compounds

There were 113 enriched soil bacterial species that possessed at least one gene encoding carbohydrate-utilising enzymes (alpha-glucosidase, beta-glucosidase, alpha-galactosidase, beta-galactosidase, alpha-mannosidase, cellobiohydrolase, and pectate lyase) (Supplementary Table S10). These carbohydrate-utilising species mostly belonged to Proteobacteria and Firmicutes which were the most common phyla in the soil bacteriomes. However, some taxonomic groups seemingly exerted specificity to particular types of oligosaccharides and geographical environment. For example, the majority of the enriched species with *malZ* gene in Thailand and Malaysia soil bacteriomes belonged to Proteobacteria, whereas, in the Philippines and Indonesia, this gene was predominantly found in Firmicutes species (Fig. 6).

In terms of degradation of aromatic compounds, there were 13 enriched species harboring at least one gene involving in hydroxylation of aromatic compounds (*ethA*, *nagH*, and *nagG*) (Supplementary Table S10). Most of these aromatic compound-degrading bacteria belonged to Phylum Proteobacteria, especially *Burkholderia* and *Paraburkholderia* spp. Nonetheless, 71 enriched species possessed genes in *dct* family (*dctA*, *dctP*, and *dctM*), which encodes tripartite ATP-independent periplasmic (TRAP) C4 dicarboxylate transporters. TRAP C4 dicarboxylate transporters are the key regulator of C4 dicarboxylate movement across cellular membranes^29^. The most common *dct* gene in the enriched soil bacteria was *dctA*, which was found in 55 enriched species (Fig. 6). Most of the enriched soil species with the *dctA* gene (43 out of 55) belonged to Phylum Proteobacteria. Some Proteobacteria species even possessed 3–5 copies of the gene in their genomes (Supplementary Table S10).

#### Phosphorus uptake and scavenging

The main bioavailable form of phosphorous for soil bacteria is the orthophosphate anion (PO_4_^3-^), commonly known as inorganic phosphate (P_i_)^30^. The major P_i_ uptake mechanism in the soil bacteriomes was regulated by PhoR/PhoB two-component system which is a signal transduction controlling gene transcription for phosphorus assimilation and phosphorus scavenging^29^. There were 118 enriched soil species harboring *phoR* and/or *phoB* genes (Supplementary Table S11). PhoR/PhoB-containing species did not show taxonomic preponderance specific to the countries. All enriched Actinobacteria species, however, did not possess *phoR* or *phoB* in their genomes (Fig. 6). In terms of comparative abundance, the distribution of the species with *phoR* gene did not significantly differ across the countries (ANOVA, *P *value = 0.002) whereas the enriched species with *phoB* had significantly larger abundance in Indonesia soil bacteriomes than in other countries (ANOVA, *P-*value = 1.4E−12).

Along with the PhoR/PhoB system, Pst transporter is essential for detecting P_i_, and thus effectively regulating P_i_ uptake^30^. 149 enriched soil bacterial species harbored at least one gene of *pstSCAB* operon in their genomes (Supplementary Table S11). The prevalence of the *pst* genes were not significantly different between the countries (Fig. 6). In addition, another major regulator of the PhoR/PhoB is PhoU, which functions as PhoR/PhoB inhibitor protein. Again, 141 enriched species with the *phoU* gene were distributed evenly across the countries, signifying the universality of Pst and PhoU as essential PhoR/PhoB regulators in the soil bacteriomes^31^.

Phosphatase genes often act downstream of the PhoR/PhoB system for the hydrolysis of phosphoric esters. Among the P_i_ scavengers, most of the phosphatase genes found in the enriched soil bacteria were the alkaline phosphatases (*phoD* and *phoAB*) (Fig. 6). There were 42 and 65 enriched species harboring *phoD* and *phoAB*, respectively (Supplementary Table S11). In contrast, the acid phosphatases (*phoN*) were a less common group of phosphatases in the enriched soil species, as there were only 14 enriched species possessing the *phoN* gene.

C-P lyase pathway is a system to uptake phosphorus from C-P compound, especially phosphonates, through carbon-phosphorus bond cleavage^32^. With regards to genes in the C-P lyase core complex, 23 enriched soil species were shown to harbor *phnM, phnL, phnJ, phnI, phnH,* and *phnG* (Supplementary Table S11). According to Fig. 6, most C-P lyase-capable species belonged to Proteobacteria, Firmicutes, and Chloroflexi.

## Discussion

Due to the complex nature of microbial interactions, it is difficult to interpret relevant knowledge directly from raw metagenomic data, even for a single microbiome, let alone a pool of data collected from independent studies^3,33^. Hence, in this study, a series of analyses were employed systematically to uncover the ecological importance of soil bacteriomes in the selected four Southeast Asian countries. Firstly, species richness estimation and alpha diversity showed that Thailand’s soil bacterial diversity is significantly higher than that in the other three countries (Fig. 3B,C). This result suggests that the soil microbial diversity can be affected by geographic environments. However, it has been reported that the association between geographic distances and microbial diversity is rather weak due to the prevalence and broad dispersal range of microbes^34,35^. Therefore, in this study, the ecological effect of geographic factors on the soil bacteriomes was investigated from the perspectives of taxonomic composition and functional diversity^36,37^.

Comparing the richness of bacterial taxa in the Southeast Asian soil bacteriomes with globally known bacterial taxonomy in the LPSN systematic database, several differential bacterial lineages were detected. The diversification seemed to be comparatively high or low in a geographically specific manner. For example, Flavobacteriaceae manifested high genus richness in Thailand and unusually low genus richness in Indonesia’s soil microbiomes. Or when considering species richness, as Desulfobulbaceae, Syntrophaceae, and Syntrophobacteraceae, manifested differentially high species richness in Philippines soil bacteriome but differentially low species richness in the soil bacteriomes of Thailand, Malaysia, and Indonesia. This differential taxonomic richness may imply the effect of local environments on the divergence of microbial assemblages as suggested by Martiny and coworkers^38^. As the soil samples of the selected countries showed different in soil classes and pH levels to one another, the effects of these soil characteristics might be among the major drivers of differentiated species distributions in soil bacteriomes^39^. In addition, we observed that the co-occurrence pattern of the enriched bacterial soil species represented the prevalent interspecific association networks in the soil microbiomes which were apparently different among the countries (Fig. 5). Common species in the association networks were found to have different statuses and associated species in different countries. For example, *A. ferrireducens* was the hub species in Indonesia and semi-hub species in Thailand, but it connected to only a single species in Malaysia and the Philippines (Fig. 5). According to these observations, it can be hypothetically speculated that the microbial biodiversity emerged from stochastic events of migration and niche occupation of different species in a particular location^39–41^. Diffuse coevolution, a phenomenon in which the coevolution of two species is altered by other co-occurring species in the community, then drives the bacterial communities to exert unique characteristics in terms of species composition and interspecific interaction^42^.

Microbial diversity is known to be positively correlated to functional diversity and niche dimensions^43–45^. According to our results, the variation of functional diversity was present in the soil bacteriomes of different countries and different bacterial association networks. For example, *Rhodopila globiformis*, the acidophilic anoxygenic phototrophic purple bacterium that requires sulfate sources to survive^46^ and the only enriched species that has vanadium-mediated nitrogenases, was strongly enriched in Malaysia’s soil bacteriome. The higher-than-expected prevalence of *R. globiformis* might indicate the unique nitrogen-fixation activities in Malaysia’ soil samples. The effect of soil characteristics was also observed in the P-related functional profile. Compared to the other country’s soil samples, the bacteriome of Indonesia soil samples in which the major soil class was P-limited Nitisol distinctly contained a smaller number of phosphorus-uptake-and-scavenging bacterial species. Along with this kind of relationship pattern between locality and functional diversity, there was a total absence of cellobiose- and pectate-degrading genes among the Philippines’ enriched soil bacterial species (Fig. 6). Significantly high pH in the Gleysol-dominated Philippines soil samples might indicate the washout of plant-based biomass resulting from water erosion or topsoil erosion^48,49^.

Even for the conserved functions that are commonly present in all countries, the same metabolic processes can be performed by taxonomically-distinct species. One of the most pronounced examples is the convergent evolution of nitrite reductase (*nirK* and *nirS*) genes^50^. In Thailand, Malaysia, and Indonesia, Proteobacteria enriched species harboring either *nirK* or *nirS* were found, but there were no Proteobacteria species with nitrite reductase genes enriched in the Philippines (Fig. 6). Several studies have conferred that the nitrite reduction mechanisms of *nirK* and *nirS* are not equivalent, thus the distribution of *nirK* and *nirS* species in soil bacteriomes is constrained by ecotypes and environmental factors^51,52^.

Similar taxonomic variation of functional species was also observed in the enzymatic activities to metabolize the biogenic compounds, such as carbohydrates and phosphonates. The carbohydrate degradation by bacterial alpha- and beta-glucosidases in the Philippines’ soil bacteriome seemed to be dominated by Firmicutes species, whereas this bacterial activity in Malaysia’s soil was taken primarily by Proteobacteria species (Fig. 6). For C–P lyase pathway which metabolizes phosphonates, Proteobacteria and Chloroflexi species were the major players in Malaysia’s and Thailand’s soil bacteriomes. However, in the Philippines, the C–P lyase-capable species belonged exclusively to Proteobacteria and Firmicutes (Fig. 6). This observation supports that there is the functional redundancy in the microbiomes, i.e., multiple taxonomically-distinct species are able to perform the similar or same metabolic function^53^. The effect of functional redundancy may help stabilize microbial communities against fluctuating biotic and abiotic environments^44,54–56^. For instance, it has been suggested that the variation in C-P bond-cleavage capabilities of different bacterial communities emerges from an adaptation of co-existing species therein to cope with various types of phosphonates in the environments^57,58^.

Apart from the functional redundancy, the co-occurrence patterns of soil bacterial species can also imply the cooperative interactions and the functional complementarity in the microbiomes^59,60^. Multispecies microbial interactions are often required to drive metabolic pathways in biogeochemical cycles^14^. The co-occurrence of functionally-complement species might help optimize nutrient utilisation, leading to maximum ecological productivity^61^. As seen in the association network of the Philippines’ soil bacteriome, the occurrence of *Desulfomonile tiedjei*, which harbors nitric oxide reductase, crucially rendered the complete denitrification process in the community P5 that otherwise encompassed only nitrite reductase and nitrous-oxide reductase (Fig. 5 and Supplementary Table S8). However, the scope of this study did not include the effect of genetic constraints and horizontal gene transfer (HGT) which can result in various phenotypic adaptations of the same bacterial species in different environments and communities^62–64^. In addition, further investigation is indeed needed in order to explicitly interpret the causal relationship between evolutionary events (such as natural selection, genetic drift, and founder effect) and the functional interaction in the bacterial communities^65,66^.

Lastly, our study has pointed out the importance and current challenges in meta-analysis study of microbiomes. Our findings evidently supported that the differences in geographic locality and soil characteristics (soil class and pH) affected soil bacteriomes in terms of taxonomic diversity, interspecific association patterns, and functional diversity. Combination of data integration and bioinformatics approach bring about more information that can be translated to answer how the ecosystem is impacted by microbial activities^67,68^. Because the highly-dynamic nature of microbes consequently leads to spatial and temporal variation in microbiome composition, the multi-dimensional analyses are crucially needed to gain such meaningful insights from microbiome data^61^. Nevertheless, technical concerns about an integrated study of microbiomes are sampling biases in metagenomic methodology and systematic biases in data processing (such as data normalization, the selection of diversity measures and indices, and algorithms in data analysis)^69–71^. These biases often result in overlooking of rare or cryptic microbes. This “missing diversity” of rare taxa might be important for structuring soil communities and contributing to functional diversity^61,72^.

## Methods

### Soil microbiome data acquisition and extraction

The microbiome experiment samples were downloaded from the NCBI Bioproject database on 20 February 2020. The search was performed via Entrez API looking for any biosample records in the BioSample database (https://www.ncbi.nlm.nih.gov/biosample) which were wholly or partially matched the keywords: “Microbial community”, “Bacterial community”, “Metagenome”, “Metagenomic”, “Microbiome”, “Microbiota”. Then the search results were filtered to select only the ones which collected bacterial sequences in the environmental habitats including soil, freshwater, and wastewater & sludge. The search results were further filtered to include only the ones indicated any of Southeast Asian country names in the “geographic location” tag. The environmental information was assessed by looking up keywords in the tags “BioSampleModel”, “BioSampleTaxon”, “env_biome”, “env_feature”, and/or “env_material”.

The metadata and sequence read data of each selected biosample were fetched from the NCBI’s SRA database (https://www.ncbi.nlm.nih.gov/sra) using Entrez API^73^. The XML-formatted metadata were extracted and re-organized into a tabular format for a relational database. Geographic latitude and longitude coordinates of biosamples were plotted on the “Southeast Asia” map using Google Maps^74^. These biosamples were classified into environmental microbiome groups by keywords in the metadata. These biosamples were classified into environmental microbiome groups by keywords in the metadata. 614 biosamples from 47 soil-based microbiome projects spanning 6 Southeast Asian countries were selected. The soil biosamples from Cambodia and Singapore were excluded from the comparison due to small sample sizes (< 50 samples) compared to the other 4 countries. As a result, the comparison of soil bacteriomes in this study performed on the dataset of 558 soil biosamples from Thailand, Philippines, Malaysia, and Indonesia. The operational taxonomic unit (OTU) analysis and annotation of microbiome samples used in this study can be downloaded from https://www.amibase.org/microbiome_download.php.

### Retrieval of soil characteristics information

The latitude–longitude coordinates of the soil biosamples were used to query the soil class and pH information were retrieved from SoilGrids via the REST API web service (https://rest.soilgrids.org/)^21^. The soil classification in SoilGrids was based World Reference Base (2016) Soil Groups. The soil pH levels were estimated from the topsoil at the 0–5 cm depth. ANOVA test was performed to test significant differences in soil pH among the 4 countries.

### OTU identification

FASTQ files of SRA biosamples were entered into Kaiju taxonomic classification pipeline (Kaiju 1.7.0, minimum sequence match length = 11, minimum match score = 65)^75^ in combination with NCBI nr and KRAKEN 16S rRNA sequence databases^76^. The bacterial taxonomic classification was primarily based on NCBI taxonomy with some manual modification in order to reconciled with LPSN classification^77^.

### Calculation of bacterial species richness and diversity indices

Species richness and alpha diversity were analysed with the R package “vegan” version 2.5-5^78^. Chao1 species richness estimator and Shannon’s biodiversity index were calculated for each biosample. The Chao1 richness estimator was transformed with the log10 function. ANOVA with Tukey HSD post-hoc pairwise comparison was employed to compare log_10_(Chao1) and Shannon’s index between habitat clusters. The *P-*value threshold for pairwise comparison was 0.001.

### Bacterial taxonomic richness comparison

The taxonomic richness of a bacterial family or genus in the selected countries was estimated as the number of child taxa (members in lower taxonomic ranks belonged to that family or genus). The differences in taxonomic richness between countries were analysed using GNEISS, which detected the balance shifts in hierarchical trees^79^. Ordinary least squares (OLS) regression was employed to assess the statistical significance of the balance shift. The differential taxon was defined as a taxon (family or genus) whose balance shift was significant (*P* value < 1E−6) in all 4 selected countries. GNEISS and OLS regression were performed using QIIME2 version 2019.10^80^.

### Species enrichment analysis

A data matrix of species OTU abundance (read counts) and samples was prepared and imported to R version 3.6.1^81^. The read counts of all OTUs of the same species were summed into a single species read count. Species read counts were normalized using GMPR normalization as there were inflated zeroes in the data matrix^82^. The species enrichment analysis was performed using chi-squared tests. An enriched taxon is an “over-represented” taxon whose observed value of abundance in a particular country is significantly higher than an expected value by random chance (Pearson's Chi-squared test, adjusted *P* value < 1e-6). The Pearson’s residuals of each enriched taxon were calculated by subtracting the normalized observed value with the expected value.

The species was considered enriched in the country when the Pearson’s abundance residual of the species in that country was a positive outlier. In this case, the outlier was defined as the value higher than the third quartile plus interquartile range (IQR = Q3−Q1).

### Bacterial community structure of the association networks

To uncover the frequent interspecific association of abundant bacterial species in soil bacteriomes, the top-20 abundant species from each soil sample were selected and treated as co-occurring items in a sample. Association analysis was performed using the apriori method in R package “arules” version 1.6^83^. The interspecific association pairs of the bacterial species were selected with the thresholds of support and confidence values higher than 0.3 and 0.7, respectively. The association network in each country was constructed and visualized as an undirected graph from the list of association pairs from the samples in that country.

As minimum spanning tree (MST) finds the shortest path to connects all the nodes together without any cycles and with the minimum possible total edge weight, it was employed to assume the direct relationships between species in the association networks^33^. the community structure of the MST association network was detected using Girvan-Newman’s algorithm based on the edge betweenness which assumes that edges connecting separate clusters have high edge betweenness as all between-cluster paths from every node in the modules must traverse through them^84^. The network construction, MST, and community structure detection were performed in the R igraph version 1.2.4^85^.

The node degree (number of edges connected to the species node) and betweenness (number of internode paths which transverse through the species node) of species in the association network were used to infer the interaction degree at which the species interacted to other species in the network. Using the interaction degrees of the species in the communities, “hub” species is defined as a species which has the highest interaction degree; and “semi-hub” species is one which connected to more than 2 other members but does not have the highest interaction degree in its community.

### Functional annotation of the bacterial species

In order to infer the functional diversity of soil bacteriomes, FASTA files of the proteomic sequences of the enriched bacterial species were downloaded from the NCBI Genome database (https://www.ncbi.nlm.nih.gov/genome/). The proteomic FASTA files were selected based on the RefSeq category or, if the former case was not applicable, the assembly level. The priority of selection was in the order of reference, representative, type specimens, complete genome, chromosome, scaffold, and contig. In total, the functions of proteins from 164 enriched bacterial species were annotated. The protein sequences were annotated by profile-matching against KEGG HMM protein profiles using Kofamscan 1.0.0^86^.

N and S metabolic pathways in this study were adapted from those in the KEGG pathways “ko00910 nitrogen metabolism” and “ko00920 sulfur metabolism”, respectively^87^. C genes were derived from carbohydrate- and aromatic-degrading enzymes listed in Hartman et al.’s study^16^. P genes were selected from the KEGG genes involved in the PhoR/PhoB two-component system (and its downstream enzymes), Pst transporters, and C-P lyase pathway^18^. The functional genes and their KEGG ortholog numbers were listed in Supplementary Table S12.

### Comparison of functional species abundance between countries

The abundance of the enriched species was used as a proxy to compare the prevalence of metabolic functions across different countries. GMPR-normalized values of read counts of all enriched species which possessed the function-related genes in each sample were summed together. ANOVA was employed to test the statistical significance of between-country differences of those sum abundance values. The significant differences were cut off at the threshold of *P *value < 0.001.

## Supplementary Information

Supplementary Information.

## Data Availability

The dataset used in this study including the habitat information of microbiome samples and bacterial species can be found at the AmiBase website https://www.amibase.org/microbiome_download.php.
