# Supplementary material for: Uncovering multi-faceted taxonomic and functional diversity of soil bacteriomes in tropical Southeast Asian countries
Source: Sci Rep. 2021 Jan 12;11:582. doi: 10.1038/s41598-020-79786-x (PMC7804445; doi:10.1038/s41598-020-79786-x)
Supplement: Supplementary file 1 — Supplementary Information. [file 41598_2020_79786_MOESM1_ESM.docx]

**SUPPLEMENTARY INFORMATION**

**Uncovering multi-faceted taxonomic and functional diversity of soil bacteriomes in tropical Southeast Asian countries**

Somsak Likhitrattanapisal^1^, Paopit Siriarchawatana^1^, Mintra Seesang^1^, Suwanee Chunhametha^1^, Worawongsin Boonsin^1^, Chitwadee Phithakrotchanakoon^1^, Supattra Kitikhun^1^, Lily Eurwilaichitr^1^ *, Supawadee Ingsriswang^1^ *

^1^Thailand Bioresource Research Center (TBRC), National Center for Genetic Engineering and Biotechnology (BIOTEC), National Science and Technology Development Agency (NSTDA), Pathumthani, Thailand

* Corresponding authors

E-mail: [lily@biotec.or.th](mailto:lily@biotec.or.th)

E-mail: [supawadee@biotec.or.th](mailto:supawadee@biotec.or.th)

**Supplementary Figures**

**Supplementary Figure S1: The bacterial taxonomic composition of the soil samples from the selected 4 Southeast Asian countries**


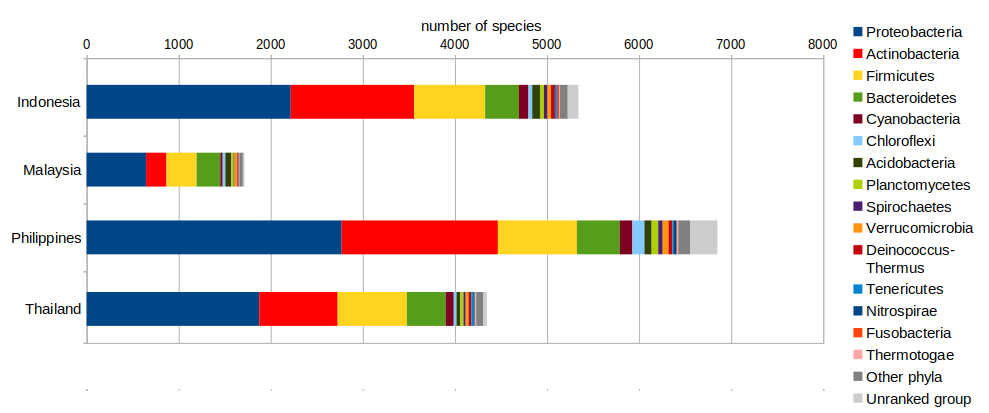


**Supplementary Figure S2: The soil classess composition of the bacteriome samples from the selected 4 Southeast Asian countries.**


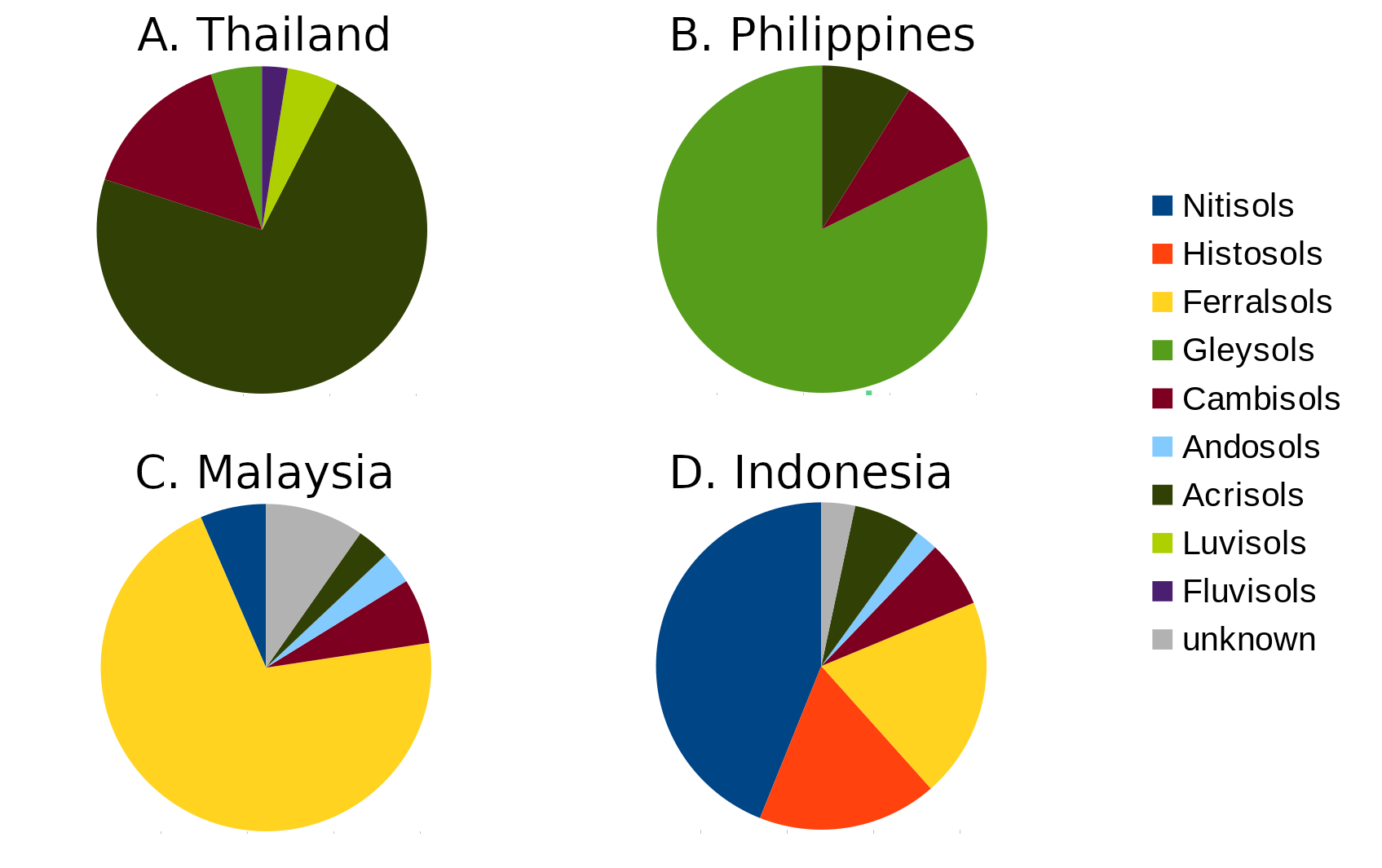


**Supplementary Tables**

**Supplementary Table S1: The differential families of soil bacteria in the selected ASEAN countries** (Gneiss and OLS regression, *P*-value < 1E-6)

**A. The differential families calculated based on numbers of member genera**

| **Family** | **Phylum** | **Class** | **Order** |
| --- | --- | --- | --- |
| Acholeplasmataceae | Tenericutes | Mollicutes | Acholeplasmatales |
| Acidaminococcaceae | Firmicutes | Negativicutes | Acidaminococcales |
| Acidiferrobacteraceae | Proteobacteria | Gammaproteobacteria | Acidiferrobacterales |
| Acidimicrobiaceae | Actinobacteria | Acidimicrobiia | Acidimicrobiales |
| Acidithiobacillaceae | Proteobacteria | Acidithiobacillia | Acidithiobacillales |
| Acidobacteriaceae | Acidobacteria | Acidobacteriia | Acidobacteriales |
| Acidothermaceae | Actinobacteria | Actinobacteria | Acidothermales |
| Actinomycetaceae | Actinobacteria | Actinobacteria | Actinomycetales |
| Actinopolysporaceae | Actinobacteria | Actinobacteria | Actinopolysporales |
| Actinospicaceae | Actinobacteria | Actinobacteria | Catenulisporales |
| Aerococcaceae | Firmicutes | Bacilli | Lactobacillales |
| Aeromonadaceae | Proteobacteria | Gammaproteobacteria | Aeromonadales |
| Akkermansiaceae | Verrucomicrobia | Verrucomicrobiae | Verrucomicrobiales |
| Alcanivoracaceae | Proteobacteria | Gammaproteobacteria | Oceanospirillales |
| Algiphilaceae | Proteobacteria | Gammaproteobacteria | Nevskiales |
| Alicyclobacillaceae | Firmicutes | Bacilli | Bacillales |
| Alteromonadaceae | Proteobacteria | Gammaproteobacteria | Alteromonadales |
| Anaerolineaceae | Chloroflexi | Anaerolineae | Anaerolineales |
| Anaeromyxobacteraceae | Proteobacteria | Deltaproteobacteria | Myxococcales |
| Anaplasmataceae | Proteobacteria | Alphaproteobacteria | Rickettsiales |
| Aphanizomenonaceae | Cyanobacteria |  | Nostocales |
| Aphanothecaceae | Cyanobacteria |  | Chroococcales |
| Aquificaceae | Aquificae | Aquificae | Aquificales |
| Archangiaceae | Proteobacteria | Deltaproteobacteria | Myxococcales |
| Ardenticatenaceae | Chloroflexi | Ardenticatenia | Ardenticatenales |
| Armatimonadaceae | Armatimonadetes | Armatimonadia | Armatimonadales |
| Atopobiaceae | Actinobacteria | Coriobacteriia | Coriobacteriales |
| Aurantimonadaceae | Proteobacteria | Alphaproteobacteria | Rhizobiales |
| Azonexaceae | Proteobacteria | Betaproteobacteria | Rhodocyclales |
| Bacillales Family X. Incertae Sedis | Firmicutes | Bacilli | Bacillales |
| Bacteriovoracaceae | Proteobacteria | Oligoflexia | Bacteriovoracales |
| Bacteroidaceae | Bacteroidetes | Bacteroidia | Bacteroidales |
| Balneolaceae | Balneolaeota | Balneolia | Balneolales |
| Barnesiellaceae | Bacteroidetes | Bacteroidia | Bacteroidales |
| Bartonellaceae | Proteobacteria | Alphaproteobacteria | Rhizobiales |
| Bdellovibrionaceae | Proteobacteria | Oligoflexia | Bdellovibrionales |
| Beijerinckiaceae | Proteobacteria | Alphaproteobacteria | Rhizobiales |
| Beutenbergiaceae | Actinobacteria | Actinobacteria | Micrococcales |
| Blastocatellaceae | Acidobacteria | Blastocatellia | Blastocatellales |
| Bogoriellaceae | Actinobacteria | Actinobacteria | Micrococcales |
| Brachyspiraceae | Spirochaetes | Spirochaetia | Brachyspirales |
| Bradymonadaceae | Proteobacteria | Deltaproteobacteria | Bradymonadales |
| Bradyrhizobiaceae | Proteobacteria | Alphaproteobacteria | Rhizobiales |
| Brevibacteriaceae | Actinobacteria | Actinobacteria | Micrococcales |
| Brevinemataceae | Spirochaetes | Spirochaetia | Brevinematales |
| Bryobacteraceae | Acidobacteria | Acidobacteriia | Bryobacterales |
| Budviciaceae | Proteobacteria | Gammaproteobacteria | Enterobacterales |
| Burkholderiaceae | Proteobacteria | Betaproteobacteria | Burkholderiales |
| Caedimonadaceae | Proteobacteria | Alphaproteobacteria | Holosporales |
| Caldicoprobacteraceae | Firmicutes | Clostridia | Eubacteriales |
| Caldilineaceae | Chloroflexi | Caldilineae | Caldilineales |
| Calotrichaceae | Cyanobacteria |  | Nostocales |
| Campylobacteraceae | Proteobacteria | Epsilonproteobacteria | Campylobacterales |
| Candidatus Brocadiaceae | Planctomycetes | Planctomycetia | Candidatus Brocadiales |
| Candidatus Competibacteraceae | Proteobacteria | Gammaproteobacteria |  |
| Capsulimonadaceae | Armatimonadetes | Armatimonadia | Capsulimonadales |
| Cardiobacteriaceae | Proteobacteria | Gammaproteobacteria | Cardiobacteriales |
| Carnobacteriaceae | Firmicutes | Bacilli | Lactobacillales |
| Caryophanaceae | Firmicutes | Bacilli | Bacillales |
| Catalimonadaceae | Bacteroidetes | Cytophagia | Cytophagales |
| Catenulisporaceae | Actinobacteria | Actinobacteria | Catenulisporales |
| Caulobacteraceae | Proteobacteria | Alphaproteobacteria | Caulobacterales |
| Cellulomonadaceae | Actinobacteria | Actinobacteria | Micrococcales |
| Cellvibrionaceae | Proteobacteria | Gammaproteobacteria | Cellvibrionales |
| Chelatococcaceae | Proteobacteria | Alphaproteobacteria | Rhizobiales |
| Chitinispirillaceae | Fibrobacteres | Chitinispirillia | Chitinispirillales |
| Chitinophagaceae | Bacteroidetes | Chitinophagia | Chitinophagales |
| Chlorobiaceae | Chlorobi | Chlorobia | Chlorobiales |
| Chloroflexaceae | Chloroflexi | Chloroflexia | Chloroflexales |
| Chlorogloeopsidaceae | Cyanobacteria |  | Nostocales |
| Chromatiaceae | Proteobacteria | Gammaproteobacteria | Chromatiales |
| Chromobacteriaceae | Proteobacteria | Betaproteobacteria | Neisseriales |
| Chroococcidiopsidaceae | Cyanobacteria | Gloeobacteria | Chroococcidiopsidales |
| Chrysiogenaceae | Chrysiogenetes | Chrysiogenetes | Chrysiogenales |
| Chthoniobacteraceae | Verrucomicrobia | Spartobacteria | Chthoniobacterales |
| Chthonomonadaceae | Armatimonadetes | Chthonomonadetes | Chthonomonadales |
| Clostridiaceae | Firmicutes | Clostridia | Eubacteriales |
| Cohaesibacteraceae | Proteobacteria | Alphaproteobacteria | Rhizobiales |
| Coleofasciculaceae | Cyanobacteria |  | Oscillatoriales |
| Colwelliaceae | Proteobacteria | Gammaproteobacteria | Alteromonadales |
| Comamonadaceae | Proteobacteria | Betaproteobacteria | Burkholderiales |
| Conexibacteraceae | Actinobacteria | Thermoleophilia | Solirubrobacterales |
| Coriobacteriaceae | Actinobacteria | Coriobacteriia | Coriobacteriales |
| Corynebacteriaceae | Actinobacteria | Actinobacteria | Corynebacteriales |
| Coxiellaceae | Proteobacteria | Gammaproteobacteria | Legionellales |
| Crenotrichaceae | Proteobacteria | Gammaproteobacteria | Methylococcales |
| Criblamydiaceae | Chlamydiae | Chlamydiia | Chlamydiales |
| Crocinitomicaceae | Bacteroidetes | Flavobacteriia | Flavobacteriales |
| Cryomorphaceae | Bacteroidetes | Flavobacteriia | Flavobacteriales |
| Cryptosporangiaceae | Actinobacteria | Actinobacteria | Cryptosporangiales |
| Cyanobacteriaceae | Cyanobacteria |  | Chroococcales |
| Cyanothecaceae | Cyanobacteria |  | Oscillatoriales |
| Cyclobacteriaceae | Bacteroidetes | Cytophagia | Cytophagales |
| Cytophagaceae | Bacteroidetes | Cytophagia | Cytophagales |
| Deferribacteraceae | Deferribacteres | Deferribacteres | Deferribacterales |
| Defluviitaleaceae | Firmicutes | Clostridia | Eubacteriales |
| Dehalococcoidaceae | Chloroflexi | Dehalococcoidia | Dehalococcoidales |
| Deinococcaceae | Deinococcus-Thermus | Deinococci | Deinococcales |
| Dermabacteraceae | Actinobacteria | Actinobacteria | Micrococcales |
| Dermacoccaceae | Actinobacteria | Actinobacteria | Micrococcales |
| Dermocarpellaceae | Cyanobacteria |  | Pleurocapsales |
| Desulfarculaceae | Proteobacteria | Deltaproteobacteria | Desulfarculales |
| Desulfobacteraceae | Proteobacteria | Deltaproteobacteria | Desulfobacterales |
| Desulfobulbaceae | Proteobacteria | Deltaproteobacteria | Desulfobacterales |
| Desulfomicrobiaceae | Proteobacteria | Deltaproteobacteria | Desulfovibrionales |
| Desulfovibrionaceae | Proteobacteria | Deltaproteobacteria | Desulfovibrionales |
| Desulfurellaceae | Proteobacteria | Deltaproteobacteria | Desulfurellales |
| Desulfuromonadaceae | Proteobacteria | Deltaproteobacteria | Desulfuromonadales |
| Dictyobacteraceae | Chloroflexi | Ktedonobacteria | Ktedonobacterales |
| Dietziaceae | Actinobacteria | Actinobacteria | Corynebacteriales |
| Dysgonomonadaceae | Bacteroidetes | Bacteroidia | Bacteroidales |
| Ectothiorhodospiraceae | Proteobacteria | Gammaproteobacteria | Chromatiales |
| Eggerthellaceae | Actinobacteria | Coriobacteriia | Eggerthellales |
| Egicoccaceae | Actinobacteria | Nitriliruptoria | Egicoccales |
| Elusimicrobiaceae | Elusimicrobia | Elusimicrobia | Elusimicrobiales |
| Endomicrobiaceae | Elusimicrobia | Endomicrobia | Endomicrobiales |
| Endozoicomonadaceae | Proteobacteria | Gammaproteobacteria | Oceanospirillales |
| Enterococcaceae | Firmicutes | Bacilli | Lactobacillales |
| Erwiniaceae | Proteobacteria | Gammaproteobacteria | Enterobacterales |
| Erysipelotrichaceae | Firmicutes | Erysipelotrichia | Erysipelotrichales |
| Erythrobacteraceae | Proteobacteria | Alphaproteobacteria | Sphingomonadales |
| Eubacteriaceae | Firmicutes | Clostridia | Eubacteriales |
| Eubacteriales Family XII. Incertae Sedis | Firmicutes | Clostridia | Eubacteriales |
| Eubacteriales Family XIII. Incertae Sedis | Firmicutes | Clostridia | Eubacteriales |
| Eubacteriales Family XVII. Incertae Sedis | Firmicutes | Clostridia | Eubacteriales |
| Euzebyaceae | Actinobacteria | Nitriliruptoria | Euzebyales |
| Ferrovaceae | Proteobacteria | Betaproteobacteria | Ferrovales |
| Fibrobacteraceae | Fibrobacteres | Fibrobacteria | Fibrobacterales |
| Fimbriimonadaceae | Armatimonadetes | Fimbriimonadia | Fimbriimonadales |
| Flavobacteriaceae | Bacteroidetes | Flavobacteriia | Flavobacteriales |
| Fortieaceae | Cyanobacteria |  | Nostocales |
| Francisellaceae | Proteobacteria | Gammaproteobacteria | Thiotrichales |
| Frankiaceae | Actinobacteria | Actinobacteria | Frankiales |
| Fusobacteriaceae | Fusobacteria | Fusobacteriia | Fusobacteriales |
| Gaiellaceae | Actinobacteria | Rubrobacteria | Gaiellales |
| Gallionellaceae | Proteobacteria | Betaproteobacteria | Nitrosomonadales |
| Geminicoccaceae | Proteobacteria | Alphaproteobacteria | Rhodospirillales |
| Gemmataceae | Planctomycetes | Planctomycetia | Planctomycetales |
| Gemmatimonadaceae | Gemmatimonadetes | Gemmatimonadetes | Gemmatimonadales |
| Geobacteraceae | Proteobacteria | Deltaproteobacteria | Desulfuromonadales |
| Geodermatophilaceae | Actinobacteria | Actinobacteria | Geodermatophilales |
| Gloeobacteraceae | Cyanobacteria | Gloeobacteria | Gloeobacterales |
| Glycomycetaceae | Actinobacteria | Actinobacteria | Glycomycetales |
| Gordoniaceae | Actinobacteria | Actinobacteria | Corynebacteriales |
| Gottschalkiaceae | Firmicutes | Tissierellia | Tissierellales |
| Gracilibacteraceae | Firmicutes | Clostridia | Eubacteriales |
| Granulosicoccaceae | Proteobacteria | Gammaproteobacteria | Chromatiales |
| Hafniaceae | Proteobacteria | Gammaproteobacteria | Enterobacterales |
| Hahellaceae | Proteobacteria | Gammaproteobacteria | Oceanospirillales |
| Halanaerobiaceae | Firmicutes | Clostridia | Halanaerobiales |
| Halieaceae | Proteobacteria | Gammaproteobacteria | Cellvibrionales |
| Haliscomenobacteraceae | Bacteroidetes | Saprospiria | Saprospirales |
| Halobacteriovoraceae | Proteobacteria | Oligoflexia | Bacteriovoracales |
| Halobacteroidaceae | Firmicutes | Clostridia | Halanaerobiales |
| Halomonadaceae | Proteobacteria | Gammaproteobacteria | Oceanospirillales |
| Halothiobacillaceae | Proteobacteria | Gammaproteobacteria | Chromatiales |
| Hapalosiphonaceae | Cyanobacteria |  | Nostocales |
| Helicobacteraceae | Proteobacteria | Epsilonproteobacteria | Campylobacterales |
| Herpetosiphonaceae | Chloroflexi | Chloroflexia | Herpetosiphonales |
| Holophagaceae | Acidobacteria | Holophagae | Holophagales |
| Hydrogenophilaceae | Proteobacteria | Hydrogenophilalia | Hydrogenophilales |
| Hymenobacteraceae | Bacteroidetes | Cytophagia | Cytophagales |
| Hyphomicrobiaceae | Proteobacteria | Alphaproteobacteria | Rhizobiales |
| Hyphomonadaceae | Proteobacteria | Alphaproteobacteria | Rhodobacterales |
| Iamiaceae | Actinobacteria | Acidimicrobiia | Acidimicrobiales |
| Idiomarinaceae | Proteobacteria | Gammaproteobacteria | Alteromonadales |
| Ignavibacteriaceae | Ignavibacteriae | Ignavibacteria | Ignavibacteriales |
| Ilumatobacteraceae | Actinobacteria | Acidimicrobiia | Acidimicrobiales |
| Immundisolibacteraceae | Proteobacteria | Gammaproteobacteria | Immundisolibacterales |
| Intrasporangiaceae | Actinobacteria | Actinobacteria | Micrococcales |
| Isosphaeraceae | Planctomycetes | Planctomycetia | Planctomycetales |
| Jiangellaceae | Actinobacteria | Actinobacteria | Jiangellales |
| Jonesiaceae | Actinobacteria | Actinobacteria | Micrococcales |
| Kallotenuaceae | Chloroflexi | Chloroflexia | Kallotenuales |
| Kineosporiaceae | Actinobacteria | Actinobacteria | Kineosporiales |
| Kofleriaceae | Proteobacteria | Deltaproteobacteria | Myxococcales |
| Kosmotogaceae | Thermotogae | Thermotogae | Kosmotogales |
| Ktedonobacteraceae | Chloroflexi | Ktedonobacteria | Ktedonobacterales |
| Labilitrichaceae | Proteobacteria | Deltaproteobacteria | Myxococcales |
| Lachnospiraceae | Firmicutes | Clostridia | Eubacteriales |
| Lacipirellulaceae | Planctomycetes | Planctomycetia | Pirellulales |
| Legionellaceae | Proteobacteria | Gammaproteobacteria | Legionellales |
| Lentimicrobiaceae | Bacteroidetes | Bacteroidia | Bacteroidales |
| Leptolyngbyaceae | Cyanobacteria |  | Synechococcales |
| Leptospiraceae | Spirochaetes | Spirochaetia |  |
| Leptotrichiaceae | Fusobacteria | Fusobacteriia | Fusobacteriales |
| Leuconostocaceae | Firmicutes | Bacilli | Lactobacillales |
| Lewinellaceae | Bacteroidetes | Saprospiria | Saprospirales |
| Limnochordaceae | Firmicutes | Limnochordia | Limnochordales |
| Longimicrobiaceae | Gemmatimonadetes | Longimicrobia | Longimicrobiales |
| Lysobacteraceae | Proteobacteria | Gammaproteobacteria | Lysobacterales |
| Marinifilaceae | Bacteroidetes | Bacteroidia | Marinilabiliales |
| Marinilabiliaceae | Bacteroidetes | Bacteroidia | Marinilabiliales |
| Mariprofundaceae | Proteobacteria | Zetaproteobacteria | Mariprofundales |
| Melioribacteraceae | Ignavibacteriae | Ignavibacteria | Ignavibacteriales |
| Methylobacteriaceae | Proteobacteria | Alphaproteobacteria | Rhizobiales |
| Methylococcaceae | Proteobacteria | Gammaproteobacteria | Methylococcales |
| Methylocystaceae | Proteobacteria | Alphaproteobacteria | Rhizobiales |
| Methylophilaceae | Proteobacteria | Betaproteobacteria | Nitrosomonadales |
| Microbacteriaceae | Actinobacteria | Actinobacteria | Micrococcales |
| Microbulbiferaceae | Proteobacteria | Gammaproteobacteria | Cellvibrionales |
| Micrococcaceae | Actinobacteria | Actinobacteria | Micrococcales |
| Microcoleaceae | Cyanobacteria |  | Oscillatoriales |
| Microcystaceae | Cyanobacteria |  | Chroococcales |
| Micromonosporaceae | Actinobacteria | Actinobacteria | Micromonosporales |
| Micropepsaceae | Proteobacteria | Alphaproteobacteria | Micropepsales |
| Moraxellaceae | Proteobacteria | Gammaproteobacteria | Pseudomonadales |
| Morganellaceae | Proteobacteria | Gammaproteobacteria | Enterobacterales |
| Moritellaceae | Proteobacteria | Gammaproteobacteria | Alteromonadales |
| Motilibacteraceae | Actinobacteria | Actinobacteria | Frankiales |
| Muribaculaceae | Bacteroidetes | Bacteroidia | Bacteroidales |
| Mycobacteriaceae | Actinobacteria | Actinobacteria | Corynebacteriales |
| Mycoplasmataceae | Tenericutes | Mollicutes | Mycoplasmatales |
| Myxococcaceae | Proteobacteria | Deltaproteobacteria | Myxococcales |
| Nakamurellaceae | Actinobacteria | Actinobacteria | Nakamurellales |
| Nannocystaceae | Proteobacteria | Deltaproteobacteria | Myxococcales |
| Neisseriaceae | Proteobacteria | Betaproteobacteria | Neisseriales |
| Nevskiaceae | Proteobacteria | Gammaproteobacteria | Nevskiales |
| Nitriliruptoraceae | Actinobacteria | Nitriliruptoria | Nitriliruptorales |
| Nitrosomonadaceae | Proteobacteria | Betaproteobacteria | Nitrosomonadales |
| Nitrospinaceae | Nitrospinae | Nitrospinia | Nitrospinales |
| Nitrospiraceae | Nitrospirae | Nitrospira | Nitrospirales |
| Nocardiaceae | Actinobacteria | Actinobacteria | Corynebacteriales |
| Nocardioidaceae | Actinobacteria | Actinobacteria | Propionibacteriales |
| Nocardiopsaceae | Actinobacteria | Actinobacteria | Streptosporangiales |
| Nostocaceae | Cyanobacteria |  | Nostocales |
| Oceanospirillaceae | Proteobacteria | Gammaproteobacteria | Oceanospirillales |
| Oligoflexaceae | Proteobacteria | Oligoflexia | Oligoflexales |
| Opitutaceae | Verrucomicrobia | Opitutae | Opitutales |
| Orbaceae | Proteobacteria | Gammaproteobacteria | Orbales |
| Oscillatoriaceae | Cyanobacteria |  | Oscillatoriales |
| Oscillochloridaceae | Chloroflexi | Chloroflexia | Chloroflexales |
| Oscillospiraceae | Firmicutes | Clostridia | Eubacteriales |
| Oxalobacteraceae | Proteobacteria | Betaproteobacteria | Burkholderiales |
| Paenibacillaceae | Firmicutes | Bacilli | Bacillales |
| Paludibacteraceae | Bacteroidetes | Bacteroidia | Bacteroidales |
| Parachlamydiaceae | Chlamydiae | Chlamydiia | Chlamydiales |
| Parviterribacteraceae | Actinobacteria | Thermoleophilia | Solirubrobacterales |
| Parvularculaceae | Proteobacteria | Alphaproteobacteria | Parvularculales |
| Pasteurellaceae | Proteobacteria | Gammaproteobacteria | Pasteurellales |
| Patulibacteraceae | Actinobacteria | Thermoleophilia | Solirubrobacterales |
| Pectobacteriaceae | Proteobacteria | Gammaproteobacteria | Enterobacterales |
| Pelagibacteraceae | Proteobacteria | Alphaproteobacteria | Pelagibacterales |
| Peptococcaceae | Firmicutes | Clostridia | Eubacteriales |
| Peptoniphilaceae | Firmicutes | Tissierellia | Tissierellales |
| Peptostreptococcaceae | Firmicutes | Clostridia | Eubacteriales |
| Petrotogaceae | Thermotogae | Thermotogae | Petrotogales |
| Phaselicystidaceae | Proteobacteria | Deltaproteobacteria | Myxococcales |
| Phycisphaeraceae | Planctomycetes | Phycisphaerae | Phycisphaerales |
| Phyllobacteriaceae | Proteobacteria | Alphaproteobacteria | Rhizobiales |
| Piscirickettsiaceae | Proteobacteria | Gammaproteobacteria | Thiotrichales |
| Planctomycetaceae | Planctomycetes | Planctomycetia | Planctomycetales |
| Pleurocapsaceae | Cyanobacteria | Gloeobacteria | Pleurocapsales |
| Polyangiaceae | Proteobacteria | Deltaproteobacteria | Myxococcales |
| Porphyromonadaceae | Bacteroidetes | Bacteroidia | Bacteroidales |
| Prevotellaceae | Bacteroidetes | Bacteroidia | Bacteroidales |
| Prochloraceae | Cyanobacteria |  | Synechococcales |
| Prolixibacteraceae | Bacteroidetes | Bacteroidia | Marinilabiliales |
| Promicromonosporaceae | Actinobacteria | Actinobacteria | Micrococcales |
| Propionibacteriaceae | Actinobacteria | Actinobacteria | Propionibacteriales |
| Proteinivoraceae | Firmicutes | Clostridia | Eubacteriales |
| Pseudanabaenaceae | Cyanobacteria |  | Synechococcales |
| Pseudomonadaceae | Proteobacteria | Gammaproteobacteria | Pseudomonadales |
| Pseudonocardiaceae | Actinobacteria | Actinobacteria | Pseudonocardiales |
| Psychromonadaceae | Proteobacteria | Gammaproteobacteria | Alteromonadales |
| Puniceicoccaceae | Verrucomicrobia | Opitutae | Puniceicoccales |
| Pyrinomonadaceae | Acidobacteria | Blastocatellia | Blastocatellales |
| Rhizobiaceae | Proteobacteria | Alphaproteobacteria | Rhizobiales |
| Rhodanobacteraceae | Proteobacteria | Gammaproteobacteria | Lysobacterales |
| Rhodobiaceae | Proteobacteria | Alphaproteobacteria | Rhizobiales |
| Rhodocyclaceae | Proteobacteria | Betaproteobacteria | Rhodocyclales |
| Rhodothermaceae | Bacteroidetes | Chitinophagia | Bacteroidetes Order II. Incertae sedis |
| Rickettsiaceae | Proteobacteria | Alphaproteobacteria | Rickettsiales |
| Rikenellaceae | Bacteroidetes | Bacteroidia | Bacteroidales |
| Rivulariaceae | Cyanobacteria |  | Nostocales |
| Roseiarcaceae | Proteobacteria | Alphaproteobacteria | Rhizobiales |
| Roseiflexaceae | Chloroflexi | Chloroflexia | Chloroflexales |
| Ruaniaceae | Actinobacteria | Actinobacteria | Micrococcales |
| Rubricoccaceae | Rhodothermaeota | Rhodothermia | Rhodothermales |
| Rubrobacteraceae | Actinobacteria | Rubrobacteria | Rubrobacterales |
| Saccharospirillaceae | Proteobacteria | Gammaproteobacteria | Oceanospirillales |
| Salinarimonadaceae | Proteobacteria | Alphaproteobacteria | Rhizobiales |
| Salinisphaeraceae | Proteobacteria | Gammaproteobacteria | Nevskiales |
| Sandaracinaceae | Proteobacteria | Deltaproteobacteria | Myxococcales |
| Saprospiraceae | Bacteroidetes | Saprospiria | Saprospirales |
| Scytonemataceae | Cyanobacteria |  | Nostocales |
| Sedimentisphaeraceae | Planctomycetes | Phycisphaerae | Sedimentisphaerales |
| Segniliparaceae | Actinobacteria | Actinobacteria | Corynebacteriales |
| Selenomonadaceae | Firmicutes | Negativicutes | Selenomonadales |
| Shewanellaceae | Proteobacteria | Gammaproteobacteria | Alteromonadales |
| Silvanigrellaceae | Proteobacteria | Oligoflexia | Silvanigrellales |
| Simkaniaceae | Chlamydiae | Chlamydiia | Chlamydiales |
| Sneathiellaceae | Proteobacteria | Alphaproteobacteria | Sneathiellales |
| Solibacteraceae | Acidobacteria | Solibacteres | Solibacterales |
| Solirubrobacteraceae | Actinobacteria | Thermoleophilia | Solirubrobacterales |
| Soortiaceae | Balneolaeota | Balneolia | Balneolales |
| Sphaerobacteraceae | Chloroflexi | Thermomicrobia | Sphaerobacterales |
| Sphingobacteriaceae | Bacteroidetes | Sphingobacteriia | Sphingobacteriales |
| Sphingomonadaceae | Proteobacteria | Alphaproteobacteria | Sphingomonadales |
| Spirochaetaceae | Spirochaetes | Spirochaetia | Spirochaetales |
| Spiroplasmataceae | Tenericutes | Mollicutes | Entomoplasmatales |
| Spirulinaceae | Cyanobacteria |  | Spirulinales |
| Spongiibacteraceae | Proteobacteria | Gammaproteobacteria | Cellvibrionales |
| Sporichthyaceae | Actinobacteria | Actinobacteria | Sporichthyales |
| Sporolactobacillaceae | Firmicutes | Bacilli | Bacillales |
| Sporomusaceae | Firmicutes | Negativicutes | Selenomonadales |
| Steroidobacteraceae | Proteobacteria | Gammaproteobacteria | Nevskiales |
| Sterolibacteriaceae | Proteobacteria | Betaproteobacteria | Nitrosomonadales |
| Streptomycetaceae | Actinobacteria | Actinobacteria | Streptomycetales |
| Streptosporangiaceae | Actinobacteria | Actinobacteria | Streptosporangiales |
| Symbiobacteriaceae | Firmicutes | Clostridia | Eubacteriales |
| Synechococcaceae | Cyanobacteria |  | Synechococcales |
| Synergistaceae | Synergistetes | Synergistia | Synergistales |
| Syntrophaceae | Proteobacteria | Deltaproteobacteria | Syntrophobacterales |
| Syntrophobacteraceae | Proteobacteria | Deltaproteobacteria | Syntrophobacterales |
| Syntrophomonadaceae | Firmicutes | Clostridia | Eubacteriales |
| Syntrophorhabdaceae | Proteobacteria | Deltaproteobacteria | Syntrophobacterales |
| Tannerellaceae | Bacteroidetes | Bacteroidia | Bacteroidales |
| Tepidisphaeraceae | Planctomycetes | Phycisphaerae | Tepidisphaerales |
| Thermaceae | Deinococcus-Thermus | Deinococci | Thermales |
| Thermoactinomycetaceae | Firmicutes | Bacilli | Bacillales |
| Thermoanaerobacterales Family III. Incertae Sedis | Firmicutes | Clostridia | Thermoanaerobacterales |
| Thermoanaerobacterales Family IV. Incertae Sedis | Firmicutes | Clostridia | Thermoanaerobacterales |
| Thermoanaerobaculaceae | Acidobacteria | Thermoanaerobaculia | Thermoanaerobaculales |
| Thermodesulfobacteriaceae | Thermodesulfobacteria | Thermodesulfobacteria | Thermodesulfobacteriales |
| Thermogemmatisporaceae | Chloroflexi | Ktedonobacteria | Thermogemmatisporales |
| Thermoleophilaceae | Actinobacteria | Thermoleophilia | Thermoleophilales |
| Thermomicrobiaceae | Chloroflexi | Thermomicrobia | Thermomicrobiales |
| Thermomonosporaceae | Actinobacteria | Actinobacteria | Streptosporangiales |
| Thermosporotrichaceae | Chloroflexi | Ktedonobacteria | Ktedonobacterales |
| Thermotogaceae | Thermotogae | Thermotogae | Thermotogales |
| Thioalkalispiraceae | Proteobacteria | Gammaproteobacteria | Chromatiales |
| Thiobacillaceae | Proteobacteria | Betaproteobacteria | Nitrosomonadales |
| Thiotrichaceae | Proteobacteria | Gammaproteobacteria | Thiotrichales |
| Tissierellaceae | Firmicutes | Tissierellia | Tissierellales |
| Trueperaceae | Deinococcus-Thermus | Deinococci | Deinococcales |
| Tsukamurellaceae | Actinobacteria | Actinobacteria | Corynebacteriales |
| Veillonellaceae | Firmicutes | Negativicutes | Veillonellales |
| Verrucomicrobia subdivision 3 | Verrucomicrobia | Verrucomicrobiae | Verrucomicrobiales |
| Verrucomicrobiaceae | Verrucomicrobia | Verrucomicrobiae | Verrucomicrobiales |
| Vicinamibacteraceae | Acidobacteria | Acidobacteria subdivision 6 |  |
| Vulgatibacteraceae | Proteobacteria | Deltaproteobacteria | Myxococcales |
| Waddliaceae | Chlamydiae | Chlamydiia | Chlamydiales |
| Wenzhouxiangellaceae | Proteobacteria | Gammaproteobacteria | Chromatiales |
| Williamwhitmaniaceae | Bacteroidetes | Bacteroidia | Bacteroidales |
| Woeseiaceae | Proteobacteria | Gammaproteobacteria | Chromatiales |
| Xanthobacteraceae | Proteobacteria | Alphaproteobacteria | Rhizobiales |
| Xenococcaceae | Cyanobacteria |  | Pleurocapsales |
| Yersiniaceae | Proteobacteria | Gammaproteobacteria | Enterobacterales |
| Zoogloeaceae | Proteobacteria | Betaproteobacteria | Rhodocyclales |

**B.**  **The differential families calculated based on numbers of member species**

| **Family** | **Phylum** | **Class** | **Order** |
| --- | --- | --- | --- |
| Alicyclobacillaceae | Firmicutes | Bacilli | Bacillales |
| Anaerolineaceae | Chloroflexi | Anaerolineae | Anaerolineales |
| Blastocatellaceae | Acidobacteria | Blastocatellia | Blastocatellales |
| Desulfobulbaceae | Proteobacteria | Deltaproteobacteria | Desulfobacterales |
| Gemmataceae | Planctomycetes | Planctomycetia | Planctomycetales |
| Iamiaceae | Actinobacteria | Acidimicrobiia | Acidimicrobiales |
| Isosphaeraceae | Planctomycetes | Planctomycetia | Planctomycetales |
| Methylococcaceae | Proteobacteria | Gammaproteobacteria | Methylococcales |
| Micromonosporaceae | Actinobacteria | Actinobacteria | Micromonosporales |
| Nostocaceae | Cyanobacteria |  | Nostocales |
| Opitutaceae | Verrucomicrobia | Opitutae | Opitutales |
| Polyangiaceae | Proteobacteria | Deltaproteobacteria | Myxococcales |
| Pyrinomonadaceae | Acidobacteria | Blastocatellia | Blastocatellales |
| Sporomusaceae | Firmicutes | Negativicutes | Selenomonadales |
| Syntrophaceae | Proteobacteria | Deltaproteobacteria | Syntrophobacterales |
| Syntrophobacteraceae | Proteobacteria | Deltaproteobacteria | Syntrophobacterales |
| Thermoactinomycetaceae | Firmicutes | Bacilli | Bacillales |
| Verrucomicrobiaceae | Verrucomicrobia | Verrucomicrobiae | Verrucomicrobiales |

**Supplementary Table S2: The differential genera of soil bacteria in the selected ASEAN countries** (Gneiss and OLS regression, *P*-value < 1E-6, calculation based on numbers of member species)

| **Genus** | **Phylum** | **Class** | **Order** | **Family** |
| --- | --- | --- | --- | --- |
| *Acetobacter* | Proteobacteria | Alphaproteobacteria | Rhodospirillales | Acetobacteraceae |
| *Acetobacteroides* | Bacteroidetes | Bacteroidia | Bacteroidales | Rikenellaceae |
| *Acidisphaera* | Proteobacteria | Alphaproteobacteria | Rhodospirillales | Acetobacteraceae |
| *Aerosakkonema* | Cyanobacteria |  | Oscillatoriales | Oscillatoriaceae |
| *Aliinostoc* | Cyanobacteria |  | Nostocales | Nostocaceae |
| *Aminipila* | Firmicutes | Clostridia | Eubacteriales | Eubacteriales Family XIII. Incertae Sedis |
| *Anaerolinea* | Chloroflexi | Anaerolineae | Anaerolineales | Anaerolineaceae |
| *Anaerosporomusa* | Firmicutes | Negativicutes | Selenomonadales | Sporomusaceae |
| *Aquihabitans* | Actinobacteria | Acidimicrobiia | Acidimicrobiales | Iamiaceae |
| *Archangium* | Proteobacteria | Deltaproteobacteria | Myxococcales | Archangiaceae |
| *Azonexus* | Proteobacteria | Betaproteobacteria | Rhodocyclales | Azonexaceae |
| *Bacteriovorax* | Proteobacteria | Oligoflexia | Bacteriovoracales | Bacteriovoracaceae |
| *Bellilinea* | Chloroflexi | Anaerolineae | Anaerolineales | Anaerolineaceae |
| *Brevifollis* | Verrucomicrobia | Verrucomicrobiae | Verrucomicrobiales | Verrucomicrobiaceae |
| *Bythopirellula* | Planctomycetes | Planctomycetia | Pirellulales | Lacipirellulaceae |
| *Calochaete* | Cyanobacteria |  | Nostocales | Fortieaceae |
| *Carboxydocella* | Firmicutes | Clostridia | Eubacteriales | Eubacteriales Family XVI. Incertae Sedis |
| *Carboxydothermus* | Firmicutes | Clostridia | Thermoanaerobacterales | Thermoanaerobacteraceae |
| *Cellvibrio* | Proteobacteria | Gammaproteobacteria | Cellvibrionales | Cellvibrionaceae |
| *Chitinispirillum* | Fibrobacteres | Chitinispirillia | Chitinispirillales | Chitinispirillaceae |
| *Chlorogloeopsis* | Cyanobacteria |  | Nostocales | Chlorogloeopsidaceae |
| *Clostridioides* | Firmicutes | Clostridia | Eubacteriales | Peptostreptococcaceae |
| *Cronbergia* | Cyanobacteria |  | Nostocales | Nostocaceae |
| *Curvibacter* | Proteobacteria | Betaproteobacteria | Burkholderiales | Comamonadaceae |
| *Cutibacterium* | Actinobacteria | Actinobacteria | Propionibacteriales | Propionibacteriaceae |
| *Cyanobium* | Cyanobacteria |  | Synechococcales | Synechococcaceae |
| *Defluviicoccus* | Proteobacteria | Alphaproteobacteria | Rhodospirillales | Rhodospirillaceae |
| *Dehalobacter* | Firmicutes | Clostridia | Eubacteriales | Peptococcaceae |
| *Dehalogenimonas* | Chloroflexi | Dehalococcoidia |  |  |
| *Desulfitobacterium* | Firmicutes | Clostridia | Eubacteriales | Peptococcaceae |
| *Desulfobacterium* | Proteobacteria | Deltaproteobacteria | Desulfobacterales | Desulfobacteraceae |
| *Desulfomonile* | Proteobacteria | Deltaproteobacteria | Syntrophobacterales | Syntrophaceae |
| *Desulfonauticus* | Proteobacteria | Deltaproteobacteria | Desulfovibrionales | Desulfohalobiaceae |
| *Desulfonispora* | Firmicutes | Clostridia | Eubacteriales | Peptococcaceae |
| *Desulforhabdus* | Proteobacteria | Deltaproteobacteria | Syntrophobacterales | Syntrophobacteraceae |
| *Desulfosporosinus* | Firmicutes | Clostridia | Eubacteriales | Peptococcaceae |
| *Desulfovirga* | Proteobacteria | Deltaproteobacteria | Syntrophobacterales | Syntrophobacteraceae |
| *Desulfuromonas* | Proteobacteria | Deltaproteobacteria | Desulfuromonadales | Desulfuromonadaceae |
| *Dethiobacter* | Firmicutes | Clostridia | Eubacteriales | Syntrophomonadaceae |
| *Dokdonella* | Proteobacteria | Gammaproteobacteria | Lysobacterales | Rhodanobacteraceae |
| *Endomicrobium* | Elusimicrobia | Endomicrobia | Endomicrobiales | Endomicrobiaceae |
| *Enhydrobacter* | Proteobacteria | Alphaproteobacteria | Rhodospirillales |  |
| *Ereboglobus* | Verrucomicrobia | Opitutae | Opitutales | Opitutaceae |
| *Escherichia* | Proteobacteria | Gammaproteobacteria | Enterobacterales | Enterobacteriaceae |
| *Ferrovibrio* | Proteobacteria | Alphaproteobacteria | Rhodospirillales | Rhodospirillaceae |
| *Flammeovirga* | Bacteroidetes | Cytophagia | Cytophagales | Flammeovirgaceae |
| *Fonticella* | Firmicutes | Clostridia | Eubacteriales | Clostridiaceae |
| *Fusibacter* | Firmicutes | Clostridia | Eubacteriales | Eubacteriales Family XII. Incertae Sedis |
| *Gardnerella* | Actinobacteria | Actinobacteria | Bifidobacteriales | Bifidobacteriaceae |
| *Geminisphaera* | Verrucomicrobia | Opitutae | Opitutales | Opitutaceae |
| *Geothrix* | Acidobacteria | Holophagae | Holophagales | Holophagaceae |
| *Heliobacterium* | Firmicutes | Clostridia | Eubacteriales | Heliobacteriaceae |
| *Hydrogenispora* | Firmicutes |  |  |  |
| *Iamia* | Actinobacteria | Acidimicrobiia | Acidimicrobiales | Iamiaceae |
| *Ignavibacterium* | Ignavibacteriae | Ignavibacteria | Ignavibacteriales | Ignavibacteriaceae |
| *Inhella* | Proteobacteria | Betaproteobacteria | Burkholderiales |  |
| *Klebsiella* | Proteobacteria | Gammaproteobacteria | Enterobacterales | Enterobacteriaceae |
| *Kofleria* | Proteobacteria | Deltaproteobacteria | Myxococcales | Kofleriaceae |
| *Lacunisphaera* | Verrucomicrobia | Opitutae | Opitutales | Opitutaceae |
| *Leeia* | Proteobacteria | Betaproteobacteria | Neisseriales | Chromobacteriaceae |
| *Legionella* | Proteobacteria | Gammaproteobacteria | Legionellales | Legionellaceae |
| *Lentimicrobium* | Bacteroidetes | Bacteroidia | Bacteroidales | Lentimicrobiaceae |
| *Leptolinea* | Chloroflexi | Anaerolineae | Anaerolineales | Anaerolineaceae |
| *Litorilinea* | Chloroflexi | Caldilineae | Caldilineales | Caldilineaceae |
| *Longilinea* | Chloroflexi | Anaerolineae | Anaerolineales | Anaerolineaceae |
| *Longivirga* | Actinobacteria | Actinobacteria | Sporichthyales | Sporichthyaceae |
| *Luteolibacter* | Verrucomicrobia | Verrucomicrobiae | Verrucomicrobiales | Verrucomicrobiaceae |
| *Magnetospirillum* | Proteobacteria | Alphaproteobacteria | Rhodospirillales | Rhodospirillaceae |
| *Melioribacter* | Ignavibacteriae | Ignavibacteria | Ignavibacteriales | Melioribacteraceae |
| *Methylomagnum* | Proteobacteria | Gammaproteobacteria | Methylococcales | Methylococcaceae |
| *Methylosarcina* | Proteobacteria | Gammaproteobacteria | Methylococcales | Methylococcaceae |
| *Microcystis* | Cyanobacteria |  | Chroococcales | Microcystaceae |
| *Minicystis* | Proteobacteria | Deltaproteobacteria | Myxococcales |  |
| *Mycoavidus* | Proteobacteria | Betaproteobacteria | Burkholderiales | Burkholderiaceae |
| *Mycobacteroides* | Actinobacteria | Actinobacteria | Corynebacteriales | Mycobacteriaceae |
| *Nannocystis* | Proteobacteria | Deltaproteobacteria | Myxococcales | Nannocystaceae |
| *Neosynechococcus* | Cyanobacteria |  | Synechococcales | Leptolyngbyaceae |
| *Nitrosomonas* | Proteobacteria | Betaproteobacteria | Nitrosomonadales | Nitrosomonadaceae |
| *Nodularia* | Cyanobacteria |  | Nostocales | Aphanizomenonaceae |
| *Novosphingobium* | Proteobacteria | Alphaproteobacteria | Sphingomonadales | Sphingomonadaceae |
| *Oleiharenicola* | Verrucomicrobia | Opitutae | Opitutales | Opitutaceae |
| *Oscillochloris* | Chloroflexi | Chloroflexia | Chloroflexales | Oscillochloridaceae |
| *Paludibacter* | Bacteroidetes | Bacteroidia | Bacteroidales | Paludibacteraceae |
| *Pirellula* | Planctomycetes | Planctomycetia | Planctomycetales | Planctomycetaceae |
| *Planktothricoides* | Cyanobacteria |  | Oscillatoriales | Microcoleaceae |
| *Plasticicumulans* | Proteobacteria | Gammaproteobacteria |  | Candidatus Competibacteraceae |
| *Proteiniclasticum* | Firmicutes | Clostridia | Eubacteriales | Clostridiaceae |
| *Pseudoalteromonas* | Proteobacteria | Gammaproteobacteria | Alteromonadales | Pseudoalteromonadaceae |
| *Pseudoflavonifractor* | Firmicutes | Clostridia | Eubacteriales | Oscillospiraceae |
| *Rectinema* | Spirochaetes | Spirochaetia | Spirochaetales | Spirochaetaceae |
| *Rhodovastum* | Proteobacteria | Alphaproteobacteria | Rhodospirillales | Acetobacteraceae |
| *Rhodovulum* | Proteobacteria | Alphaproteobacteria | Rhodobacterales | Rhodobacteraceae |
| *Romboutsia* | Firmicutes | Clostridia | Eubacteriales | Peptostreptococcaceae |
| *Ruminiclostridium* | Firmicutes | Clostridia | Eubacteriales | Oscillospiraceae |
| *Saccharofermentans* | Firmicutes | Clostridia | Eubacteriales | Oscillospiraceae |
| *Sandaracinus* | Proteobacteria | Deltaproteobacteria | Myxococcales | Sandaracinaceae |
| *Sandarakinorhabdus* | Proteobacteria | Alphaproteobacteria | Sphingomonadales | Sphingomonadaceae |
| *Silanimonas* | Proteobacteria | Gammaproteobacteria | Lysobacterales | Lysobacteraceae |
| *Silvanigrella* | Proteobacteria | Oligoflexia | Silvanigrellales | Silvanigrellaceae |
| *Smithella* | Proteobacteria | Deltaproteobacteria | Syntrophobacterales | Syntrophaceae |
| *Sporobacter* | Firmicutes | Clostridia | Eubacteriales | Oscillospiraceae |
| *Stenotrophobacter* | Acidobacteria | Blastocatellia | Blastocatellales | Blastocatellaceae |
| *Sulfuricurvum* | Proteobacteria | Epsilonproteobacteria | Campylobacterales | Helicobacteraceae |
| *Sutterella* | Proteobacteria | Betaproteobacteria | Burkholderiales | Sutterellaceae |
| *Synechococcus* | Cyanobacteria |  | Synechococcales | Synechococcaceae |
| *Syntrophorhabdus* | Proteobacteria | Deltaproteobacteria | Syntrophobacterales | Syntrophorhabdaceae |
| *Syntrophus* | Proteobacteria | Deltaproteobacteria | Syntrophobacterales | Syntrophaceae |
| *Taylorella* | Proteobacteria | Betaproteobacteria | Burkholderiales | Alcaligenaceae |
| *Tellurimicrobium* | Acidobacteria | Blastocatellia | Blastocatellales | Blastocatellaceae |
| *Thermoanaerobaculum* | Acidobacteria | Thermoanaerobaculia | Thermoanaerobaculales | Thermoanaerobaculaceae |
| *Thermomarinilinea* | Chloroflexi | Anaerolineae | Anaerolineales | Anaerolineaceae |
| *Thermotalea* | Firmicutes | Clostridia | Eubacteriales | Clostridiaceae |
| *Vampirovibrio* | Candidatus Melainabacteria | | Bdellovibrionales | Bdellovibrionaceae |
| *Verrucosispora* | Actinobacteria | Actinobacteria | Micromonosporales | Micromonosporaceae |
| *Virgibacillus* | Firmicutes | Bacilli | Bacillales | Bacillaceae |
| *Williamwhitmania* | Bacteroidetes | Bacteroidia | Bacteroidales | Williamwhitmaniaceae |
| *Zavarzinella* | Planctomycetes | Planctomycetia | Gemmatales | Gemmataceae |

**Supplementary Table S3: The enriched bacterial species in the soil microbiomes from the selected ASEAN countries** (Pearson's Chi-squared test, adjusted *P-*value < 1e-6).

| **Species** | **Phylum** | **Class** | **Order** | **Family** | **enriched country** |
| --- | --- | --- | --- | --- | --- |
| *Acetobacteroides hydrogenigenes* | Bacteroidetes | Bacteroidia | Bacteroidales | Rikenellaceae | Thailand |
| Acidobacteria bacterium | Acidobacteria |  |  |  | Thailand |
| Acidobacteriaceae bacterium KBS 96 | Acidobacteria | Acidobacteriia | Acidobacteriales | Acidobacteriaceae | Thailand |
| Acidobacteriia bacterium | Acidobacteria | Acidobacteriia |  |  | Thailand |
| Acidobacteriia bacterium AA117 | Acidobacteria | Acidobacteriia |  |  | Thailand |
| Actinobacteria bacterium 13_2_20CM_2_66_6 | Actinobacteria |  |  |  | Thailand |
| *Aliinostoc morphoplasticum* | Cyanobacteria | Gloeobacteria | Nostocales | Nostocaceae | Thailand |
| *Anaerocolumna aminovalerica* | Firmicutes | Clostridia | Eubacteriales | Lachnospiraceae | Thailand |
| *Ancylobacter oerskovii* | Proteobacteria | Alphaproteobacteria | Rhizobiales | Xanthobacteraceae | Thailand |
| *Armatimonas rosea* | Armatimonadetes | Armatimonadia | Armatimonadales | Armatimonadaceae | Thailand |
| *Burkholderia cepacia* | Proteobacteria | Betaproteobacteria | Burkholderiales | Burkholderiaceae | Thailand |
| *Burkholderia pseudomallei* | Proteobacteria | Betaproteobacteria | Burkholderiales | Burkholderiaceae | Thailand |
| *Burkholderia pseudomultivorans* | Proteobacteria | Betaproteobacteria | Burkholderiales | Burkholderiaceae | Thailand |
| *Calochaete cimrmanii* | Cyanobacteria | Gloeobacteria | Nostocales | Fortieaceae | Thailand |
| *Candidatus Koribacter versatilis* | Acidobacteria | Acidobacteriia | Acidobacteriales | Acidobacteriaceae | Thailand |
| *Candidatus Rokubacteria bacterium* | Candidatus Rokubacteria |  |  |  | Thailand |
| *Candidatus Sulfopaludibacter* sp. SbA4 | Acidobacteria | Acidobacteriia | Bryobacterales | Solibacteraceae | Thailand |
| *Candidatus Sulfotelmatobacter kueseliae* | Acidobacteria | Acidobacteriia | Acidobacteriales | Acidobacteriaceae | Thailand |
| *Cavicella subterranea* | Proteobacteria | Gammaproteobacteria | Pseudomonadales | Moraxellaceae | Thailand |
| *Clostridium aurantibutyricum* | Firmicutes | Clostridia | Eubacteriales | Clostridiaceae | Thailand |
| *Clostridium cadaveris* | Firmicutes | Clostridia | Eubacteriales | Clostridiaceae | Thailand |
| *Clostridium herbivorans* | Firmicutes | Clostridia | Eubacteriales | Lachnospiraceae | Thailand |
| *Clostridium swellfunianum* | Firmicutes | Clostridia | Eubacteriales | Clostridiaceae | Thailand |
| *Comamonas kerstersii* | Proteobacteria | Betaproteobacteria | Burkholderiales | Comamonadaceae | Thailand |
| *Cronbergia siamensis* | Cyanobacteria | Gloeobacteria | Nostocales | Nostocaceae | Thailand |
| *Cyanobium gracile* | Cyanobacteria | Gloeobacteria | Synechococcales | Synechococcaceae | Thailand |
| *Defluviicoccus vanus* | Proteobacteria | Alphaproteobacteria | Rhodospirillales | Rhodospirillaceae | Thailand |
| *Delftia tsuruhatensis* | Proteobacteria | Betaproteobacteria | Burkholderiales | Comamonadaceae | Thailand |
| *Desulfovirga adipica* | Proteobacteria | Deltaproteobacteria | Syntrophobacterales | Syntrophobacteraceae | Thailand |
| *Desulfuromonas michiganensis* | Proteobacteria | Deltaproteobacteria | Desulfuromonadales | Desulfuromonadaceae | Thailand |
| *Elizabethkingia anophelis* | Bacteroidetes | Flavobacteriia | Flavobacteriales | Flavobacteriaceae | Thailand |
| *Endomicrobium proavitum* | Elusimicrobia | Endomicrobia | Endomicrobiales | Endomicrobiaceae | Thailand |
| *Enterobacter cancerogenus* | Proteobacteria | Gammaproteobacteria | Enterobacterales | Enterobacteriaceae | Thailand |
| *Enterococcus faecalis* | Firmicutes | Bacilli | Lactobacillales | Enterococcaceae | Thailand |
| Gemmatimonadetes bacterium | Gemmatimonadetes |  |  |  | Thailand |
| *Geobacter luticola* | Proteobacteria | Deltaproteobacteria | Desulfuromonadales | Geobacteraceae | Thailand |
| *Geobacter pelophilus* | Proteobacteria | Deltaproteobacteria | Desulfuromonadales | Geobacteraceae | Thailand |
| *Iamia majanohamensis* | Actinobacteria | Acidimicrobiia | Acidimicrobiales | Iamiaceae | Thailand |
| *Klebsiella oxytoca* | Proteobacteria | Gammaproteobacteria | Enterobacterales | Enterobacteriaceae | Thailand |
| *Kofleria flava* | Proteobacteria | Deltaproteobacteria | Myxococcales | Kofleriaceae | Thailand |
| *Lactococcus garvieae* | Firmicutes | Bacilli | Lactobacillales | Streptococcaceae | Thailand |
| *Lactococcus lactis* | Firmicutes | Bacilli | Lactobacillales | Streptococcaceae | Thailand |
| *Limnobacter litoralis* | Proteobacteria | Betaproteobacteria | Burkholderiales | Burkholderiaceae | Thailand |
| *Litorilinea aerophila* | Chloroflexi | Caldilineae | Caldilineales | Caldilineaceae | Thailand |
| *Longilinea arvoryzae* | Chloroflexi | Anaerolineae | Anaerolineales | Anaerolineaceae | Thailand |
| *Longivirga aurantiaca* | Actinobacteria | Actinobacteria | Sporichthyales | Sporichthyaceae | Thailand |
| *Microcystis aeruginosa* | Cyanobacteria |  | Chroococcales | Microcystaceae | Thailand |
| *Microvirga flocculans* | Proteobacteria | Alphaproteobacteria | Rhizobiales | Methylobacteriaceae | Thailand |
| *Minicystis rosea* | Proteobacteria | Deltaproteobacteria | Myxococcales |  | Thailand |
| *Moraxella osloensis* | Proteobacteria | Gammaproteobacteria | Pseudomonadales | Moraxellaceae | Thailand |
| *Morganella morganii* | Proteobacteria | Gammaproteobacteria | Enterobacterales | Morganellaceae | Thailand |
| *Opitutus terrae* | Verrucomicrobia | Opitutae | Opitutales | Opitutaceae | Thailand |
| *Oscillochloris trichoides* | Chloroflexi | Chloroflexia | Chloroflexales | Oscillochloridaceae | Thailand |
| *Paeniclostridium sordellii* | Firmicutes | Clostridia | Eubacteriales | Peptostreptococcaceae | Thailand |
| *Petrimonas mucosa* | Bacteroidetes | Bacteroidia | Bacteroidales | Porphyromonadaceae | Thailand |
| Planctomycetes bacterium | Planctomycetes |  |  |  | Thailand |
| *Planktothricoides raciborskii* | Cyanobacteria |  | Oscillatoriales | Microcoleaceae | Thailand |
| *Proteiniphilum saccharofermentans* | Bacteroidetes | Bacteroidia | Bacteroidales | Dysgonomonadaceae | Thailand |
| Proteobacteria bacterium | Proteobacteria |  |  |  | Thailand |
| Rhizobiales bacterium | Proteobacteria | Alphaproteobacteria | Rhizobiales |  | Thailand |
| *Ruminiclostridium cellobioparum* | Firmicutes | Clostridia | Eubacteriales | Oscillospiraceae | Thailand |
| *Sedimentibacter acidaminivorans* | Firmicutes | Tissierellia |  |  | Thailand |
| *Sedimentibacter saalensis* | Firmicutes | Tissierellia |  |  | Thailand |
| *Sorangium cellulosum* | Proteobacteria | Deltaproteobacteria | Myxococcales | Polyangiaceae | Thailand |
| *Sphingomonas agri* | Proteobacteria | Alphaproteobacteria | Sphingomonadales | Sphingomonadaceae | Thailand |
| *Thiobacillus sajanensis* | Proteobacteria | Betaproteobacteria | Nitrosomonadales | Thiobacillaceae | Thailand |
| *Thiobacillus thioparus* | Proteobacteria | Betaproteobacteria | Nitrosomonadales | Thiobacillaceae | Thailand |
| *Tissierella praeacuta* | Firmicutes | Tissierellia | Tissierellales | Tissierellaceae | Thailand |
| *Tumebacillus ginsengisoli* | Firmicutes | Bacilli | Bacillales | Alicyclobacillaceae | Thailand |
| Verrucomicrobia bacterium | Verrucomicrobia |  |  |  | Thailand |
| Verrucomicrobiaceae bacterium | Verrucomicrobia | Verrucomicrobiae | Verrucomicrobiales | Verrucomicrobiaceae | Thailand |
| *Vicinamibacter silvestris* | Acidobacteria | Vicinamibacteria |  | Vicinamibacteraceae | Thailand |
| *Acetobacter pomorum* | Proteobacteria | Alphaproteobacteria | Rhodospirillales | Acetobacteraceae | Philippines |
| Actinobacteria bacterium 66_15 | Actinobacteria | Actinobacteria |  |  | Philippines |
| *Anaerolinea thermophila* | Chloroflexi | Anaerolineae | Anaerolineales | Anaerolineaceae | Philippines |
| *Anaeromyxobacter* sp. RBG_16_69_14 | Proteobacteria | Deltaproteobacteria | Myxococcales | Anaeromyxobacteraceae | Philippines |
| bacterium P201 |  |  |  |  | Philippines |
| Bacteroidetes-Chlorobi group bacterium Naka2016 |  |  |  |  | Philippines |
| candidate division CPR2 bacterium GW2011_GWC1_41_48 | candidate division CPR2 |  |  |  | Philippines |
| candidate division CPR3 bacterium 4484_211 | candidate division CPR3 |  |  |  | Philippines |
| *Candidatus* Liptonbacteria bacterium CG11_big_fil_rev_8_21_14_0_20_35_14 | Candidatus Liptonbacteria |  |  |  | Philippines |
| *Candidatus* Zambryskibacteria bacterium CG10_big_fil_rev_8_21_14_0_10_42_12 | Candidatus Zambryskibacteria |  |  |  | Philippines |
| *Chitinispirillum alkaliphilum* | Fibrobacteres | Chitinispirillia | Chitinispirillales | Chitinispirillaceae | Philippines |
| *Chlamydia trachomatis* | Chlamydiae | Chlamydiia | Chlamydiales | Chlamydiaceae | Philippines |
| Chloroflexi bacterium | Chloroflexi |  |  |  | Philippines |
| *Clostridioides difficile* | Firmicutes | Clostridia | Eubacteriales | Peptostreptococcaceae | Philippines |
| *Clostridium bolteae* | Firmicutes | Clostridia | Eubacteriales | Lachnospiraceae | Philippines |
| *Clostridium botulinum* | Firmicutes | Clostridia | Eubacteriales | Clostridiaceae | Philippines |
| *Clostridium cellulosi* | Firmicutes | Clostridia | Eubacteriales | Oscillospiraceae | Philippines |
| *Clostridium leptum* | Firmicutes | Clostridia | Eubacteriales | Oscillospiraceae | Philippines |
| *Clostridium* sp. BL8 | Firmicutes | Clostridia | Eubacteriales | Clostridiaceae | Philippines |
| *Desulfohalovibrio alkalitolerans* | Proteobacteria | Deltaproteobacteria | Desulfovibrionales | Desulfovibrionaceae | Philippines |
| *Desulfomonile tiedjei* | Proteobacteria | Deltaproteobacteria | Syntrophobacterales | Syntrophaceae | Philippines |
| *Desulfonauticus s*p. 38_4375 | Proteobacteria | Deltaproteobacteria | Desulfovibrionales | Desulfohalobiaceae | Philippines |
| *Desulforhabdus amnigena* | Proteobacteria | Deltaproteobacteria | Syntrophobacterales | Syntrophobacteraceae | Philippines |
| *Desulfovibrio putealis* | Proteobacteria | Deltaproteobacteria | Desulfovibrionales | Desulfovibrionaceae | Philippines |
| *Dorea longicatena* | Firmicutes | Clostridia | Eubacteriales | Lachnospiraceae | Philippines |
| *Flammeovirga yaeyamensis* | Bacteroidetes | Cytophagia | Cytophagales | Flammeovirgaceae | Philippines |
| *Fonticella tunisiensis* | Firmicutes | Clostridia | Eubacteriales | Clostridiaceae | Philippines |
| *Gardnerella vaginalis* | Actinobacteria | Actinobacteria | Bifidobacteriales | Bifidobacteriaceae | Philippines |
| *Geminisphaera colitermitum* | Verrucomicrobia | Opitutae | Opitutales | Opitutaceae | Philippines |
| *Heliobacterium modesticaldum* | Firmicutes | Clostridia | Eubacteriales | Heliobacteriaceae | Philippines |
| *Klebsiella pneumoniae* | Proteobacteria | Gammaproteobacteria | Enterobacterales | Enterobacteriaceae | Philippines |
| *Lentimicrobium saccharophilum* | Bacteroidetes | Bacteroidia | Bacteroidales | Lentimicrobiaceae | Philippines |
| *Listeria monocytogenes* | Firmicutes | Bacilli | Bacillales | Listeriaceae | Philippines |
| *Methylocaldum gracile* | Proteobacteria | Gammaproteobacteria | Methylococcales | Methylococcaceae | Philippines |
| *Mycobacterium ahvazicum* | Actinobacteria | Actinobacteria | Corynebacteriales | Mycobacteriaceae | Philippines |
| *Pantoea ananatis* | Proteobacteria | Gammaproteobacteria | Enterobacterales | Erwiniaceae | Philippines |
| *Pararhodospirillum photometricum* | Proteobacteria | Alphaproteobacteria | Rhodospirillales | Rhodospirillaceae | Philippines |
| *Parvimonas micra* | Firmicutes | Tissierellia | Tissierellales | Peptoniphilaceae | Philippines |
| *Pseudoclostridium thermosuccinogenes* | Firmicutes | Clostridia | Eubacteriales | Oscillospiraceae | Philippines |
| *Pseudoflavonifractor capillosus* | Firmicutes | Clostridia | Eubacteriales | Oscillospiraceae | Philippines |
| *Rickettsia prowazekii* | Proteobacteria | Alphaproteobacteria | Rickettsiales | Rickettsiaceae | Philippines |
| *Ruminococcus gnavus* | Firmicutes | Clostridia | Eubacteriales | Lachnospiraceae | Philippines |
| *Shewanella benthica* | Proteobacteria | Gammaproteobacteria | Alteromonadales | Shewanellaceae | Philippines |
| *Sporomusa malonica* | Firmicutes | Negativicutes | Selenomonadales | Sporomusaceae | Philippines |
| *Staphylococcus aureus* | Firmicutes | Bacilli | Bacillales | Staphylococcaceae | Philippines |
| *Streptococcus pneumoniae* | Firmicutes | Bacilli | Lactobacillales | Streptococcaceae | Philippines |
| *Streptococcus ruminantium* | Firmicutes | Bacilli | Lactobacillales | Streptococcaceae | Philippines |
| *Streptococcus thermophilus* | Firmicutes | Bacilli | Lactobacillales | Streptococcaceae | Philippines |
| *Sutterella* sp. CAG:521 | Proteobacteria | Betaproteobacteria | Burkholderiales | Sutterellaceae | Philippines |
| *Synechococcus* sp. WH 8109 | Cyanobacteria | Gloeobacteria | Synechococcales | Synechococcaceae | Philippines |
| *Syntrophobacter* sp. SbD1 | Proteobacteria | Deltaproteobacteria | Syntrophobacterales | Syntrophobacteraceae | Philippines |
| *Syntrophobacter wolinii* | Proteobacteria | Deltaproteobacteria | Syntrophobacterales | Syntrophobacteraceae | Philippines |
| *Syntrophus aciditrophicus* | Proteobacteria | Deltaproteobacteria | Syntrophobacterales | Syntrophaceae | Philippines |
| *Thermotalea metallivorans* | Firmicutes | Clostridia | Eubacteriales | Clostridiaceae | Philippines |
| uncultured bacterium |  |  |  |  | Philippines |
| uncultured bacterium 5G4 |  |  |  |  | Philippines |
| uncultured *Blautia* sp. | Firmicutes | Clostridia | Eubacteriales | Lachnospiraceae | Philippines |
| uncultured *Desulfobacterium* sp. | Proteobacteria | Deltaproteobacteria | Desulfobacterales | Desulfobacteraceae | Philippines |
| uncultured sulfate-reducing bacterium |  |  |  |  | Philippines |
| uncultured *Syntrophobacter* sp. | Proteobacteria | Deltaproteobacteria | Syntrophobacterales | Syntrophobacteraceae | Philippines |
| *Vampirococcus* sp. LiM |  |  |  |  | Philippines |
| *Vibrio parahaemolyticus* | Proteobacteria | Gammaproteobacteria | Vibrionales | Vibrionaceae | Philippines |
| *Acidibacter ferrireducens* | Proteobacteria | Gammaproteobacteria |  |  | Malaysia |
| *Acidicapsa acidisoli* | Acidobacteria | Acidobacteriia | Acidobacteriales | Acidobacteriaceae | Malaysia |
| *Acidicapsa ligni* | Acidobacteria | Acidobacteriia | Acidobacteriales | Acidobacteriaceae | Malaysia |
| *Acidisoma tundrae* | Proteobacteria | Alphaproteobacteria | Rhodospirillales | Acetobacteraceae | Malaysia |
| *Aciditerrimonas ferrireducens* | Actinobacteria | Acidimicrobiia | Acidimicrobiales | Acidimicrobiaceae | Malaysia |
| *Acidobacterium ailaaui* | Acidobacteria | Acidobacteriia | Acidobacteriales | Acidobacteriaceae | Malaysia |
| *Actinocrinis puniceicyclus* | Actinobacteria | Actinobacteria | Catenulisporales | Actinospicaceae | Malaysia |
| *Adhaeribacter terrae* | Bacteroidetes | Cytophagia | Cytophagales | Hymenobacteraceae | Malaysia |
| *Adhaeribacter terreus* | Bacteroidetes | Cytophagia | Cytophagales | Hymenobacteraceae | Malaysia |
| *Alkanindiges illinoisensis* | Proteobacteria | Gammaproteobacteria | Pseudomonadales | Moraxellaceae | Malaysia |
| *Aquicella siphonis* | Proteobacteria | Gammaproteobacteria | Legionellales | Coxiellaceae | Malaysia |
| *Aquihabitans daechungensis* | Actinobacteria | Acidimicrobiia | Acidimicrobiales | Iamiaceae | Malaysia |
| *Aquisphaera giovannonii* | Planctomycetes | Planctomycetia | Planctomycetales | Isosphaeraceae | Malaysia |
| *Arenimonas subflava* | Proteobacteria | Gammaproteobacteria | Lysobacterales | Lysobacteraceae | Malaysia |
| *Bacillus thuringiensis* | Firmicutes | Bacilli | Bacillales | Bacillaceae | Malaysia |
| *Bauldia consociata* | Proteobacteria | Alphaproteobacteria | Rhizobiales |  | Malaysia |
| *Bradyrhizobium erythrophlei* | Proteobacteria | Alphaproteobacteria | Rhizobiales | Bradyrhizobiaceae | Malaysia |
| *Brevibacillus fluminis* | Firmicutes | Bacilli | Bacillales | Paenibacillaceae | Malaysia |
| *Brevibacillus ginsengisoli* | Firmicutes | Bacilli | Bacillales | Paenibacillaceae | Malaysia |
| *Burkholderia alpina* | Proteobacteria | Betaproteobacteria | Burkholderiales | Burkholderiaceae | Malaysia |
| *Bythopirellula goksoyri* | Planctomycetes | Planctomycetia | Pirellulales | Lacipirellulaceae | Malaysia |
| *Caulobacter fusiformis* | Proteobacteria | Alphaproteobacteria | Caulobacterales | Caulobacteraceae | Malaysia |
| *Caulobacter profundus* | Proteobacteria | Alphaproteobacteria | Caulobacterales | Caulobacteraceae | Malaysia |
| *Chromobacterium* sp. ATCC 53434 | Proteobacteria | Betaproteobacteria | Neisseriales | Chromobacteriaceae | Malaysia |
| *Chthoniobacter flavus* | Verrucomicrobia | Spartobacteria | Chthoniobacterales | Chthoniobacteraceae | Malaysia |
| *Dictyobacter aurantiacus* | Chloroflexi | Ktedonobacteria | Ktedonobacterales | Dictyobacteraceae | Malaysia |
| *Dinghuibacter silviterrae* | Bacteroidetes | Chitinophagia | Chitinophagales | Chitinophagaceae | Malaysia |
| *Diplorickettsia massiliensis* | Proteobacteria | Gammaproteobacteria | Legionellales | Coxiellaceae | Malaysia |
| *Duganella ginsengisoli* | Proteobacteria | Betaproteobacteria | Burkholderiales | Oxalobacteraceae | Malaysia |
| *Dyella flava* | Proteobacteria | Gammaproteobacteria | Lysobacterales | Rhodanobacteraceae | Malaysia |
| *Dyella japonica* | Proteobacteria | Gammaproteobacteria | Lysobacterales | Rhodanobacteraceae | Malaysia |
| *Dyella kyungheensis* | Proteobacteria | Gammaproteobacteria | Lysobacterales | Rhodanobacteraceae | Malaysia |
| *Dyella marensis* | Proteobacteria | Gammaproteobacteria | Lysobacterales | Rhodanobacteraceae | Malaysia |
| *Edaphobacter acidisoli* | Acidobacteria | Acidobacteriia | Acidobacteriales | Acidobacteriaceae | Malaysia |
| *Edaphobacter lichenicola* | Acidobacteria | Acidobacteriia | Acidobacteriales | Acidobacteriaceae | Malaysia |
| *Edaphobaculum flavum* | Bacteroidetes | Chitinophagia | Chitinophagales | Chitinophagaceae | Malaysia |
| *Erythrobacter luteus* | Proteobacteria | Alphaproteobacteria | Sphingomonadales | Erythrobacteraceae | Malaysia |
| *Fimbriimonas ginsengisoli* | Armatimonadetes | Fimbriimonadia | Fimbriimonadales | Fimbriimonadaceae | Malaysia |
| *Flavisolibacter tropicus* | Bacteroidetes | Chitinophagia | Chitinophagales | Chitinophagaceae | Malaysia |
| *Flavitalea flava* | Bacteroidetes | Chitinophagia | Chitinophagales | Chitinophagaceae | Malaysia |
| *Flavobacterium anhuiense* | Bacteroidetes | Flavobacteriia | Flavobacteriales | Flavobacteriaceae | Malaysia |
| *Gaiella occulta* | Actinobacteria | Rubrobacteria | Gaiellales | Gaiellaceae | Malaysia |
| *Gemmata massiliana* | Planctomycetes | Planctomycetia | Gemmatales | Gemmataceae | Malaysia |
| *Gemmatimonas phototrophica* | Gemmatimonadetes | Gemmatimonadetes | Gemmatimonadales | Gemmatimonadaceae | Malaysia |
| *Granulicella sapmiensis* | Acidobacteria | Acidobacteriia | Acidobacteriales | Acidobacteriaceae | Malaysia |
| *Haliangium ochraceum* | Proteobacteria | Deltaproteobacteria | Myxococcales | Kofleriaceae | Malaysia |
| *Ktedonobacter racemifer* | Chloroflexi | Ktedonobacteria | Ktedonobacterales | Ktedonobacteraceae | Malaysia |
| *Limisphaera ngatamarikiensis* | Verrucomicrobia | Verrucomicrobiae | Verrucomicrobiales | Verrucomicrobia subdivision 3 | Malaysia |
| *Luteitalea pratensis* | Acidobacteria | Acidobacteria subdivision 6 |  | Vicinamibacteraceae | Malaysia |
| *Luteolibacter gellanilyticus* | Verrucomicrobia | Verrucomicrobiae | Verrucomicrobiales | Verrucomicrobiaceae | Malaysia |
| *Lysobacter caseinilyticus* | Proteobacteria | Gammaproteobacteria | Lysobacterales | Lysobacteraceae | Malaysia |
| *Microlunatus endophyticus* | Actinobacteria | Actinobacteria | Propionibacteriales | Propionibacteriaceae | Malaysia |
| *Mucibacter soli* | Bacteroidetes | Chitinophagia | Chitinophagales | Chitinophagaceae | Malaysia |
| *Mycobacterium conspicuum* | Actinobacteria | Actinobacteria | Corynebacteriales | Mycobacteriaceae | Malaysia |
| *Neochlamydia hartmannellae* | Chlamydiae | Chlamydiia | Chlamydiales | Parachlamydiaceae | Malaysia |
| *Nevskia terrae* | Proteobacteria | Gammaproteobacteria | Nevskiales | Nevskiaceae | Malaysia |
| *Nordella oligomobilis* | Proteobacteria | Alphaproteobacteria | Rhizobiales |  | Malaysia |
| *Novosphingobium rosa* | Proteobacteria | Alphaproteobacteria | Sphingomonadales | Sphingomonadaceae | Malaysia |
| *Paenibacillus aceris* | Firmicutes | Bacilli | Bacillales | Paenibacillaceae | Malaysia |
| *Paenibacillus doosanensis* | Firmicutes | Bacilli | Bacillales | Paenibacillaceae | Malaysia |
| *Paenibacillus herberti* | Firmicutes | Bacilli | Bacillales | Paenibacillaceae | Malaysia |
| *Paludibaculum fermentans* | Acidobacteria | Acidobacteriia | Bryobacterales | Bryobacteraceae | Malaysia |
| *Paludisphaera borealis* | Planctomycetes | Planctomycetia | Planctomycetales | Isosphaeraceae | Malaysia |
| *Paraburkholderia nodosa* | Proteobacteria | Betaproteobacteria | Burkholderiales | Burkholderiaceae | Malaysia |
| *Paraburkholderia susongensis* | Proteobacteria | Betaproteobacteria | Burkholderiales | Burkholderiaceae | Malaysia |
| *Paracoccus denitrificans* | Proteobacteria | Alphaproteobacteria | Rhodobacterales | Rhodobacteraceae | Malaysia |
| *Paracoccus yeei* | Proteobacteria | Alphaproteobacteria | Rhodobacterales | Rhodobacteraceae | Malaysia |
| *Parafilimonas terrae* | Bacteroidetes | Chitinophagia | Chitinophagales | Chitinophagaceae | Malaysia |
| *Pedomicrobium australicum* | Proteobacteria | Alphaproteobacteria | Rhizobiales | Hyphomicrobiaceae | Malaysia |
| *Peredibacter starrii* | Proteobacteria | Oligoflexia | Bacteriovoracales | Bacteriovoracaceae | Malaysia |
| *Phenylobacterium deserti* | Proteobacteria | Alphaproteobacteria | Caulobacterales | Caulobacteraceae | Malaysia |
| *Phenylobacterium hankyongense* | Proteobacteria | Alphaproteobacteria | Caulobacterales | Caulobacteraceae | Malaysia |
| *Pirellula staleyi* | Planctomycetes | Planctomycetia | Pirellulales | Planctomycetaceae | Malaysia |
| *Pseudoduganella eburnea* | Proteobacteria | Betaproteobacteria | Burkholderiales | Oxalobacteraceae | Malaysia |
| *Pseudomonas flexibilis* | Proteobacteria | Gammaproteobacteria | Pseudomonadales | Pseudomonadaceae | Malaysia |
| *Pseudomonas lini* | Proteobacteria | Gammaproteobacteria | Pseudomonadales | Pseudomonadaceae | Malaysia |
| *Puia dinghuensis* | Bacteroidetes | Chitinophagia | Chitinophagales | Chitinophagaceae | Malaysia |
| *Reyranella soli* | Proteobacteria | Alphaproteobacteria | Rhodospirillales |  | Malaysia |
| *Rhizomicrobium electricum* | Proteobacteria | Alphaproteobacteria | Micropepsales | Micropepsaceae | Malaysia |
| *Rhodobacter* sp. LPB0142 | Proteobacteria | Alphaproteobacteria | Rhodobacterales | Rhodobacteraceae | Malaysia |
| *Rhodopila globiformis* | Proteobacteria | Alphaproteobacteria | Rhodospirillales | Acetobacteraceae | Malaysia |
| *Roseiarcus fermentans* | Proteobacteria | Alphaproteobacteria | Rhizobiales | Roseiarcaceae | Malaysia |
| *Rudaea cellulosilytica* | Proteobacteria | Gammaproteobacteria | Lysobacterales | Rhodanobacteraceae | Malaysia |
| *Silvibacterium bohemicum* | Acidobacteria | Acidobacteriia | Acidobacteriales | Acidobacteriaceae | Malaysia |
| *Singulisphaera acidiphila* | Planctomycetes | Planctomycetia | Planctomycetales | Isosphaeraceae | Malaysia |
| *Singulisphaera rosea* | Planctomycetes | Planctomycetia | Planctomycetales | Isosphaeraceae | Malaysia |
| *Sinosporangium album* | Actinobacteria | Actinobacteria | Streptosporangiales | Streptosporangiaceae | Malaysia |
| *Solitalea koreensis* | Bacteroidetes | Sphingobacteriia | Sphingobacteriales | Sphingobacteriaceae | Malaysia |
| *Sphingomonas daechungensis* | Proteobacteria | Alphaproteobacteria | Sphingomonadales | Sphingomonadaceae | Malaysia |
| *Sphingomonas oryziterrae* | Proteobacteria | Alphaproteobacteria | Sphingomonadales | Sphingomonadaceae | Malaysia |
| *Sphingomonas palustris* | Proteobacteria | Alphaproteobacteria | Sphingomonadales | Sphingomonadaceae | Malaysia |
| *Sphingorhabdus contaminans* | Proteobacteria | Alphaproteobacteria | Sphingomonadales | Sphingomonadaceae | Malaysia |
| *Sporosarcina thermotolerans* | Firmicutes | Bacilli | Bacillales | Caryophanaceae | Malaysia |
| *Stenotrophobacter terrae* | Acidobacteria | Blastocatellia | Blastocatellales | Blastocatellaceae | Malaysia |
| *Stenotrophomonas maltophilia* | Proteobacteria | Gammaproteobacteria | Lysobacterales | Lysobacteraceae | Malaysia |
| *Tellurimicrobium multivorans* | Acidobacteria | Blastocatellia | Blastocatellales | Blastocatellaceae | Malaysia |
| *Telmatospirillum siberiense* | Proteobacteria | Alphaproteobacteria | Rhodospirillales | Rhodospirillaceae | Malaysia |
| *Tepidisphaera mucosa* | Planctomycetes | Phycisphaerae | Tepidisphaerales | Tepidisphaeraceae | Malaysia |
| *Terrimicrobium sacchariphilum* | Verrucomicrobia | Spartobacteria |  |  | Malaysia |
| *Tundrisphaera lichenicola* | Planctomycetes | Planctomycetia | Planctomycetales | Isosphaeraceae | Malaysia |
| *Vulgatibacter incomptus* | Proteobacteria | Deltaproteobacteria | Myxococcales | Vulgatibacteraceae | Malaysia |
| *Yangia pacifica* | Proteobacteria | Alphaproteobacteria | Rhodobacterales | Rhodobacteraceae | Malaysia |
| *Zavarzinella formosa* | Planctomycetes | Planctomycetia | Gemmatales | Gemmataceae | Malaysia |
| *Acidisphaera rubrifaciens* | Proteobacteria | Alphaproteobacteria | Rhodospirillales | Acetobacteraceae | Indonesia |
| *Acinetobacter baumannii* | Proteobacteria | Gammaproteobacteria | Pseudomonadales | Moraxellaceae | Indonesia |
| *Aggregicoccus edonensis* | Proteobacteria | Deltaproteobacteria | Myxococcales | Myxococcaceae | Indonesia |
| *Anaeromyxobacter dehalogenans* | Proteobacteria | Deltaproteobacteria | Myxococcales | Anaeromyxobacteraceae | Indonesia |
| *Azospirillum* sp. CAG:260 | Proteobacteria | Alphaproteobacteria | Rhodospirillales | Rhodospirillaceae | Indonesia |
| *Bacillus cereus* | Firmicutes | Bacilli | Bacillales | Bacillaceae | Indonesia |
| *Bathymodiolus azoricus thioautotrophic gill symbiont* | Proteobacteria | Gammaproteobacteria |  |  | Indonesia |
| *Brucella suis* | Proteobacteria | Alphaproteobacteria | Rhizobiales | Brucellaceae | Indonesia |
| *Burkholderia cenocepacia* | Proteobacteria | Betaproteobacteria | Burkholderiales | Burkholderiaceae | Indonesia |
| *Candidatus* Giovannonibacteria bacterium RIFCSPHIGHO2_12_FULL_44_42 | Candidatus Giovannonibacteria |  |  |  | Indonesia |
| *Candidatus* Levybacteria bacterium GW2011_GWB1_41_21 | Candidatus Levybacteria |  |  |  | Indonesia |
| *Candidatus Solibacter usitatus* | Acidobacteria | Acidobacteriia | Bryobacterales | Solibacteraceae | Indonesia |
| *Candidatus* Woesebacteria bacterium RBG_16_36_11 | Candidatus Woesebacteria |  |  |  | Indonesia |
| *Carboxydocella* sp. JDF658 | Firmicutes | Clostridia | Eubacteriales | Eubacteriales Family XVI. Incertae Sedis | Indonesia |
| *Chujaibacter soli* | Proteobacteria | Gammaproteobacteria | Lysobacterales | Lysobacteraceae | Indonesia |
| *Comamonas testosteroni* | Proteobacteria | Betaproteobacteria | Burkholderiales | Comamonadaceae | Indonesia |
| *Crenothrix polyspora* | Proteobacteria | Gammaproteobacteria | Methylococcales | Crenotrichaceae | Indonesia |
| Curvibacter putative symbiont of Hydra magnipapillata | Proteobacteria | Betaproteobacteria | Burkholderiales | Comamonadaceae | Indonesia |
| *Cutibacterium acnes* | Actinobacteria | Actinobacteria | Propionibacteriales | Propionibacteriaceae | Indonesia |
| *Dethiobacter alkaliphilus* | Firmicutes | Clostridia | Eubacteriales | Syntrophomonadaceae | Indonesia |
| *Escherichia coli* | Proteobacteria | Gammaproteobacteria | Enterobacterales | Enterobacteriaceae | Indonesia |
| *Legionella pneumophila* | Proteobacteria | Gammaproteobacteria | Legionellales | Legionellaceae | Indonesia |
| *Listeria seeligeri* | Firmicutes | Bacilli | Bacillales | Listeriaceae | Indonesia |
| *Magnetospirillum* sp. XM-1 | Proteobacteria | Alphaproteobacteria | Rhodospirillales | Rhodospirillaceae | Indonesia |
| methanotrophic bacterial endosymbiont of *Bathymodiolus* sp. | Proteobacteria | Gammaproteobacteria |  |  | Indonesia |
| *Mycobacterium tuberculosis* | Actinobacteria | Actinobacteria | Corynebacteriales | Mycobacteriaceae | Indonesia |
| *Mycobacteroides abscessus* | Actinobacteria | Actinobacteria | Corynebacteriales | Mycobacteriaceae | Indonesia |
| *Nitrospira japonica* | Nitrospirae | Nitrospira | Nitrospirales | Nitrospiraceae | Indonesia |
| *Novimethylophilus kurashikiensis* | Proteobacteria | Betaproteobacteria | Nitrosomonadales | Methylophilaceae | Indonesia |
| *Paenarthrobacter nicotinovorans* | Actinobacteria | Actinobacteria | Micrococcales | Micrococcaceae | Indonesia |
| *Povalibacter uvarum* | Proteobacteria | Gammaproteobacteria | Nevskiales | Steroidobacteraceae | Indonesia |
| *Rhodovulum sulfidophilum* | Proteobacteria | Alphaproteobacteria | Rhodobacterales | Rhodobacteraceae | Indonesia |
| *Rugosibacter aromaticivorans* | Proteobacteria | Betaproteobacteria | Rhodocyclales | Rhodocyclaceae | Indonesia |
| *Salmonella enterica* | Proteobacteria | Gammaproteobacteria | Enterobacterales | Enterobacteriaceae | Indonesia |
| *Streptomyces cinnamoneus* | Actinobacteria | Actinobacteria | Streptomycetales | Streptomycetaceae | Indonesia |
| *Streptomyces griseocarneus* | Actinobacteria | Actinobacteria | Streptomycetales | Streptomycetaceae | Indonesia |
| *Streptomyces himastatinicus* | Actinobacteria | Actinobacteria | Streptomycetales | Streptomycetaceae | Indonesia |
| *Taylorella asinigenitalis* | Proteobacteria | Betaproteobacteria | Burkholderiales | Alcaligenaceae | Indonesia |
| *Thermodesulfobacterium commune* | Thermodesulfobacteria | Thermodesulfobacteria | Thermodesulfobacteriales | Thermodesulfobacteriaceae | Indonesia |
| *Thermus thermophilus* | Deinococcus-Thermus | Deinococci | Thermales | Thermaceae | Indonesia |
| uncultured bacterium 5G12 | Actinobacteria | Actinobacteria | Gaiellales | Rubrobacteridae | Indonesia |
| uncultured bacterium FPPP_33K14 |  |  |  |  | Indonesia |
| uncultured *Pleomorphomonas* sp. | Proteobacteria | Alphaproteobacteria | Rhizobiales | Methylocystaceae | Indonesia |
| *Virgibacillus profundi* | Firmicutes | Bacilli | Bacillales | Bacillaceae | Indonesia |
| *Xanthomonas citri* | Proteobacteria | Gammaproteobacteria | Lysobacterales | Lysobacteraceae | Indonesia |

**Supplementary Table S4: The community membership of the association networks of top abundant bacterial species in Thailand’s soil microbiomes**

| **Community** | **Species** | **Node degree** | **Betweenness** | **Phylum** | **Class** | **Order** | **Family** |
| --- | --- | --- | --- | --- | --- | --- | --- |
| T1 | *Clostridium swellfunianum* | 6 | 103 | Firmicutes | Clostridia | Eubacteriales | Clostridiaceae |
|  | *Gaiella occulta* | 2 | 18 | Actinobacteria | Rubrobacteria | Gaiellales | Gaiellaceae |
|  | *Clostridium aurantibutyricum* | 2 | 18 | Firmicutes | Clostridia | Eubacteriales | Clostridiaceae |
|  | *Aliinostoc morphoplasticum* | 1 | 0 | Cyanobacteria | Gloeobacteria | Nostocales | Nostocaceae |
|  | *Thiobacillus sajanensis* | 1 | 0 | Proteobacteria | Betaproteobacteria | Nitrosomonadales | Thiobacillaceae |
|  | *Cronbergia siamensis* | 1 | 0 | Cyanobacteria | Gloeobacteria | Nostocales | Nostocaceae |
|  | *Chitinispirillum alkaliphilum* | 1 | 0 | Fibrobacteres | Chitinispirillia | Chitinispirillales | Chitinispirillaceae |
|  | *Gemmata massiliana* | 1 | 0 | Planctomycetes | Planctomycetia | Gemmatales | Gemmataceae |
| T2 | *Tumebacillus ginsengisoli* | 5 | 125 | Firmicutes | Bacilli | Bacillales | Alicyclobacillaceae |
|  | *Aquihabitans daechungensis* | 3 | 35 | Actinobacteria | Acidimicrobiia | Acidimicrobiales | Iamiaceae |
|  | *Armatimonas rosea* | 1 | 0 | Armatimonadetes | Armatimonadia | Armatimonadales | Armatimonadaceae |
|  | *Oscillochloris trichoides* | 1 | 0 | Chloroflexi | Chloroflexia | Chloroflexales | Oscillochloridaceae |
|  | *Calochaete cimrmanii* | 1 | 0 | Cyanobacteria | Gloeobacteria | Nostocales | Fortieaceae |
|  | *Acetobacteroides hydrogenigenes* | 1 | 0 | Bacteroidetes | Bacteroidia | Bacteroidales | Rikenellaceae |
| T3 | *Aquisphaera giovannonii* | 5 | 79 | Planctomycetes | Planctomycetia | Planctomycetales | Isosphaeraceae |
|  | *Acidibacter ferrireducens* | 2 | 18 | Proteobacteria | Gammaproteobacteria |  |  |
|  | *Opitutus terrae* | 1 | 0 | Verrucomicrobia | Opitutae | Opitutales | Opitutaceae |
|  | *Tepidisphaera mucosa* | 1 | 0 | Planctomycetes | Phycisphaerae | Tepidisphaerales | Tepidisphaeraceae |
|  | *Thiobacillus thioparus* | 1 | 0 | Proteobacteria | Betaproteobacteria | Nitrosomonadales | Thiobacillaceae |
|  | *Paludibaculum fermentans* | 1 | 0 | Acidobacteria | Acidobacteriia | Bryobacterales | Bryobacteraceae |

**Supplementary Table S5: The community membership of the association networks of top abundant bacterial species in Malaysia’s soil microbiomes**

| **Community** | **species** | **Node degree** | **Betweenness** | **Phylum** | **Class** | **Order** | **Family** |
| --- | --- | --- | --- | --- | --- | --- | --- |
| M1 | *Chthoniobacter flavus* | 6 | 104 | Verrucomicrobia | Spartobacteria | Chthoniobacterales | Chthoniobacteraceae |
|  | *Roseiarcus fermentans* | 2 | 16 | Proteobacteria | Alphaproteobacteria | Rhizobiales | Roseiarcaceae |
|  | *Aquihabitans daechungensis* | 1 | 0 | Actinobacteria | Acidimicrobiia | Acidimicrobiales | Iamiaceae |
|  | *Dinghuibacter silviterrae* | 1 | 0 | Bacteroidetes | Chitinophagia | Chitinophagales | Chitinophagaceae |
|  | *Nevskia terrae* | 1 | 0 | Proteobacteria | Gammaproteobacteria | Nevskiales | Nevskiaceae |
|  | *Actinocrinis puniceicyclus* | 1 | 0 | Actinobacteria | Actinobacteria | Catenulisporales | Actinospicaceae |
| M2 | *Mucibacter soli* | 4 | 45 | Bacteroidetes | Chitinophagia | Chitinophagales | Chitinophagaceae |
|  | *Dictyobacter aurantiacus* | 2 | 52 | Chloroflexi | Ktedonobacteria | Ktedonobacterales | Dictyobacteraceae |
|  | *Gemmata massiliana* | 1 | 0 | Planctomycetes | Planctomycetia | Gemmatales | Gemmataceae |
|  | *Zavarzinella formosa* | 1 | 0 | Planctomycetes | Planctomycetia | Gemmatales | Gemmataceae |
|  | *Aquisphaera giovannonii* | 1 | 0 | Planctomycetes | Planctomycetia | Planctomycetales | Isosphaeraceae |
| M3 | *Tepidisphaera mucosa* | 5 | 78 | Planctomycetes | Phycisphaerae | Tepidisphaerales | Tepidisphaeraceae |
|  | *Paludibaculum fermentans* | 1 | 0 | Acidobacteria | Acidobacteriia | Bryobacterales | Bryobacteraceae |
|  | *Puia dinghuensis* | 1 | 0 | Bacteroidetes | Chitinophagia | Chitinophagales | Chitinophagaceae |
|  | *Nitrospira japonica* | 1 | 0 | Nitrospirae | Nitrospira | Nitrospirales | Nitrospiraceae |
| M4 | *Gaiella occulta* | 3 | 31 | Actinobacteria | Rubrobacteria | Gaiellales | Gaiellaceae |
|  | *Acidibacter ferrireducens* | 1 | 0 | Proteobacteria | Gammaproteobacteria |  |  |
|  | *Limisphaera ngatamarikiensis* | 1 | 0 | Verrucomicrobia | Verrucomicrobiae | Verrucomicrobiales | Verrucomicrobia subdivision 3 |

**Supplementary Table S6: The community membership of the association networks of top abundant bacterial species in the Philippines’ soil microbiomes**

| **Community** | **species** | **Node degree** | **Betweenness** | **Phylum** | **Class** | **Order** | **Family** |
| --- | --- | --- | --- | --- | --- | --- | --- |
| P1 | *Klebsiella pneumoniae* | 2 | 9 | Proteobacteria | Gammaproteobacteria | Enterobacterales | Enterobacteriaceae |
|  | *Streptomyces himastatinicus* | 2 | 16 | Actinobacteria | Actinobacteria | Streptomycetales | Streptomycetaceae |
|  | *Synechococcus* sp. | 2 | 21 | Cyanobacteria | Gloeobacteria | Synechococcales | Synechococcaceae |
|  | *Clostridium botulinum* | 2 | 24 | Firmicutes | Clostridia | Eubacteriales | Clostridiaceae |
|  | *Acetobacter pomorum* | 2 | 25 | Proteobacteria | Alphaproteobacteria | Rhodospirillales | Acetobacteraceae |
|  | *Streptomyces griseocarneus* | 1 | 0 | Actinobacteria | Actinobacteria | Streptomycetales | Streptomycetaceae |
| P2 | *Paludibaculum fermentans* | 5 | 94 | Acidobacteria | Acidobacteriia | Bryobacterales | Bryobacteraceae |
|  | *Clostridium aurantibutyricum* | 3 | 33 | Firmicutes | Clostridia | Eubacteriales | Clostridiaceae |
|  | *Zavarzinella formosa* | 2 | 17 | Planctomycetes | Planctomycetia | Gemmatales | Gemmataceae |
|  | *Tepidisphaera mucosa* | 1 | 0 | Planctomycetes | Phycisphaerae | Tepidisphaerales | Tepidisphaeraceae |
|  | *Reyranella soli* | 1 | 0 | Proteobacteria | Alphaproteobacteria | Rhodospirillales |  |
|  | *Minicystis rosea* | 1 | 0 | Proteobacteria | Deltaproteobacteria | Myxococcales |  |
|  | *Acidibacter ferrireducens* | 1 | 0 | Proteobacteria | Gammaproteobacteria |  |  |
|  | *Nitrospira japonica* | 1 | 0 | Nitrospirae | Nitrospira | Nitrospirales | Nitrospiraceae |
| P3 | *Armatimonas rosea* | 3 | 33 | Armatimonadetes | Armatimonadia | Armatimonadales | Armatimonadaceae |
|  | *Aquihabitans daechungensis* | 1 | 0 | Actinobacteria | Acidimicrobiia | Acidimicrobiales | Iamiaceae |
|  | *Limisphaera ngatamarikiensis* | 1 | 0 | Verrucomicrobia | Verrucomicrobiae | Verrucomicrobiales | Verrucomicrobia subdivision 3 |
| P4 | uncultured *Desulfobacterium* sp*.* | 2 | 24 | Proteobacteria | Deltaproteobacteria | Desulfobacterales | Desulfobacteraceae |
|  | *Vibrio parahaemolyticus* | 2 | 21 | Proteobacteria | Gammaproteobacteria | Vibrionales | Vibrionaceae |
|  | *Desulfonauticus* sp. | 2 | 16 | Proteobacteria | Deltaproteobacteria | Desulfovibrionales | Desulfohalobiaceae |
|  | *Dethiobacter alkaliphilus* | 2 | 9 | Firmicutes | Clostridia | Eubacteriales | Syntrophomonadaceae |
| P5 | *Cronbergia siamensis* | 7 | 120 | Cyanobacteria | Gloeobacteria | Nostocales | Nostocaceae |
|  | *Opitutus terrae* | 2 | 17 | Verrucomicrobia | Opitutae | Opitutales | Opitutaceae |
|  | *Gemmata massiliana* | 2 | 17 | Planctomycetes | Planctomycetia | Gemmatales | Gemmataceae |
|  | *Gaiella occulta* | 1 | 0 | Actinobacteria | Rubrobacteria | Gaiellales | Gaiellaceae |
|  | *Desulfovirga adipica* | 1 | 0 | Proteobacteria | Deltaproteobacteria | Syntrophobacterales | Syntrophobacteraceae |
|  | *Desulfomonile tiedjei* | 1 | 0 | Proteobacteria | Deltaproteobacteria | Syntrophobacterales | Syntrophaceae |
|  | *Geminisphaera colitermitum* | 1 | 0 | Verrucomicrobia | Opitutae | Opitutales | Opitutaceae |
|  | *Tellurimicrobium multivorans* | 1 | 0 | Acidobacteria | Blastocatellia | Blastocatellales | Blastocatellaceae |

**Supplementary Table S7: The community membership of the association networks of top abundant bacterial species in Indonesia’s soil microbiomes**

| **Community** | **Species** | **Node degree** | **Betweenness** | **Phylum** | **Class** | **Order** | **Family** |
| --- | --- | --- | --- | --- | --- | --- | --- |
| I1 | *Escherichia coli* | 2 | 26 | Proteobacteria | Gammaproteobacteria | Enterobacterales | Enterobacteriaceae |
|  | *Acidisphaera rubrifaciens* | 2 | 14 | Proteobacteria | Alphaproteobacteria | Rhodospirillales | Acetobacteraceae |
|  | *Azospirillum* sp. | 1 | 0 | Proteobacteria | Alphaproteobacteria | Rhodospirillales | Rhodospirillaceae |
| I2 | uncultured bacterium 5G12 | 4 | 39 | Actinobacteria | Actinobacteria | Gaiellales | Rubrobacteridae |
|  | *Acetobacter pomorum* | 1 | 0 | Proteobacteria | Alphaproteobacteria | Rhodospirillales | Acetobacteraceae |
|  | *Streptomyces griseocarneus* | 1 | 0 | Actinobacteria | Actinobacteria | Streptomycetales | Streptomycetaceae |
|  | *Burkholderia pseudomallei* | 1 | 0 | Proteobacteria | Betaproteobacteria | Burkholderiales | Burkholderiaceae |
| I3 | *Acidibacter ferrireducens* | 8 | 93 | Proteobacteria | Gammaproteobacteria |  |  |
|  | *Virgibacillus profundi* | 1 | 0 | Firmicutes | Bacilli | Bacillales | Bacillaceae |
|  | *Streptomyces himastatinicus* | 1 | 0 | Actinobacteria | Actinobacteria | Streptomycetales | Streptomycetaceae |
|  | *Brucella suis* | 1 | 0 | Proteobacteria | Alphaproteobacteria | Rhizobiales | Brucellaceae |
|  | *Vibrio parahaemolyticus* | 1 | 0 | Proteobacteria | Gammaproteobacteria | Vibrionales | Vibrionaceae |
|  | *Dethiobacter alkaliphilus* | 1 | 0 | Firmicutes | Clostridia | Eubacteriales | Syntrophomonadaceae |
| I4 | *Klebsiella pneumoniae* | 3 | 27 | Proteobacteria | Gammaproteobacteria | Enterobacterales | Enterobacteriaceae |
|  | *Acinetobacter baumannii* | 1 | 0 | Proteobacteria | Gammaproteobacteria | Pseudomonadales | Moraxellaceae |
|  | *Carboxydocella* sp. | 1 | 0 | Firmicutes | Clostridia | Eubacteriales | Eubacteriales Family XVI. Incertae Sedis |

**Supplementary Table S8: The functional profiles of the enriched soil bacterial species related to nitrogen biogeochemical pathway.** Only the enriched species with available genomes were displayed.

| **Species** | **Nitrogen fixation** | | **Assimilatory nitrate reduction** | | | **Assimilatory nitrite reduction** | | **Dissimilatory nitrate reduction** | | **Dissimilatory nitrite reduction** | | **Denitrification** | | | | **Nitrification** | |
| --- | --- | --- | --- | --- | --- | --- | --- | --- | --- | --- | --- | --- | --- | --- | --- | --- | --- |
|  | **nifDKH** | **vnfDKGH** | **narB** | **NR** | **nasAB** | **nit-6** | **nirA** | **narGHI** | **napAB** | **nirBD** | **nrfAH** | **nirS** | **nirK** | **norBC** | **nosZ** | **pmo-amo** | **hao** |
| *Acetobacter pomorum* | 0 | 0 | 0 | 0 | 0 | 0 | 0 | 0 | 0 | 0 | 0 | 0 | 0 | 0 | 0 | 0 | 0 |
| *Acetobacteroides hydrogenigenes* | 0 | 0 | 0 | 0 | 0 | 0 | 0 | 0 | 0 | 0 | 2 | 0 | 0 | 0 | 0 | 0 | 0 |
| *Acidisphaera rubrifaciens* | 0 | 0 | 0 | 0 | 0 | 0 | 1 | 0 | 0 | 0 | 0 | 0 | 0 | 0 | 0 | 0 | 0 |
| *Acinetobacter baumannii* | 0 | 0 | 0 | 0 | 1 | 0 | 0 | 0 | 0 | 3 | 0 | 0 | 0 | 0 | 0 | 0 | 0 |
| *Alkanindiges illinoisensis* | 0 | 0 | 0 | 0 | 1 | 0 | 0 | 0 | 0 | 3 | 0 | 0 | 0 | 0 | 0 | 0 | 0 |
| *Anaerocolumna aminovalerica* | 1 | 0 | 0 | 0 | 0 | 0 | 0 | 0 | 0 | 0 | 0 | 0 | 0 | 0 | 0 | 0 | 0 |
| *Anaerolinea thermophila* | 0 | 0 | 0 | 0 | 0 | 0 | 0 | 0 | 0 | 0 | 2 | 1 | 0 | 0 | 0 | 0 | 0 |
| *Anaeromyxobacter dehalogenans* | 0 | 0 | 0 | 0 | 0 | 0 | 0 | 3 | 1 | 0 | 4 | 0 | 0 | 2 | 1 | 0 | 0 |
| *Aquicella siphonis* | 0 | 0 | 0 | 0 | 0 | 0 | 0 | 0 | 0 | 0 | 0 | 0 | 0 | 1 | 0 | 0 | 0 |
| *Aquisphaera giovannonii* | 0 | 0 | 0 | 0 | 0 | 0 | 0 | 0 | 0 | 0 | 0 | 0 | 0 | 1 | 0 | 0 | 0 |
| *Azospirillum* sp. CAG:260 | 0 | 0 | 0 | 0 | 0 | 0 | 0 | 0 | 0 | 0 | 0 | 0 | 0 | 0 | 0 | 0 | 0 |
| *Bacillus cereus* | 0 | 0 | 0 | 0 | 0 | 0 | 1 | 3 | 0 | 2 | 0 | 0 | 0 | 0 | 0 | 0 | 0 |
| *Bacillus thuringiensis* | 0 | 0 | 0 | 0 | 0 | 0 | 1 | 3 | 0 | 2 | 0 | 0 | 0 | 0 | 0 | 0 | 0 |
| *Bradyrhizobium erythrophlei* | 0 | 0 | 0 | 0 | 1 | 0 | 1 | 0 | 0 | 1 | 0 | 0 | 0 | 0 | 0 | 0 | 0 |
| *Brevibacillus fluminis* | 0 | 0 | 0 | 0 | 0 | 0 | 1 | 0 | 0 | 0 | 0 | 0 | 0 | 0 | 0 | 0 | 0 |
| *Brucella suis* | 0 | 0 | 0 | 0 | 0 | 0 | 0 | 3 | 0 | 0 | 0 | 0 | 1 | 2 | 1 | 0 | 0 |
| *Burkholderia cenocepacia* | 0 | 0 | 0 | 0 | 0 | 0 | 0 | 0 | 0 | 2 | 0 | 0 | 0 | 0 | 0 | 0 | 0 |
| *Burkholderia cepacia* | 0 | 0 | 0 | 0 | 0 | 0 | 0 | 0 | 0 | 2 | 0 | 0 | 0 | 0 | 0 | 0 | 0 |
| *Burkholderia pseudomallei* | 0 | 0 | 0 | 0 | 1 | 0 | 0 | 6 | 0 | 4 | 0 | 0 | 2 | 2 | 1 | 0 | 0 |
| *Burkholderia pseudomultivorans* | 0 | 0 | 0 | 0 | 0 | 0 | 0 | 3 | 0 | 2 | 0 | 0 | 0 | 0 | 0 | 0 | 0 |
| *Bythopirellula goksoyri* | 0 | 0 | 0 | 0 | 0 | 0 | 0 | 0 | 0 | 2 | 2 | 0 | 0 | 1 | 0 | 0 | 0 |
| *Candidatus* Giovannonibacteria bacterium RIFCSPHIGHO2_12_FULL_44_42 | 0 | 0 | 0 | 0 | 0 | 0 | 0 | 0 | 0 | 0 | 0 | 0 | 0 | 0 | 0 | 0 | 0 |
| *Candidatus Koribacter versatilis* | 0 | 0 | 0 | 0 | 0 | 0 | 0 | 0 | 0 | 0 | 0 | 0 | 0 | 1 | 0 | 0 | 0 |
| *Candidatus* Levybacteria bacterium GW2011_GWB1_41_21 | 0 | 0 | 0 | 0 | 0 | 0 | 0 | 0 | 0 | 0 | 0 | 0 | 0 | 0 | 0 | 0 | 0 |
| *Candidatus* Liptonbacteria bacterium CG11_big_fil_rev_8_21_14_0_20_35_14 | 0 | 0 | 0 | 0 | 0 | 0 | 0 | 0 | 0 | 0 | 0 | 0 | 0 | 0 | 0 | 0 | 0 |
| *Candidatus* Rokubacteria bacterium | 0 | 0 | 0 | 0 | 0 | 0 | 1 | 3 | 2 | 0 | 2 | 1 | 1 | 0 | 0 | 0 | 0 |
| *Candidatus Solibacter usitatus* | 0 | 0 | 0 | 0 | 0 | 0 | 0 | 0 | 0 | 1 | 0 | 0 | 0 | 2 | 0 | 0 | 0 |
| *Candidatus Sulfopaludibacter* sp. SbA4 | 0 | 0 | 0 | 0 | 0 | 0 | 0 | 3 | 0 | 0 | 0 | 0 | 0 | 0 | 0 | 0 | 0 |
| *Candidatus Sulfotelmatobacter kueseliae* | 0 | 0 | 0 | 0 | 0 | 0 | 0 | 1 | 0 | 0 | 0 | 0 | 0 | 0 | 0 | 0 | 0 |
| *Candidatus* Woesebacteria bacterium RBG_16_36_11 | 0 | 0 | 0 | 0 | 0 | 0 | 0 | 0 | 0 | 0 | 0 | 0 | 0 | 0 | 0 | 0 | 0 |
| *Candidatus* Zambryskibacteria bacterium CG10_big_fil_rev_8_21_14_0_10_42_12 | 0 | 0 | 0 | 0 | 0 | 0 | 0 | 0 | 0 | 0 | 0 | 0 | 0 | 0 | 0 | 0 | 0 |
| *Carboxydocella* sp. JDF658 | 3 | 0 | 0 | 0 | 0 | 0 | 0 | 0 | 1 | 0 | 4 | 0 | 0 | 0 | 0 | 0 | 0 |
| *Chitinispirillum alkaliphilum* | 0 | 0 | 0 | 0 | 0 | 0 | 0 | 0 | 0 | 0 | 0 | 0 | 0 | 0 | 0 | 0 | 0 |
| *Chlamydia trachomatis* | 0 | 0 | 0 | 0 | 0 | 0 | 0 | 0 | 0 | 0 | 0 | 0 | 0 | 0 | 0 | 0 | 0 |
| *Chromobacterium* sp. ATCC 53434 | 0 | 0 | 0 | 0 | 1 | 0 | 0 | 3 | 0 | 2 | 0 | 0 | 0 | 1 | 0 | 0 | 0 |
| *Chthoniobacter flavus* | 0 | 0 | 0 | 0 | 0 | 0 | 2 | 0 | 0 | 1 | 2 | 0 | 1 | 0 | 0 | 0 | 0 |
| *Clostridioides difficile* | 0 | 0 | 0 | 0 | 0 | 0 | 0 | 0 | 0 | 0 | 0 | 0 | 0 | 0 | 0 | 0 | 0 |
| *Clostridium aurantibutyricum* | 3 | 0 | 0 | 0 | 0 | 0 | 0 | 0 | 0 | 0 | 0 | 0 | 0 | 0 | 0 | 0 | 0 |
| *Clostridium bolteae* | 0 | 0 | 0 | 0 | 0 | 0 | 0 | 0 | 0 | 0 | 0 | 0 | 0 | 0 | 0 | 0 | 0 |
| *Clostridium botulinum* | 1 | 0 | 0 | 0 | 0 | 0 | 0 | 0 | 0 | 0 | 0 | 0 | 0 | 0 | 0 | 0 | 0 |
| *Clostridium cadaveris* | 0 | 0 | 0 | 0 | 0 | 0 | 0 | 0 | 0 | 0 | 0 | 0 | 0 | 0 | 0 | 0 | 0 |
| *Clostridium cellulosi* | 1 | 0 | 0 | 0 | 0 | 0 | 0 | 0 | 0 | 0 | 0 | 0 | 0 | 0 | 0 | 0 | 0 |
| *Clostridium leptum* | 0 | 0 | 0 | 0 | 0 | 0 | 0 | 0 | 0 | 0 | 0 | 0 | 0 | 0 | 0 | 0 | 0 |
| *Clostridium* sp. BL8 | 0 | 0 | 0 | 0 | 0 | 0 | 0 | 0 | 0 | 0 | 0 | 0 | 0 | 0 | 0 | 0 | 0 |
| *Comamonas kerstersii* | 0 | 0 | 0 | 0 | 1 | 0 | 0 | 0 | 2 | 2 | 0 | 0 | 0 | 0 | 0 | 0 | 0 |
| *Comamonas testosteroni* | 0 | 0 | 0 | 0 | 1 | 0 | 0 | 0 | 0 | 2 | 0 | 0 | 0 | 0 | 0 | 0 | 0 |
| *Crenothrix polyspora* | 0 | 0 | 0 | 0 | 0 | 0 | 0 | 0 | 0 | 2 | 0 | 0 | 1 | 0 | 0 | 6 | 1 |
| *Cyanobium gracile* | 0 | 0 | 0 | 0 | 0 | 0 | 1 | 0 | 0 | 0 | 0 | 0 | 0 | 0 | 0 | 0 | 0 |
| *Delftia tsuruhatensis* | 0 | 0 | 0 | 0 | 1 | 0 | 0 | 3 | 0 | 3 | 0 | 0 | 0 | 0 | 0 | 0 | 0 |
| *Desulfomonile tiedjei* | 3 | 0 | 0 | 0 | 0 | 0 | 0 | 6 | 1 | 0 | 0 | 0 | 0 | 1 | 1 | 0 | 0 |
| *Desulfonauticus* sp. 38_4375 | 0 | 0 | 0 | 0 | 0 | 0 | 0 | 1 | 0 | 0 | 0 | 0 | 0 | 0 | 0 | 0 | 0 |
| *Desulfovibrio putealis* | 6 | 0 | 0 | 0 | 0 | 0 | 0 | 1 | 0 | 0 | 2 | 0 | 0 | 1 | 0 | 0 | 0 |
| *Dethiobacter alkaliphilus* | 3 | 0 | 0 | 0 | 0 | 0 | 0 | 0 | 0 | 0 | 4 | 0 | 0 | 2 | 0 | 0 | 0 |
| *Dictyobacter aurantiacus* | 0 | 0 | 0 | 0 | 0 | 0 | 0 | 0 | 0 | 1 | 0 | 0 | 0 | 0 | 0 | 0 | 0 |
| *Dinghuibacter silviterrae* | 0 | 0 | 1 | 0 | 0 | 0 | 0 | 0 | 0 | 2 | 0 | 0 | 0 | 0 | 0 | 0 | 0 |
| *Diplorickettsia massiliensis* | 0 | 0 | 0 | 0 | 0 | 0 | 0 | 0 | 0 | 0 | 0 | 0 | 0 | 0 | 0 | 0 | 0 |
| *Dorea longicatena* | 0 | 0 | 0 | 0 | 0 | 0 | 0 | 0 | 0 | 0 | 0 | 0 | 0 | 0 | 0 | 0 | 0 |
| *Duganella ginsengisoli* | 0 | 0 | 0 | 0 | 1 | 0 | 0 | 0 | 0 | 3 | 0 | 0 | 0 | 0 | 0 | 0 | 0 |
| *Dyella japonica* | 0 | 0 | 0 | 0 | 1 | 0 | 0 | 0 | 2 | 3 | 0 | 0 | 1 | 0 | 0 | 0 | 0 |
| *Dyella marensis* | 0 | 0 | 0 | 0 | 1 | 0 | 0 | 0 | 2 | 2 | 0 | 0 | 0 | 0 | 0 | 0 | 0 |
| *Elizabethkingia anophelis* | 0 | 0 | 0 | 0 | 0 | 0 | 0 | 0 | 0 | 0 | 0 | 0 | 0 | 1 | 0 | 0 | 0 |
| *Endomicrobium proavitum* | 1 | 0 | 0 | 0 | 0 | 0 | 0 | 0 | 0 | 0 | 0 | 0 | 0 | 0 | 0 | 0 | 0 |
| *Enterobacter cancerogenus* | 0 | 0 | 0 | 0 | 1 | 0 | 0 | 6 | 0 | 4 | 0 | 0 | 0 | 0 | 0 | 0 | 0 |
| *Enterococcus faecalis* | 0 | 0 | 0 | 0 | 0 | 0 | 0 | 0 | 0 | 0 | 0 | 0 | 0 | 0 | 0 | 0 | 0 |
| *Erythrobacter luteus* | 0 | 0 | 0 | 0 | 0 | 0 | 0 | 0 | 0 | 0 | 0 | 0 | 0 | 0 | 0 | 0 | 0 |
| *Escherichia coli* | 0 | 0 | 0 | 0 | 0 | 0 | 0 | 6 | 2 | 2 | 1 | 0 | 0 | 0 | 0 | 0 | 0 |
| *Fimbriimonas ginsengisoli* | 0 | 0 | 0 | 0 | 1 | 0 | 1 | 0 | 0 | 1 | 0 | 0 | 0 | 0 | 0 | 0 | 0 |
| *Flavisolibacter tropicus* | 0 | 0 | 0 | 0 | 0 | 0 | 0 | 0 | 0 | 0 | 0 | 0 | 0 | 2 | 2 | 0 | 0 |
| *Fonticella tunisiensis* | 0 | 0 | 0 | 0 | 0 | 0 | 0 | 0 | 0 | 0 | 0 | 0 | 0 | 0 | 0 | 0 | 0 |
| *Gaiella occulta* | 0 | 0 | 0 | 0 | 0 | 0 | 0 | 3 | 0 | 0 | 0 | 0 | 1 | 0 | 0 | 0 | 0 |
| *Gardnerella vaginalis* | 0 | 0 | 0 | 0 | 0 | 0 | 0 | 0 | 0 | 0 | 0 | 0 | 0 | 0 | 0 | 0 | 0 |
| *Geminisphaera colitermitum* | 7 | 0 | 0 | 0 | 0 | 0 | 0 | 0 | 0 | 0 | 2 | 0 | 0 | 0 | 1 | 0 | 0 |
| *Gemmata massiliana* | 0 | 0 | 0 | 0 | 0 | 0 | 0 | 0 | 0 | 2 | 0 | 0 | 0 | 0 | 0 | 0 | 0 |
| *Gemmatimonas phototrophica* | 0 | 0 | 0 | 0 | 0 | 0 | 0 | 0 | 0 | 0 | 0 | 0 | 0 | 0 | 0 | 0 | 0 |
| *Geobacter pelophilus* | 3 | 0 | 0 | 0 | 0 | 0 | 0 | 1 | 0 | 0 | 3 | 0 | 0 | 3 | 0 | 0 | 0 |
| *Haliangium ochraceum* | 0 | 0 | 0 | 0 | 0 | 0 | 0 | 0 | 0 | 0 | 0 | 0 | 0 | 0 | 0 | 0 | 0 |
| *Klebsiella oxytoca* | 3 | 0 | 0 | 0 | 1 | 0 | 0 | 6 | 0 | 4 | 0 | 0 | 0 | 0 | 0 | 0 | 0 |
| *Klebsiella pneumoniae* | 0 | 0 | 0 | 0 | 1 | 0 | 0 | 7 | 0 | 5 | 0 | 0 | 0 | 0 | 0 | 0 | 0 |
| *Ktedonobacter racemifer* | 0 | 0 | 0 | 0 | 0 | 0 | 0 | 0 | 0 | 0 | 0 | 0 | 0 | 0 | 0 | 0 | 0 |
| *Lactococcus garvieae* | 0 | 0 | 0 | 0 | 0 | 0 | 0 | 0 | 0 | 0 | 0 | 0 | 0 | 0 | 0 | 0 | 0 |
| *Lactococcus lactis* | 0 | 0 | 0 | 0 | 0 | 0 | 0 | 0 | 0 | 0 | 0 | 0 | 0 | 0 | 0 | 0 | 0 |
| *Legionella pneumophila* | 0 | 0 | 0 | 0 | 0 | 0 | 0 | 0 | 0 | 0 | 0 | 0 | 0 | 1 | 0 | 0 | 0 |
| *Lentimicrobium saccharophilum* | 0 | 0 | 0 | 0 | 0 | 0 | 0 | 0 | 0 | 0 | 2 | 0 | 1 | 0 | 0 | 0 | 0 |
| *Limisphaera ngatamarikiensis* | 0 | 0 | 0 | 0 | 0 | 0 | 0 | 0 | 0 | 0 | 2 | 0 | 0 | 0 | 0 | 0 | 0 |
| *Listeria monocytogenes* | 0 | 0 | 0 | 0 | 0 | 0 | 0 | 0 | 0 | 0 | 0 | 0 | 0 | 0 | 0 | 0 | 0 |
| *Listeria seeligeri* | 0 | 0 | 0 | 0 | 0 | 0 | 0 | 0 | 0 | 0 | 0 | 0 | 0 | 0 | 0 | 0 | 0 |
| *Litorilinea aerophila* | 0 | 0 | 1 | 0 | 1 | 0 | 1 | 2 | 2 | 0 | 0 | 0 | 0 | 0 | 1 | 0 | 0 |
| *Longilinea arvoryzae* | 0 | 0 | 0 | 0 | 0 | 0 | 0 | 0 | 0 | 0 | 0 | 1 | 0 | 0 | 0 | 0 | 0 |
| *Luteitalea pratensis* | 0 | 0 | 0 | 0 | 1 | 0 | 0 | 0 | 0 | 1 | 2 | 0 | 0 | 2 | 1 | 0 | 0 |
| *Magnetospirillum* sp. XM-1 | 3 | 0 | 0 | 0 | 0 | 0 | 0 | 1 | 2 | 2 | 0 | 2 | 0 | 2 | 1 | 0 | 0 |
| *Microcystis aeruginosa* | 0 | 0 | 1 | 0 | 0 | 0 | 1 | 0 | 0 | 0 | 0 | 0 | 0 | 0 | 0 | 0 | 0 |
| *Microvirga flocculans* | 0 | 0 | 0 | 0 | 0 | 0 | 0 | 0 | 2 | 0 | 0 | 0 | 1 | 0 | 0 | 0 | 0 |
| *Minicystis rosea* | 0 | 0 | 0 | 0 | 0 | 0 | 0 | 0 | 0 | 1 | 0 | 0 | 0 | 0 | 0 | 0 | 0 |
| *Moraxella osloensis* | 0 | 0 | 0 | 0 | 1 | 0 | 0 | 0 | 0 | 2 | 0 | 0 | 0 | 0 | 0 | 0 | 0 |
| *Morganella morganii* | 0 | 0 | 0 | 0 | 0 | 0 | 0 | 3 | 0 | 2 | 0 | 0 | 0 | 0 | 0 | 0 | 0 |
| *Mycobacteroides abscessus* | 0 | 0 | 0 | 0 | 0 | 0 | 0 | 0 | 0 | 2 | 0 | 0 | 0 | 0 | 0 | 0 | 0 |
| *Nitrospira japonica* | 0 | 0 | 0 | 0 | 0 | 0 | 0 | 4 | 1 | 1 | 0 | 0 | 3 | 0 | 0 | 0 | 0 |
| *Novimethylophilus kurashikiensis* | 0 | 0 | 0 | 0 | 1 | 0 | 0 | 2 | 0 | 2 | 0 | 0 | 1 | 2 | 1 | 0 | 0 |
| *Novosphingobium rosa* | 0 | 0 | 0 | 0 | 0 | 0 | 0 | 0 | 0 | 2 | 0 | 0 | 0 | 0 | 0 | 0 | 0 |
| *Opitutus terrae* | 0 | 0 | 0 | 0 | 0 | 0 | 1 | 0 | 0 | 0 | 2 | 0 | 1 | 0 | 1 | 0 | 0 |
| *Oscillochloris trichoides* | 3 | 0 | 0 | 0 | 0 | 0 | 0 | 0 | 0 | 0 | 2 | 0 | 0 | 0 | 0 | 0 | 0 |
| *Paenarthrobacter nicotinovorans* | 0 | 0 | 0 | 0 | 0 | 0 | 0 | 0 | 0 | 2 | 0 | 0 | 0 | 0 | 0 | 0 | 0 |
| *Paenibacillus herberti* | 0 | 0 | 0 | 0 | 0 | 0 | 1 | 0 | 0 | 0 | 0 | 0 | 0 | 0 | 0 | 0 | 0 |
| *Paeniclostridium sordellii* | 1 | 0 | 0 | 0 | 0 | 0 | 0 | 0 | 0 | 0 | 0 | 0 | 0 | 0 | 0 | 0 | 0 |
| *Paludisphaera borealis* | 0 | 0 | 0 | 0 | 0 | 0 | 1 | 0 | 0 | 1 | 0 | 0 | 0 | 1 | 0 | 0 | 0 |
| *Pantoea ananatis* | 0 | 0 | 0 | 0 | 1 | 0 | 0 | 1 | 0 | 2 | 0 | 0 | 0 | 0 | 0 | 0 | 0 |
| *Paraburkholderia nodosa* | 3 | 0 | 0 | 0 | 0 | 0 | 0 | 6 | 0 | 2 | 0 | 0 | 1 | 1 | 1 | 0 | 0 |
| *Paraburkholderia susongensis* | 0 | 0 | 0 | 0 | 0 | 0 | 0 | 0 | 0 | 2 | 0 | 0 | 0 | 0 | 0 | 0 | 0 |
| *Paracoccus denitrificans* | 0 | 0 | 0 | 0 | 1 | 0 | 0 | 3 | 2 | 2 | 0 | 1 | 0 | 2 | 1 | 0 | 0 |
| *Paracoccus yeei* | 0 | 0 | 0 | 0 | 1 | 0 | 0 | 3 | 0 | 2 | 0 | 0 | 0 | 2 | 1 | 0 | 0 |
| *Parafilimonas terrae* | 0 | 0 | 0 | 0 | 0 | 0 | 0 | 0 | 0 | 2 | 0 | 0 | 1 | 0 | 1 | 0 | 0 |
| *Pararhodospirillum photometricum* | 8 | 0 | 0 | 0 | 0 | 0 | 0 | 0 | 0 | 0 | 0 | 0 | 0 | 0 | 0 | 0 | 0 |
| *Parvimonas micra* | 0 | 0 | 0 | 0 | 0 | 0 | 0 | 0 | 0 | 0 | 0 | 0 | 0 | 0 | 0 | 0 | 0 |
| *Petrimonas mucosa* | 0 | 0 | 0 | 0 | 0 | 0 | 0 | 0 | 0 | 0 | 2 | 0 | 0 | 1 | 0 | 0 | 0 |
| *Pirellula staleyi* | 0 | 0 | 0 | 0 | 0 | 0 | 1 | 0 | 1 | 0 | 0 | 0 | 0 | 0 | 0 | 0 | 0 |
| *Proteiniphilum saccharofermentans* | 0 | 0 | 0 | 0 | 0 | 0 | 0 | 0 | 0 | 0 | 2 | 0 | 0 | 1 | 0 | 0 | 0 |
| *Pseudoclostridium thermosuccinogenes* | 4 | 0 | 0 | 0 | 0 | 0 | 0 | 0 | 0 | 0 | 0 | 0 | 0 | 0 | 0 | 0 | 0 |
| *Pseudoduganella eburnea* | 0 | 0 | 0 | 0 | 1 | 0 | 0 | 0 | 0 | 3 | 0 | 0 | 1 | 1 | 1 | 0 | 0 |
| *Pseudoflavonifractor capillosus* | 0 | 0 | 0 | 0 | 0 | 0 | 0 | 0 | 0 | 0 | 0 | 0 | 0 | 0 | 0 | 0 | 0 |
| *Pseudomonas flexibilis* | 0 | 0 | 0 | 0 | 1 | 0 | 0 | 6 | 2 | 3 | 0 | 2 | 0 | 2 | 1 | 0 | 0 |
| *Pseudomonas lini* | 0 | 0 | 0 | 0 | 1 | 0 | 0 | 3 | 0 | 4 | 0 | 1 | 0 | 2 | 1 | 0 | 0 |
| *Reyranella soli* | 0 | 0 | 0 | 0 | 1 | 0 | 1 | 0 | 0 | 2 | 0 | 0 | 0 | 0 | 0 | 0 | 0 |
| *Rhodobacter* sp. LPB0142 | 0 | 0 | 0 | 0 | 1 | 0 | 0 | 1 | 0 | 2 | 0 | 1 | 0 | 2 | 1 | 0 | 0 |
| *Rhodopila globiformis* | 4 | 3 | 0 | 0 | 0 | 0 | 0 | 0 | 0 | 0 | 0 | 0 | 1 | 2 | 1 | 0 | 0 |
| *Rhodovulum sulfidophilum* | 3 | 0 | 0 | 0 | 0 | 0 | 0 | 2 | 0 | 0 | 0 | 0 | 0 | 0 | 0 | 0 | 0 |
| *Rickettsia prowazekii* | 0 | 0 | 0 | 0 | 0 | 0 | 0 | 0 | 0 | 0 | 0 | 0 | 0 | 0 | 0 | 0 | 0 |
| *Roseiarcus fermentans* | 4 | 0 | 0 | 0 | 1 | 0 | 0 | 0 | 0 | 2 | 0 | 0 | 1 | 0 | 0 | 0 | 0 |
| *Rudaea cellulosilytica* | 0 | 0 | 0 | 0 | 1 | 0 | 0 | 0 | 0 | 2 | 0 | 0 | 0 | 0 | 0 | 0 | 0 |
| *Rugosibacter aromaticivorans* | 0 | 0 | 0 | 0 | 1 | 0 | 0 | 0 | 0 | 2 | 0 | 0 | 0 | 1 | 0 | 0 | 0 |
| *Ruminiclostridium cellobioparum* | 5 | 0 | 0 | 0 | 0 | 0 | 0 | 0 | 0 | 0 | 0 | 0 | 0 | 0 | 0 | 0 | 0 |
| *Ruminococcus gnavus* | 0 | 0 | 0 | 0 | 0 | 0 | 0 | 0 | 0 | 0 | 0 | 0 | 0 | 0 | 0 | 0 | 0 |
| *Salmonella enterica* | 0 | 0 | 0 | 0 | 0 | 0 | 0 | 6 | 2 | 2 | 1 | 0 | 0 | 0 | 0 | 0 | 0 |
| *Sedimentibacter saalensis* | 0 | 0 | 0 | 0 | 0 | 0 | 0 | 0 | 0 | 0 | 1 | 0 | 0 | 0 | 0 | 0 | 0 |
| *Shewanella benthica* | 0 | 0 | 0 | 0 | 0 | 0 | 0 | 0 | 3 | 1 | 2 | 0 | 0 | 0 | 0 | 0 | 0 |
| *Singulisphaera acidiphila* | 0 | 0 | 0 | 0 | 0 | 0 | 1 | 0 | 0 | 0 | 0 | 0 | 0 | 0 | 0 | 0 | 0 |
| *Sinosporangium album* | 0 | 0 | 0 | 0 | 0 | 0 | 0 | 0 | 0 | 0 | 0 | 0 | 0 | 0 | 0 | 0 | 0 |
| *Solitalea koreensis* | 0 | 0 | 0 | 0 | 0 | 0 | 0 | 0 | 0 | 0 | 0 | 0 | 0 | 0 | 0 | 0 | 0 |
| *Sorangium cellulosum* | 0 | 0 | 0 | 0 | 1 | 0 | 0 | 0 | 2 | 4 | 2 | 0 | 0 | 2 | 0 | 0 | 0 |
| *Sphingorhabdus contaminans* | 0 | 0 | 0 | 0 | 0 | 0 | 0 | 0 | 0 | 0 | 0 | 0 | 0 | 0 | 0 | 0 | 0 |
| *Sporomusa malonica* | 3 | 0 | 0 | 0 | 0 | 0 | 1 | 0 | 0 | 0 | 4 | 0 | 0 | 0 | 0 | 0 | 0 |
| *Staphylococcus aureus* | 0 | 0 | 0 | 0 | 0 | 0 | 0 | 3 | 0 | 2 | 0 | 0 | 0 | 0 | 0 | 0 | 0 |
| *Stenotrophomonas maltophilia* | 0 | 0 | 0 | 0 | 0 | 0 | 0 | 3 | 0 | 0 | 0 | 0 | 0 | 0 | 0 | 0 | 0 |
| *Streptococcus pneumoniae* | 0 | 0 | 0 | 0 | 0 | 0 | 0 | 0 | 0 | 0 | 0 | 0 | 0 | 0 | 0 | 0 | 0 |
| *Streptococcus ruminantium* | 0 | 0 | 0 | 0 | 0 | 0 | 0 | 0 | 0 | 0 | 0 | 0 | 0 | 0 | 0 | 0 | 0 |
| *Streptococcus thermophilus* | 0 | 0 | 0 | 0 | 0 | 0 | 0 | 0 | 0 | 0 | 0 | 0 | 0 | 0 | 0 | 0 | 0 |
| *Streptomyces cinnamoneus* | 0 | 0 | 0 | 0 | 1 | 0 | 0 | 0 | 0 | 2 | 0 | 0 | 0 | 0 | 0 | 0 | 0 |
| *Streptomyces himastatinicus* | 0 | 0 | 0 | 0 | 0 | 0 | 0 | 0 | 0 | 3 | 0 | 0 | 0 | 0 | 0 | 0 | 0 |
| *Sutterella* sp. CAG:521 | 0 | 0 | 0 | 0 | 0 | 0 | 0 | 0 | 0 | 0 | 0 | 0 | 0 | 0 | 0 | 0 | 0 |
| *Synechococcus* sp. WH 8109 | 0 | 0 | 1 | 0 | 0 | 0 | 1 | 0 | 0 | 0 | 0 | 0 | 0 | 0 | 0 | 0 | 0 |
| *Syntrophobacter* sp. SbD1 | 3 | 0 | 0 | 0 | 0 | 0 | 0 | 1 | 0 | 0 | 0 | 0 | 0 | 0 | 0 | 0 | 0 |
| *Syntrophus aciditrophicus* | 0 | 0 | 0 | 0 | 0 | 0 | 0 | 0 | 0 | 0 | 2 | 0 | 0 | 0 | 0 | 0 | 0 |
| *Taylorella asinigenitalis* | 0 | 0 | 0 | 0 | 0 | 0 | 0 | 0 | 0 | 0 | 0 | 0 | 1 | 0 | 0 | 0 | 0 |
| *Telmatospirillum siberiense* | 10 | 0 | 0 | 0 | 1 | 0 | 0 | 0 | 2 | 2 | 2 | 0 | 0 | 0 | 0 | 0 | 0 |
| *Terrimicrobium sacchariphilum* | 3 | 0 | 0 | 0 | 0 | 0 | 0 | 0 | 0 | 0 | 0 | 0 | 0 | 0 | 0 | 0 | 0 |
| *Thermotalea metallivorans* | 0 | 0 | 0 | 0 | 0 | 0 | 0 | 0 | 0 | 0 | 0 | 0 | 0 | 0 | 0 | 0 | 0 |
| *Thermus thermophilus* | 0 | 0 | 0 | 0 | 0 | 0 | 0 | 0 | 0 | 0 | 0 | 0 | 0 | 0 | 0 | 0 | 0 |
| *Thiobacillus thioparus* | 0 | 0 | 0 | 0 | 1 | 0 | 0 | 4 | 0 | 2 | 0 | 0 | 1 | 1 | 1 | 0 | 0 |
| *Tissierella praeacuta* | 1 | 0 | 0 | 0 | 0 | 0 | 0 | 0 | 0 | 0 | 0 | 0 | 0 | 0 | 0 | 0 | 0 |
| *Vibrio parahaemolyticus* | 0 | 0 | 0 | 0 | 1 | 0 | 0 | 0 | 2 | 4 | 1 | 0 | 0 | 0 | 0 | 0 | 0 |
| *Virgibacillus profundi* | 0 | 0 | 0 | 0 | 0 | 0 | 0 | 6 | 0 | 0 | 0 | 0 | 1 | 0 | 0 | 0 | 0 |
| *Vulgatibacter incomptus* | 0 | 0 | 0 | 0 | 0 | 0 | 0 | 0 | 0 | 0 | 2 | 0 | 0 | 0 | 0 | 0 | 0 |
| *Xanthomonas citri* | 0 | 0 | 0 | 0 | 0 | 0 | 0 | 0 | 0 | 0 | 0 | 0 | 0 | 0 | 0 | 0 | 0 |
| *Yangia pacifica* | 0 | 0 | 0 | 0 | 1 | 0 | 0 | 0 | 2 | 2 | 0 | 0 | 0 | 0 | 0 | 0 | 0 |
| *Zavarzinella formosa* | 0 | 0 | 0 | 0 | 0 | 0 | 1 | 0 | 0 | 3 | 0 | 0 | 0 | 0 | 0 | 0 | 0 |

**Supplementary Table S9: The functional profiles of the enriched soil bacterial species related to sulphur biogeochemical pathway.** Only the enriched species with available genomes were displayed.

| **Species** | **Assimilatory sulfate reduction** | | | | | | | **Dissimilatory sulfate reduction and oxidation** | | **SOX system** | | | **Sulfite detoxification** | | **Sulfur oxidation to sulfide** | | **Anaerobic sulfate reduction** | |
| --- | --- | --- | --- | --- | --- | --- | --- | --- | --- | --- | --- | --- | --- | --- | --- | --- | --- | --- |
|  | **sat** | **cysNCD** | **cysC** | **PAPSS** | **cysH** | **cysJI** | **sir** | **dsrAB** | **aprAB** | **soxAX** | **soxB** | **soxCD** | **soeABC** | **suox** | **fccAB** | **sqr** | **asrABC** | **fsr** |
| *Acetobacter pomorum* | 1 | 2 | 1 | 0 | 0 | 0 | 0 | 0 | 0 | 0 | 0 | 0 | 0 | 1 | 0 | 0 | 1 | 0 |
| *Acetobacteroides hydrogenigenes* | 0 | 0 | 0 | 0 | 0 | 0 | 0 | 0 | 0 | 0 | 0 | 0 | 0 | 1 | 0 | 0 | 0 | 0 |
| *Acidisphaera rubrifaciens* | 1 | 0 | 1 | 0 | 1 | 0 | 0 | 1 | 2 | 4 | 0 | 0 | 0 | 2 | 0 | 0 | 1 | 0 |
| *Acinetobacter baumannii* | 1 | 2 | 2 | 0 | 0 | 0 | 1 | 0 | 0 | 0 | 0 | 0 | 0 | 1 | 0 | 0 | 2 | 0 |
| *Alkanindiges illinoisensis* | 1 | 2 | 2 | 0 | 0 | 0 | 1 | 0 | 0 | 0 | 0 | 0 | 0 | 0 | 0 | 0 | 3 | 0 |
| *Anaerocolumna aminovalerica* | 0 | 0 | 0 | 0 | 0 | 0 | 0 | 0 | 0 | 0 | 0 | 0 | 0 | 0 | 0 | 0 | 0 | 0 |
| *Anaerolinea thermophila* | 0 | 0 | 0 | 0 | 0 | 0 | 0 | 0 | 0 | 0 | 0 | 0 | 0 | 1 | 0 | 0 | 0 | 0 |
| *Anaeromyxobacter dehalogenans* | 1 | 1 | 0 | 0 | 0 | 0 | 0 | 1 | 2 | 4 | 0 | 0 | 0 | 2 | 0 | 0 | 0 | 0 |
| *Aquicella siphonis* | 0 | 0 | 0 | 1 | 0 | 0 | 0 | 0 | 0 | 0 | 0 | 0 | 0 | 0 | 0 | 0 | 0 | 0 |
| *Aquisphaera giovannonii* | 1 | 2 | 0 | 1 | 1 | 0 | 0 | 0 | 0 | 0 | 0 | 0 | 0 | 0 | 0 | 0 | 0 | 0 |
| *Azospirillum* sp. CAG:260 | 0 | 0 | 0 | 0 | 0 | 0 | 0 | 0 | 0 | 0 | 0 | 0 | 0 | 0 | 0 | 0 | 0 | 0 |
| *Bacillus cereus* | 0 | 2 | 0 | 1 | 0 | 0 | 0 | 0 | 0 | 0 | 0 | 0 | 0 | 1 | 0 | 0 | 2 | 0 |
| *Bacillus thuringiensis* | 0 | 2 | 0 | 1 | 0 | 0 | 0 | 0 | 0 | 0 | 0 | 0 | 0 | 1 | 0 | 0 | 2 | 0 |
| *Bradyrhizobium erythrophlei* | 2 | 3 | 1 | 0 | 0 | 0 | 0 | 2 | 5 | 11 | 0 | 0 | 4 | 0 | 0 | 0 | 2 | 0 |
| *Brevibacillus fluminis* | 0 | 1 | 2 | 1 | 0 | 0 | 0 | 0 | 0 | 0 | 0 | 0 | 0 | 1 | 0 | 0 | 0 | 0 |
| *Brucella suis* | 1 | 3 | 1 | 0 | 0 | 0 | 0 | 0 | 0 | 0 | 0 | 0 | 0 | 0 | 0 | 0 | 1 | 0 |
| *Burkholderia cenocepacia* | 2 | 2 | 3 | 0 | 0 | 0 | 0 | 0 | 0 | 2 | 0 | 0 | 2 | 1 | 0 | 0 | 1 | 0 |
| *Burkholderia cepacia* | 2 | 2 | 3 | 0 | 0 | 0 | 0 | 0 | 0 | 2 | 0 | 0 | 2 | 1 | 0 | 0 | 1 | 0 |
| *Burkholderia pseudomallei* | 2 | 1 | 3 | 0 | 0 | 0 | 0 | 0 | 0 | 0 | 0 | 0 | 0 | 1 | 0 | 0 | 1 | 0 |
| *Burkholderia pseudomultivorans* | 2 | 2 | 3 | 0 | 0 | 0 | 0 | 0 | 0 | 0 | 0 | 0 | 2 | 1 | 0 | 0 | 1 | 0 |
| *Bythopirellula goksoyri* | 2 | 4 | 1 | 1 | 1 | 0 | 0 | 0 | 0 | 0 | 0 | 0 | 1 | 0 | 0 | 0 | 0 | 0 |
| *Candidatus* Giovannonibacteria bacterium RIFCSPHIGHO2_12_FULL_44_42 | 0 | 0 | 0 | 0 | 0 | 0 | 0 | 0 | 0 | 0 | 0 | 0 | 0 | 0 | 0 | 0 | 0 | 0 |
| *Candidatus Koribacter versatilis* | 1 | 1 | 0 | 0 | 0 | 0 | 0 | 0 | 0 | 0 | 0 | 0 | 0 | 0 | 0 | 0 | 0 | 0 |
| *Candidatus* Levybacteria bacterium GW2011_GWB1_41_21 | 0 | 0 | 0 | 0 | 0 | 0 | 0 | 0 | 0 | 0 | 0 | 0 | 0 | 0 | 0 | 0 | 0 | 0 |
| *Candidatus* Liptonbacteria bacterium CG11_big_fil_rev_8_21_14_0_20_35_14 | 0 | 0 | 0 | 0 | 0 | 0 | 0 | 0 | 0 | 0 | 0 | 0 | 0 | 0 | 0 | 0 | 0 | 0 |
| *Candidatus* Rokubacteria bacterium | 1 | 1 | 0 | 2 | 0 | 2 | 1 | 4 | 7 | 8 | 0 | 0 | 4 | 1 | 0 | 0 | 3 | 1 |
| *Candidatus Solibacter usitatus* | 1 | 0 | 0 | 0 | 0 | 0 | 0 | 0 | 0 | 0 | 0 | 0 | 0 | 0 | 0 | 0 | 0 | 0 |
| *Candidatus Sulfopaludibacter* sp. SbA4 | 0 | 1 | 0 | 0 | 0 | 2 | 0 | 0 | 0 | 2 | 0 | 0 | 0 | 0 | 0 | 0 | 0 | 0 |
| *Candidatus Sulfotelmatobacter kueseliae* | 0 | 0 | 0 | 1 | 0 | 1 | 0 | 0 | 0 | 0 | 0 | 0 | 0 | 0 | 0 | 0 | 0 | 0 |
| *Candidatus* Woesebacteria bacterium RBG_16_36_11 | 0 | 0 | 0 | 0 | 0 | 0 | 0 | 0 | 0 | 0 | 0 | 0 | 0 | 0 | 0 | 0 | 0 | 0 |
| *Candidatus* Zambryskibacteria bacterium CG10_big_fil_rev_8_21_14_0_10_42_12 | 0 | 0 | 0 | 0 | 0 | 0 | 0 | 0 | 0 | 0 | 0 | 0 | 0 | 0 | 0 | 0 | 0 | 0 |
| *Carboxydocella* sp. JDF658 | 0 | 0 | 0 | 1 | 0 | 0 | 1 | 1 | 0 | 0 | 0 | 0 | 0 | 1 | 0 | 0 | 0 | 0 |
| *Chitinispirillum alkaliphilum* | 1 | 1 | 0 | 0 | 0 | 0 | 0 | 0 | 0 | 0 | 0 | 0 | 0 | 0 | 0 | 0 | 0 | 0 |
| *Chlamydia trachomatis* | 0 | 0 | 0 | 0 | 0 | 0 | 0 | 0 | 0 | 0 | 0 | 0 | 0 | 0 | 0 | 0 | 0 | 0 |
| *Chromobacterium* sp. ATCC 53434 | 1 | 1 | 1 | 0 | 0 | 0 | 0 | 0 | 0 | 0 | 0 | 0 | 0 | 0 | 0 | 0 | 1 | 0 |
| *Chthoniobacter flavus* | 0 | 0 | 1 | 0 | 0 | 0 | 0 | 0 | 0 | 0 | 0 | 0 | 0 | 0 | 0 | 0 | 0 | 0 |
| *Clostridioides difficile* | 0 | 0 | 0 | 0 | 0 | 0 | 0 | 0 | 0 | 0 | 0 | 0 | 0 | 0 | 2 | 1 | 0 | 0 |
| *Clostridium aurantibutyricum* | 1 | 2 | 0 | 0 | 0 | 0 | 1 | 1 | 0 | 0 | 0 | 0 | 0 | 0 | 2 | 1 | 0 | 0 |
| *Clostridium bolteae* | 0 | 0 | 0 | 0 | 0 | 0 | 0 | 0 | 0 | 0 | 0 | 0 | 0 | 0 | 4 | 2 | 0 | 0 |
| *Clostridium botulinum* | 0 | 0 | 0 | 0 | 0 | 0 | 0 | 0 | 0 | 0 | 0 | 0 | 0 | 0 | 0 | 0 | 0 | 0 |
| *Clostridium cadaveris* | 0 | 0 | 0 | 0 | 0 | 0 | 0 | 0 | 0 | 0 | 0 | 0 | 0 | 0 | 2 | 1 | 0 | 0 |
| *Clostridium cellulosi* | 0 | 0 | 0 | 0 | 0 | 0 | 0 | 0 | 0 | 0 | 0 | 0 | 0 | 0 | 0 | 0 | 0 | 0 |
| *Clostridium leptum* | 0 | 0 | 0 | 0 | 0 | 0 | 0 | 0 | 0 | 0 | 0 | 0 | 0 | 0 | 0 | 0 | 0 | 0 |
| *Clostridium* sp. BL8 | 0 | 0 | 0 | 0 | 0 | 0 | 0 | 0 | 0 | 0 | 0 | 0 | 0 | 0 | 2 | 1 | 0 | 0 |
| *Comamonas kerstersii* | 1 | 0 | 1 | 0 | 0 | 0 | 0 | 0 | 1 | 1 | 0 | 0 | 2 | 0 | 0 | 0 | 1 | 0 |
| *Comamonas testosteroni* | 1 | 0 | 1 | 0 | 0 | 0 | 0 | 1 | 3 | 5 | 0 | 0 | 1 | 0 | 0 | 0 | 1 | 0 |
| *Crenothrix polyspora* | 2 | 3 | 1 | 0 | 0 | 0 | 0 | 0 | 1 | 1 | 0 | 0 | 0 | 1 | 0 | 0 | 1 | 1 |
| *Cyanobium gracile* | 0 | 2 | 0 | 1 | 1 | 0 | 0 | 0 | 0 | 0 | 0 | 0 | 0 | 1 | 0 | 0 | 0 | 0 |
| *Delftia tsuruhatensis* | 1 | 1 | 1 | 0 | 0 | 0 | 0 | 0 | 0 | 0 | 0 | 0 | 0 | 1 | 0 | 0 | 1 | 0 |
| *Desulfomonile tiedjei* | 0 | 0 | 0 | 1 | 0 | 2 | 1 | 1 | 0 | 0 | 0 | 0 | 0 | 0 | 0 | 0 | 0 | 0 |
| *Desulfonauticus* sp. 38_4375 | 0 | 1 | 0 | 2 | 0 | 2 | 1 | 1 | 0 | 0 | 0 | 0 | 0 | 0 | 0 | 0 | 0 | 0 |
| *Desulfovibrio putealis* | 0 | 1 | 0 | 1 | 0 | 2 | 2 | 2 | 0 | 0 | 0 | 0 | 0 | 1 | 0 | 0 | 0 | 0 |
| *Dethiobacter alkaliphilus* | 1 | 1 | 0 | 0 | 0 | 0 | 1 | 1 | 0 | 0 | 0 | 0 | 0 | 0 | 0 | 0 | 0 | 0 |
| *Dictyobacter aurantiacus* | 0 | 0 | 1 | 1 | 1 | 0 | 0 | 0 | 0 | 0 | 0 | 0 | 0 | 0 | 0 | 0 | 0 | 0 |
| *Dinghuibacter silviterrae* | 1 | 1 | 0 | 0 | 0 | 0 | 0 | 0 | 0 | 0 | 0 | 0 | 0 | 1 | 0 | 0 | 0 | 0 |
| *Diplorickettsia massiliensis* | 0 | 0 | 0 | 0 | 0 | 0 | 0 | 0 | 0 | 0 | 0 | 0 | 0 | 0 | 0 | 0 | 0 | 0 |
| *Dorea longicatena* | 0 | 0 | 0 | 0 | 0 | 0 | 0 | 0 | 0 | 0 | 0 | 0 | 0 | 0 | 0 | 0 | 0 | 0 |
| *Duganella ginsengisoli* | 1 | 1 | 1 | 0 | 0 | 0 | 1 | 1 | 3 | 4 | 0 | 0 | 0 | 0 | 0 | 0 | 1 | 0 |
| *Dyella japonica* | 1 | 2 | 2 | 1 | 0 | 0 | 0 | 0 | 0 | 0 | 0 | 0 | 0 | 0 | 0 | 0 | 0 | 0 |
| *Dyella marensis* | 1 | 2 | 2 | 1 | 0 | 0 | 0 | 0 | 0 | 0 | 0 | 0 | 0 | 0 | 0 | 0 | 0 | 0 |
| *Elizabethkingia anophelis* | 1 | 1 | 2 | 0 | 0 | 0 | 0 | 0 | 0 | 0 | 0 | 0 | 0 | 0 | 0 | 0 | 0 | 0 |
| *Endomicrobium proavitum* | 0 | 0 | 0 | 0 | 0 | 0 | 0 | 0 | 0 | 0 | 0 | 0 | 0 | 0 | 0 | 0 | 0 | 0 |
| *Enterobacter cancerogenus* | 1 | 2 | 2 | 0 | 0 | 0 | 0 | 0 | 0 | 0 | 0 | 0 | 0 | 0 | 0 | 0 | 2 | 1 |
| *Enterococcus faecalis* | 0 | 0 | 0 | 0 | 0 | 0 | 0 | 0 | 0 | 0 | 0 | 0 | 0 | 0 | 0 | 0 | 1 | 0 |
| *Erythrobacter luteus* | 1 | 2 | 1 | 0 | 0 | 0 | 0 | 0 | 0 | 0 | 0 | 0 | 0 | 0 | 0 | 0 | 0 | 0 |
| *Escherichia coli* | 1 | 2 | 2 | 0 | 0 | 0 | 0 | 0 | 0 | 0 | 0 | 0 | 0 | 0 | 0 | 0 | 2 | 1 |
| *Fimbriimonas ginsengisoli* | 2 | 1 | 0 | 0 | 1 | 0 | 0 | 0 | 0 | 0 | 0 | 0 | 0 | 0 | 0 | 0 | 0 | 0 |
| *Flavisolibacter tropicus* | 1 | 1 | 0 | 0 | 0 | 0 | 0 | 0 | 0 | 0 | 0 | 0 | 0 | 0 | 0 | 0 | 0 | 0 |
| *Fonticella tunisiensis* | 0 | 0 | 0 | 0 | 0 | 0 | 0 | 0 | 0 | 0 | 0 | 0 | 0 | 0 | 2 | 1 | 0 | 0 |
| *Gaiella occulta* | 1 | 2 | 0 | 0 | 0 | 0 | 0 | 0 | 0 | 0 | 0 | 0 | 0 | 0 | 0 | 0 | 0 | 0 |
| *Gardnerella vaginalis* | 0 | 0 | 0 | 0 | 0 | 0 | 0 | 0 | 0 | 0 | 0 | 0 | 0 | 0 | 0 | 0 | 0 | 0 |
| *Geminisphaera colitermitum* | 1 | 1 | 1 | 0 | 0 | 0 | 0 | 0 | 0 | 0 | 0 | 0 | 0 | 0 | 0 | 0 | 0 | 0 |
| *Gemmata massiliana* | 1 | 2 | 0 | 0 | 0 | 0 | 0 | 0 | 0 | 0 | 0 | 0 | 0 | 0 | 0 | 0 | 0 | 0 |
| *Gemmatimonas phototrophica* | 1 | 1 | 0 | 0 | 0 | 0 | 0 | 0 | 0 | 4 | 0 | 0 | 0 | 1 | 0 | 0 | 0 | 0 |
| *Geobacter pelophilus* | 1 | 2 | 0 | 0 | 0 | 0 | 0 | 0 | 0 | 0 | 0 | 0 | 0 | 0 | 0 | 0 | 0 | 0 |
| *Haliangium ochraceum* | 1 | 1 | 0 | 0 | 0 | 0 | 0 | 0 | 0 | 0 | 0 | 0 | 0 | 1 | 0 | 0 | 0 | 0 |
| *Klebsiella oxytoca* | 1 | 2 | 2 | 0 | 0 | 0 | 0 | 0 | 0 | 0 | 0 | 0 | 0 | 0 | 0 | 0 | 2 | 1 |
| *Klebsiella pneumoniae* | 1 | 2 | 2 | 0 | 0 | 0 | 0 | 0 | 0 | 0 | 0 | 0 | 0 | 0 | 0 | 0 | 2 | 1 |
| *Ktedonobacter racemifer* | 0 | 0 | 1 | 1 | 1 | 0 | 0 | 0 | 0 | 0 | 0 | 0 | 2 | 0 | 0 | 0 | 0 | 0 |
| *Lactococcus garvieae* | 0 | 0 | 0 | 0 | 0 | 0 | 0 | 0 | 0 | 0 | 0 | 0 | 0 | 0 | 0 | 0 | 0 | 0 |
| *Lactococcus lactis* | 0 | 0 | 0 | 0 | 0 | 0 | 0 | 0 | 0 | 0 | 0 | 0 | 0 | 0 | 0 | 0 | 0 | 0 |
| *Legionella pneumophila* | 0 | 0 | 0 | 0 | 0 | 0 | 0 | 0 | 0 | 0 | 0 | 0 | 0 | 0 | 0 | 0 | 0 | 0 |
| *Lentimicrobium saccharophilum* | 1 | 1 | 0 | 0 | 0 | 0 | 0 | 0 | 0 | 0 | 0 | 0 | 0 | 1 | 0 | 0 | 0 | 0 |
| *Limisphaera ngatamarikiensis* | 1 | 0 | 1 | 0 | 1 | 0 | 0 | 0 | 0 | 0 | 0 | 0 | 0 | 1 | 0 | 0 | 0 | 0 |
| *Listeria monocytogenes* | 0 | 0 | 0 | 0 | 0 | 0 | 0 | 0 | 0 | 0 | 0 | 0 | 0 | 0 | 0 | 0 | 0 | 0 |
| *Listeria seeligeri* | 0 | 0 | 0 | 0 | 0 | 0 | 0 | 0 | 0 | 0 | 0 | 0 | 0 | 0 | 0 | 0 | 0 | 0 |
| *Litorilinea aerophila* | 1 | 2 | 0 | 0 | 0 | 0 | 0 | 0 | 0 | 0 | 0 | 0 | 0 | 1 | 0 | 0 | 0 | 0 |
| *Longilinea arvoryzae* | 0 | 0 | 0 | 0 | 0 | 0 | 0 | 0 | 0 | 0 | 0 | 0 | 0 | 1 | 0 | 0 | 0 | 0 |
| *Luteitalea pratensis* | 1 | 1 | 1 | 0 | 1 | 0 | 0 | 0 | 0 | 0 | 0 | 0 | 0 | 0 | 0 | 0 | 0 | 0 |
| *Magnetospirillum* sp. XM-1 | 1 | 2 | 0 | 1 | 0 | 2 | 0 | 0 | 2 | 2 | 0 | 0 | 3 | 1 | 0 | 0 | 2 | 1 |
| *Microcystis aeruginosa* | 0 | 1 | 1 | 1 | 1 | 0 | 0 | 0 | 0 | 0 | 0 | 0 | 0 | 0 | 0 | 0 | 0 | 0 |
| *Microvirga flocculans* | 0 | 0 | 0 | 0 | 0 | 0 | 0 | 0 | 0 | 0 | 0 | 0 | 0 | 0 | 0 | 0 | 1 | 0 |
| *Minicystis rosea* | 1 | 2 | 0 | 1 | 0 | 0 | 0 | 0 | 0 | 0 | 0 | 0 | 0 | 0 | 0 | 0 | 0 | 0 |
| *Moraxella osloensis* | 0 | 0 | 1 | 1 | 0 | 0 | 0 | 0 | 0 | 0 | 0 | 0 | 0 | 0 | 0 | 0 | 2 | 0 |
| *Morganella morganii* | 0 | 0 | 3 | 0 | 0 | 0 | 0 | 0 | 0 | 0 | 0 | 0 | 0 | 1 | 0 | 0 | 2 | 1 |
| *Mycobacteroides abscessus* | 3 | 3 | 3 | 0 | 1 | 0 | 0 | 0 | 0 | 0 | 0 | 0 | 0 | 1 | 0 | 0 | 0 | 0 |
| *Nitrospira japonica* | 1 | 1 | 0 | 1 | 0 | 0 | 0 | 0 | 0 | 2 | 0 | 0 | 0 | 0 | 0 | 0 | 1 | 0 |
| *Novimethylophilus kurashikiensis* | 1 | 1 | 1 | 0 | 0 | 0 | 0 | 0 | 2 | 1 | 0 | 0 | 1 | 1 | 0 | 0 | 1 | 0 |
| *Novosphingobium rosa* | 1 | 3 | 3 | 0 | 0 | 0 | 0 | 0 | 1 | 0 | 0 | 0 | 0 | 1 | 0 | 0 | 0 | 0 |
| *Opitutus terrae* | 2 | 0 | 2 | 0 | 0 | 0 | 0 | 0 | 0 | 0 | 0 | 0 | 0 | 0 | 0 | 0 | 0 | 0 |
| *Oscillochloris trichoides* | 0 | 0 | 0 | 0 | 0 | 0 | 0 | 0 | 0 | 0 | 0 | 0 | 0 | 1 | 0 | 0 | 0 | 0 |
| *Paenarthrobacter nicotinovorans* | 1 | 1 | 0 | 0 | 1 | 0 | 0 | 0 | 0 | 0 | 0 | 0 | 0 | 1 | 0 | 0 | 0 | 0 |
| *Paenibacillus herberti* | 0 | 1 | 0 | 1 | 0 | 0 | 0 | 0 | 0 | 0 | 0 | 0 | 0 | 0 | 0 | 0 | 0 | 0 |
| *Paeniclostridium sordellii* | 0 | 0 | 0 | 0 | 0 | 0 | 0 | 0 | 0 | 0 | 0 | 0 | 0 | 0 | 2 | 1 | 0 | 0 |
| *Paludisphaera borealis* | 1 | 2 | 1 | 1 | 1 | 0 | 0 | 0 | 0 | 0 | 0 | 0 | 0 | 0 | 0 | 0 | 0 | 0 |
| *Pantoea ananatis* | 1 | 2 | 3 | 0 | 0 | 0 | 0 | 0 | 0 | 0 | 0 | 0 | 0 | 1 | 0 | 0 | 2 | 1 |
| *Paraburkholderia nodosa* | 1 | 1 | 2 | 0 | 0 | 0 | 1 | 0 | 0 | 0 | 0 | 0 | 1 | 1 | 0 | 0 | 1 | 0 |
| *Paraburkholderia susongensis* | 1 | 1 | 2 | 0 | 0 | 0 | 0 | 0 | 0 | 2 | 0 | 0 | 1 | 0 | 0 | 0 | 1 | 0 |
| *Paracoccus denitrificans* | 1 | 3 | 1 | 0 | 0 | 0 | 0 | 1 | 3 | 4 | 0 | 0 | 0 | 1 | 0 | 0 | 1 | 0 |
| *Paracoccus yeei* | 0 | 1 | 1 | 1 | 0 | 0 | 0 | 0 | 0 | 0 | 0 | 0 | 0 | 1 | 0 | 0 | 1 | 0 |
| *Parafilimonas terrae* | 0 | 2 | 2 | 1 | 0 | 0 | 0 | 0 | 0 | 0 | 0 | 0 | 0 | 0 | 0 | 0 | 0 | 0 |
| *Pararhodospirillum photometricum* | 2 | 3 | 1 | 0 | 0 | 0 | 0 | 0 | 0 | 0 | 0 | 0 | 0 | 1 | 0 | 0 | 1 | 0 |
| *Parvimonas micra* | 0 | 0 | 0 | 0 | 0 | 0 | 0 | 0 | 0 | 0 | 0 | 0 | 0 | 0 | 0 | 0 | 0 | 0 |
| *Petrimonas mucosa* | 0 | 0 | 0 | 0 | 0 | 0 | 0 | 0 | 0 | 0 | 0 | 0 | 0 | 0 | 0 | 0 | 0 | 0 |
| *Pirellula staleyi* | 1 | 2 | 1 | 0 | 1 | 0 | 0 | 0 | 0 | 0 | 0 | 0 | 0 | 1 | 0 | 0 | 0 | 0 |
| *Proteiniphilum saccharofermentans* | 0 | 0 | 0 | 0 | 0 | 0 | 0 | 0 | 0 | 0 | 0 | 0 | 0 | 0 | 0 | 0 | 0 | 0 |
| *Pseudoclostridium thermosuccinogenes* | 0 | 0 | 0 | 0 | 0 | 0 | 0 | 0 | 0 | 0 | 0 | 0 | 0 | 0 | 0 | 0 | 0 | 0 |
| *Pseudoduganella eburnea* | 2 | 2 | 1 | 0 | 0 | 0 | 0 | 0 | 0 | 0 | 0 | 0 | 1 | 0 | 0 | 0 | 1 | 0 |
| *Pseudoflavonifractor capillosus* | 0 | 0 | 0 | 0 | 0 | 0 | 0 | 0 | 0 | 0 | 0 | 0 | 0 | 0 | 0 | 0 | 0 | 0 |
| *Pseudomonas flexibilis* | 1 | 2 | 2 | 0 | 0 | 0 | 0 | 0 | 0 | 0 | 0 | 0 | 1 | 1 | 0 | 0 | 3 | 1 |
| *Pseudomonas lini* | 1 | 2 | 3 | 0 | 0 | 0 | 1 | 0 | 1 | 0 | 0 | 0 | 0 | 1 | 0 | 0 | 3 | 1 |
| *Reyranella soli* | 2 | 3 | 1 | 0 | 0 | 0 | 0 | 1 | 2 | 1 | 0 | 0 | 3 | 0 | 0 | 0 | 1 | 0 |
| *Rhodobacter* sp. LPB0142 | 0 | 1 | 1 | 1 | 0 | 0 | 0 | 0 | 0 | 0 | 0 | 0 | 1 | 3 | 0 | 0 | 1 | 0 |
| *Rhodopila globiformis* | 2 | 2 | 2 | 0 | 0 | 0 | 0 | 0 | 1 | 2 | 0 | 0 | 2 | 3 | 0 | 0 | 2 | 0 |
| *Rhodovulum sulfidophilum* | 0 | 2 | 1 | 1 | 0 | 0 | 0 | 1 | 2 | 4 | 0 | 0 | 1 | 1 | 0 | 0 | 1 | 0 |
| *Rickettsia prowazekii* | 0 | 0 | 0 | 0 | 0 | 0 | 0 | 0 | 0 | 0 | 0 | 0 | 0 | 0 | 0 | 0 | 0 | 0 |
| *Roseiarcus fermentans* | 1 | 2 | 1 | 0 | 0 | 0 | 0 | 0 | 0 | 0 | 0 | 0 | 0 | 0 | 0 | 0 | 1 | 0 |
| *Rudaea cellulosilytica* | 1 | 3 | 2 | 0 | 0 | 0 | 0 | 0 | 0 | 0 | 0 | 0 | 0 | 0 | 0 | 0 | 0 | 0 |
| *Rugosibacter aromaticivorans* | 1 | 0 | 1 | 0 | 0 | 0 | 0 | 0 | 0 | 0 | 0 | 0 | 0 | 0 | 0 | 0 | 1 | 0 |
| *Ruminiclostridium cellobioparum* | 1 | 1 | 0 | 0 | 0 | 0 | 0 | 0 | 0 | 0 | 0 | 0 | 0 | 0 | 0 | 0 | 0 | 0 |
| *Ruminococcus gnavus* | 0 | 0 | 0 | 0 | 0 | 0 | 0 | 0 | 0 | 0 | 0 | 0 | 0 | 0 | 0 | 0 | 0 | 0 |
| *Salmonella enterica* | 1 | 2 | 2 | 0 | 0 | 0 | 0 | 0 | 0 | 0 | 0 | 0 | 0 | 0 | 2 | 1 | 2 | 1 |
| *Sedimentibacter saalensis* | 0 | 0 | 0 | 0 | 0 | 0 | 0 | 0 | 0 | 0 | 0 | 0 | 0 | 0 | 0 | 0 | 0 | 0 |
| *Shewanella benthica* | 1 | 2 | 2 | 0 | 0 | 0 | 0 | 0 | 0 | 0 | 0 | 0 | 0 | 0 | 0 | 0 | 2 | 1 |
| *Singulisphaera acidiphila* | 1 | 2 | 0 | 1 | 1 | 0 | 0 | 0 | 0 | 0 | 0 | 0 | 0 | 0 | 0 | 0 | 0 | 0 |
| *Sinosporangium album* | 1 | 1 | 0 | 0 | 1 | 0 | 0 | 0 | 0 | 0 | 0 | 0 | 0 | 0 | 0 | 0 | 0 | 0 |
| *Solitalea koreensis* | 1 | 1 | 0 | 0 | 1 | 0 | 0 | 0 | 0 | 0 | 0 | 0 | 0 | 0 | 0 | 0 | 0 | 0 |
| *Sorangium cellulosum* | 0 | 1 | 0 | 2 | 0 | 0 | 0 | 0 | 0 | 0 | 0 | 0 | 0 | 0 | 0 | 0 | 0 | 0 |
| *Sphingorhabdus contaminans* | 1 | 3 | 1 | 0 | 0 | 0 | 0 | 0 | 0 | 0 | 0 | 0 | 0 | 0 | 0 | 0 | 0 | 0 |
| *Sporomusa malonica* | 1 | 0 | 0 | 0 | 0 | 0 | 1 | 1 | 0 | 0 | 0 | 0 | 0 | 0 | 2 | 1 | 0 | 0 |
| *Staphylococcus aureus* | 0 | 0 | 1 | 0 | 0 | 0 | 0 | 0 | 0 | 0 | 0 | 0 | 0 | 1 | 0 | 0 | 0 | 0 |
| *Stenotrophomonas maltophilia* | 0 | 0 | 0 | 0 | 0 | 0 | 0 | 0 | 0 | 0 | 0 | 0 | 0 | 0 | 0 | 0 | 0 | 0 |
| *Streptococcus pneumoniae* | 0 | 0 | 0 | 0 | 0 | 0 | 0 | 0 | 0 | 0 | 0 | 0 | 0 | 0 | 0 | 0 | 0 | 0 |
| *Streptococcus ruminantium* | 0 | 0 | 0 | 0 | 0 | 0 | 0 | 0 | 0 | 0 | 0 | 0 | 0 | 0 | 0 | 0 | 0 | 0 |
| *Streptococcus thermophilus* | 0 | 0 | 0 | 0 | 0 | 0 | 0 | 0 | 0 | 0 | 0 | 0 | 0 | 0 | 0 | 0 | 0 | 0 |
| *Streptomyces cinnamoneus* | 1 | 1 | 0 | 0 | 1 | 0 | 0 | 0 | 0 | 0 | 0 | 0 | 0 | 0 | 0 | 0 | 0 | 0 |
| *Streptomyces himastatinicus* | 1 | 1 | 1 | 0 | 1 | 0 | 0 | 0 | 0 | 0 | 0 | 0 | 0 | 0 | 0 | 0 | 0 | 0 |
| *Sutterella* sp. CAG:521 | 0 | 0 | 0 | 0 | 0 | 0 | 0 | 0 | 0 | 0 | 0 | 0 | 0 | 0 | 0 | 0 | 0 | 0 |
| *Synechococcus* sp. WH 8109 | 0 | 2 | 0 | 1 | 1 | 0 | 0 | 0 | 0 | 0 | 0 | 0 | 0 | 0 | 0 | 0 | 0 | 0 |
| *Syntrophobacter* sp. SbD1 | 0 | 0 | 0 | 1 | 0 | 2 | 0 | 1 | 0 | 0 | 0 | 0 | 0 | 0 | 0 | 0 | 0 | 0 |
| *Syntrophus aciditrophicus* | 0 | 0 | 0 | 0 | 0 | 0 | 0 | 0 | 0 | 0 | 0 | 0 | 0 | 0 | 0 | 0 | 0 | 0 |
| *Taylorella asinigenitalis* | 0 | 0 | 0 | 0 | 0 | 0 | 0 | 0 | 0 | 0 | 0 | 0 | 0 | 0 | 0 | 0 | 0 | 0 |
| *Telmatospirillum siberiense* | 1 | 2 | 0 | 0 | 0 | 0 | 0 | 1 | 3 | 1 | 0 | 0 | 2 | 0 | 0 | 0 | 2 | 1 |
| *Terrimicrobium sacchariphilum* | 1 | 2 | 2 | 0 | 0 | 0 | 0 | 0 | 0 | 0 | 0 | 0 | 0 | 0 | 0 | 0 | 0 | 0 |
| *Thermotalea metallivorans* | 0 | 0 | 0 | 0 | 0 | 0 | 0 | 0 | 0 | 0 | 0 | 0 | 0 | 0 | 2 | 1 | 0 | 0 |
| *Thermus thermophilus* | 0 | 1 | 0 | 1 | 0 | 0 | 0 | 2 | 3 | 4 | 0 | 0 | 0 | 0 | 0 | 0 | 0 | 0 |
| *Thiobacillus thioparus* | 0 | 0 | 0 | 2 | 0 | 4 | 2 | 4 | 3 | 4 | 0 | 0 | 0 | 5 | 0 | 0 | 3 | 1 |
| *Tissierella praeacuta* | 0 | 0 | 0 | 0 | 0 | 0 | 0 | 0 | 0 | 0 | 0 | 0 | 0 | 0 | 2 | 1 | 0 | 0 |
| *Vibrio parahaemolyticus* | 1 | 2 | 2 | 0 | 0 | 0 | 0 | 0 | 0 | 0 | 0 | 0 | 1 | 0 | 0 | 0 | 2 | 1 |
| *Virgibacillus profundi* | 0 | 0 | 0 | 1 | 0 | 0 | 0 | 0 | 0 | 0 | 0 | 0 | 0 | 0 | 0 | 0 | 1 | 0 |
| *Vulgatibacter incomptus* | 0 | 0 | 0 | 0 | 0 | 0 | 0 | 0 | 0 | 0 | 0 | 0 | 0 | 0 | 0 | 0 | 0 | 0 |
| *Xanthomonas citri* | 1 | 2 | 3 | 0 | 0 | 0 | 0 | 0 | 0 | 0 | 0 | 0 | 0 | 0 | 0 | 0 | 0 | 0 |
| *Yangia pacifica* | 1 | 2 | 1 | 0 | 0 | 0 | 0 | 1 | 3 | 4 | 0 | 0 | 1 | 0 | 0 | 0 | 1 | 0 |
| *Zavarzinella formosa* | 1 | 2 | 1 | 0 | 1 | 0 | 0 | 0 | 0 | 0 | 0 | 0 | 0 | 0 | 0 | 0 | 0 | 0 |

**Supplementary Table S10: The functional profiles of the enriched soil bacterial species related to utilisation of carbohydrates and aromatic compounds.** Only the enriched species with available genomes were displayed.

| **Species** | **Carbohydrate utilisation** | | | | | | | | | | **Aromatic compound utilisation** | | | | | | | |
| --- | --- | --- | --- | --- | --- | --- | --- | --- | --- | --- | --- | --- | --- | --- | --- | --- | --- | --- |
|  | **malZ** | **glvA** | **galA** | **lacA** | **lacZ** | **MAN2C1** | **sga1** | **cbh1** | **cbhA** | **pel** | **ethA** | **nagH** | **nagG** | **dctA** | **dctP** | **phdF** | **nahD** | **dctM** |
| *Acetobacter pomorum* | 0 | 0 | 0 | 0 | 0 | 0 | 0 | 0 | 0 | 0 | 0 | 0 | 0 | 1 | 0 | 0 | 0 | 0 |
| *Acetobacteroides hydrogenigenes* | 0 | 0 | 2 | 1 | 5 | 0 | 0 | 0 | 0 | 0 | 0 | 0 | 0 | 0 | 0 | 0 | 0 | 0 |
| *Acidisphaera rubrifaciens* | 0 | 0 | 0 | 0 | 0 | 0 | 1 | 0 | 0 | 0 | 0 | 0 | 0 | 0 | 0 | 0 | 0 | 0 |
| *Acinetobacter baumannii* | 0 | 0 | 0 | 0 | 0 | 0 | 0 | 0 | 0 | 0 | 0 | 0 | 0 | 3 | 0 | 0 | 0 | 0 |
| *Alkanindiges illinoisensis* | 0 | 0 | 0 | 0 | 0 | 0 | 0 | 0 | 0 | 0 | 0 | 0 | 0 | 0 | 0 | 0 | 0 | 0 |
| *Anaerocolumna aminovalerica* | 1 | 1 | 1 | 0 | 1 | 2 | 0 | 0 | 0 | 0 | 0 | 0 | 0 | 0 | 0 | 0 | 0 | 0 |
| *Anaerolinea thermophila* | 2 | 0 | 1 | 2 | 1 | 1 | 0 | 0 | 0 | 0 | 0 | 0 | 0 | 0 | 0 | 0 | 0 | 0 |
| *Anaeromyxobacter dehalogenans* | 0 | 0 | 0 | 0 | 0 | 0 | 0 | 0 | 0 | 0 | 0 | 0 | 0 | 1 | 0 | 0 | 0 | 0 |
| *Aquicella siphonis* | 0 | 0 | 0 | 0 | 0 | 0 | 1 | 0 | 0 | 0 | 0 | 0 | 0 | 0 | 0 | 0 | 0 | 0 |
| *Aquisphaera giovannonii* | 0 | 0 | 1 | 1 | 1 | 1 | 1 | 0 | 0 | 0 | 0 | 0 | 0 | 0 | 0 | 0 | 0 | 0 |
| *Azospirillum* sp. CAG:260 | 0 | 0 | 0 | 0 | 0 | 0 | 0 | 0 | 0 | 0 | 0 | 0 | 0 | 0 | 0 | 0 | 0 | 0 |
| *Bacillus cereus* | 2 | 0 | 0 | 0 | 0 | 0 | 0 | 0 | 0 | 0 | 0 | 0 | 0 | 0 | 0 | 0 | 0 | 0 |
| *Bacillus thuringiensis* | 1 | 0 | 0 | 0 | 0 | 0 | 0 | 0 | 0 | 0 | 0 | 0 | 0 | 0 | 0 | 0 | 0 | 0 |
| *Bradyrhizobium erythrophlei* | 0 | 0 | 0 | 0 | 0 | 0 | 0 | 0 | 0 | 0 | 1 | 0 | 0 | 4 | 0 | 0 | 0 | 2 |
| *Brevibacillus fluminis* | 0 | 0 | 0 | 0 | 1 | 0 | 0 | 0 | 0 | 0 | 0 | 0 | 0 | 1 | 0 | 0 | 0 | 0 |
| *Brucella suis* | 0 | 0 | 0 | 0 | 0 | 0 | 0 | 0 | 0 | 0 | 0 | 0 | 0 | 0 | 0 | 0 | 0 | 0 |
| *Burkholderia cenocepacia* | 1 | 0 | 1 | 2 | 0 | 0 | 0 | 0 | 0 | 0 | 1 | 0 | 0 | 3 | 0 | 0 | 0 | 0 |
| *Burkholderia cepacia* | 1 | 0 | 0 | 2 | 0 | 0 | 0 | 0 | 0 | 0 | 1 | 1 | 1 | 3 | 0 | 0 | 0 | 0 |
| *Burkholderia pseudomallei* | 0 | 0 | 1 | 0 | 0 | 0 | 1 | 0 | 0 | 0 | 1 | 0 | 0 | 2 | 0 | 0 | 0 | 0 |
| *Burkholderia pseudomultivorans* | 1 | 0 | 0 | 1 | 0 | 0 | 0 | 0 | 0 | 0 | 1 | 0 | 0 | 2 | 0 | 0 | 0 | 1 |
| *Bythopirellula goksoyri* | 0 | 0 | 0 | 0 | 4 | 1 | 1 | 0 | 0 | 0 | 0 | 0 | 0 | 0 | 0 | 0 | 0 | 0 |
| *Candidatus* Giovannonibacteria bacterium RIFCSPHIGHO2_12_FULL_44_42 | 0 | 0 | 0 | 0 | 0 | 0 | 0 | 0 | 0 | 0 | 0 | 0 | 0 | 0 | 0 | 0 | 0 | 0 |
| *Candidatus Koribacter versatilis* | 4 | 0 | 4 | 1 | 1 | 1 | 1 | 0 | 0 | 0 | 0 | 0 | 0 | 0 | 0 | 0 | 0 | 0 |
| *Candidatus* Levybacteria bacterium GW2011_GWB1_41_21 | 0 | 0 | 0 | 0 | 0 | 0 | 0 | 0 | 0 | 0 | 0 | 0 | 0 | 0 | 0 | 0 | 0 | 0 |
| *Candidatus* Liptonbacteria bacterium CG11_big_fil_rev_8_21_14_0_20_35_14 | 0 | 0 | 0 | 0 | 0 | 0 | 0 | 0 | 0 | 0 | 0 | 0 | 0 | 0 | 0 | 0 | 0 | 0 |
| *Candidatus* Rokubacteria bacterium | 0 | 0 | 0 | 0 | 0 | 0 | 0 | 0 | 0 | 0 | 0 | 0 | 0 | 0 | 0 | 0 | 0 | 0 |
| *Candidatus Solibacter usitatus* | 0 | 0 | 7 | 1 | 4 | 0 | 0 | 0 | 0 | 0 | 0 | 0 | 0 | 1 | 0 | 0 | 0 | 0 |
| *Candidatus Sulfopaludibacter* sp. SbA4 | 1 | 0 | 4 | 1 | 1 | 0 | 0 | 0 | 0 | 0 | 0 | 0 | 0 | 0 | 0 | 0 | 0 | 0 |
| *Candidatus Sulfotelmatobacter kueseliae* | 1 | 0 | 8 | 2 | 2 | 1 | 1 | 0 | 0 | 0 | 0 | 0 | 0 | 0 | 0 | 0 | 0 | 0 |
| *Candidatus* Woesebacteria bacterium RBG_16_36_11 | 0 | 0 | 0 | 0 | 0 | 0 | 0 | 0 | 0 | 0 | 0 | 0 | 0 | 0 | 0 | 0 | 0 | 0 |
| *Candidatus* Zambryskibacteria bacterium CG10_big_fil_rev_8_21_14_0_10_42_12 | 0 | 0 | 0 | 0 | 0 | 0 | 0 | 0 | 0 | 0 | 0 | 0 | 0 | 0 | 0 | 0 | 0 | 0 |
| *Carboxydocella* sp. JDF658 | 1 | 0 | 0 | 0 | 0 | 0 | 1 | 0 | 0 | 0 | 0 | 0 | 0 | 0 | 0 | 0 | 0 | 1 |
| *Chitinispirillum alkaliphilum* | 0 | 0 | 0 | 0 | 0 | 0 | 0 | 0 | 0 | 0 | 0 | 0 | 0 | 0 | 0 | 0 | 0 | 0 |
| *Chlamydia trachomatis* | 0 | 0 | 0 | 0 | 0 | 0 | 0 | 0 | 0 | 0 | 0 | 0 | 0 | 0 | 0 | 0 | 0 | 0 |
| *Chromobacterium* sp. ATCC 53434 | 0 | 0 | 0 | 0 | 0 | 0 | 1 | 0 | 0 | 0 | 0 | 0 | 0 | 1 | 0 | 0 | 0 | 1 |
| *Chthoniobacter flavus* | 0 | 0 | 0 | 0 | 0 | 0 | 0 | 0 | 0 | 0 | 0 | 0 | 0 | 1 | 0 | 0 | 0 | 0 |
| *Clostridioides difficile* | 0 | 4 | 0 | 0 | 0 | 0 | 0 | 0 | 0 | 0 | 0 | 0 | 0 | 0 | 0 | 0 | 0 | 0 |
| *Clostridium aurantibutyricum* | 0 | 0 | 1 | 1 | 1 | 0 | 0 | 0 | 0 | 1 | 0 | 0 | 0 | 0 | 0 | 0 | 0 | 0 |
| *Clostridium bolteae* | 1 | 0 | 3 | 1 | 1 | 1 | 0 | 0 | 0 | 0 | 0 | 0 | 0 | 0 | 3 | 0 | 0 | 7 |
| *Clostridium botulinum* | 0 | 3 | 0 | 0 | 0 | 0 | 0 | 0 | 0 | 0 | 0 | 0 | 0 | 0 | 0 | 0 | 0 | 0 |
| *Clostridium cadaveris* | 0 | 1 | 0 | 0 | 1 | 0 | 0 | 0 | 0 | 0 | 0 | 0 | 0 | 0 | 0 | 0 | 0 | 1 |
| *Clostridium cellulosi* | 0 | 0 | 0 | 1 | 1 | 0 | 0 | 0 | 0 | 0 | 0 | 0 | 0 | 0 | 0 | 0 | 0 | 0 |
| *Clostridium leptum* | 1 | 0 | 3 | 1 | 1 | 1 | 0 | 0 | 0 | 0 | 0 | 0 | 0 | 0 | 0 | 0 | 0 | 0 |
| *Clostridium* sp. BL8 | 1 | 0 | 0 | 0 | 0 | 0 | 0 | 0 | 0 | 0 | 0 | 0 | 0 | 0 | 0 | 0 | 0 | 0 |
| *Comamonas kerstersii* | 0 | 0 | 0 | 0 | 0 | 0 | 0 | 0 | 0 | 0 | 0 | 0 | 0 | 1 | 1 | 0 | 0 | 0 |
| *Comamonas testosteroni* | 0 | 0 | 0 | 0 | 0 | 0 | 0 | 0 | 0 | 0 | 0 | 0 | 0 | 2 | 2 | 0 | 0 | 0 |
| *Crenothrix polyspora* | 0 | 0 | 0 | 0 | 0 | 0 | 0 | 0 | 0 | 0 | 0 | 0 | 0 | 1 | 0 | 0 | 0 | 0 |
| *Cyanobium gracile* | 0 | 0 | 0 | 0 | 0 | 0 | 0 | 0 | 0 | 0 | 0 | 0 | 0 | 0 | 0 | 0 | 0 | 0 |
| *Delftia tsuruhatensis* | 0 | 0 | 0 | 0 | 0 | 0 | 0 | 0 | 0 | 0 | 0 | 0 | 0 | 2 | 3 | 0 | 0 | 0 |
| *Desulfomonile tiedjei* | 0 | 0 | 0 | 0 | 0 | 0 | 1 | 0 | 0 | 0 | 0 | 0 | 0 | 0 | 0 | 0 | 0 | 1 |
| *Desulfonauticus* sp. 38_4375 | 0 | 0 | 0 | 0 | 0 | 0 | 0 | 0 | 0 | 0 | 0 | 0 | 0 | 0 | 0 | 0 | 0 | 0 |
| *Desulfovibrio putealis* | 0 | 0 | 0 | 0 | 0 | 0 | 0 | 0 | 0 | 0 | 0 | 0 | 0 | 1 | 0 | 0 | 0 | 0 |
| *Dethiobacter alkaliphilus* | 0 | 0 | 0 | 0 | 0 | 0 | 1 | 0 | 0 | 0 | 0 | 0 | 0 | 0 | 0 | 0 | 0 | 0 |
| *Dictyobacter aurantiacus* | 2 | 0 | 5 | 2 | 0 | 2 | 1 | 0 | 1 | 0 | 0 | 0 | 0 | 0 | 0 | 0 | 0 | 0 |
| *Dinghuibacter silviterrae* | 2 | 0 | 5 | 4 | 5 | 0 | 0 | 0 | 0 | 0 | 0 | 0 | 0 | 0 | 0 | 0 | 0 | 0 |
| *Diplorickettsia massiliensis* | 0 | 0 | 0 | 0 | 0 | 0 | 0 | 0 | 0 | 0 | 0 | 0 | 0 | 0 | 0 | 0 | 0 | 0 |
| *Dorea longicatena* | 0 | 0 | 1 | 0 | 3 | 0 | 0 | 0 | 0 | 0 | 0 | 0 | 0 | 0 | 0 | 0 | 0 | 0 |
| *Duganella ginsengisoli* | 0 | 0 | 0 | 0 | 0 | 0 | 0 | 0 | 0 | 0 | 0 | 0 | 0 | 1 | 0 | 0 | 0 | 1 |
| *Dyella japonica* | 0 | 0 | 2 | 1 | 0 | 0 | 0 | 0 | 0 | 0 | 0 | 0 | 0 | 0 | 0 | 0 | 0 | 0 |
| *Dyella marensis* | 1 | 0 | 3 | 0 | 0 | 0 | 0 | 0 | 0 | 0 | 0 | 0 | 0 | 1 | 0 | 0 | 0 | 0 |
| *Elizabethkingia anophelis* | 0 | 0 | 2 | 1 | 3 | 0 | 0 | 0 | 0 | 0 | 0 | 0 | 0 | 0 | 0 | 0 | 0 | 0 |
| *Endomicrobium proavitum* | 0 | 0 | 0 | 0 | 0 | 0 | 0 | 0 | 0 | 0 | 0 | 0 | 0 | 0 | 0 | 0 | 0 | 0 |
| *Enterobacter cancerogenus* | 1 | 1 | 0 | 0 | 1 | 0 | 0 | 0 | 0 | 0 | 0 | 0 | 0 | 1 | 0 | 0 | 0 | 1 |
| *Enterococcus faecalis* | 0 | 0 | 0 | 3 | 1 | 0 | 0 | 0 | 0 | 0 | 0 | 0 | 0 | 0 | 0 | 0 | 0 | 0 |
| *Erythrobacter luteus* | 1 | 0 | 1 | 1 | 1 | 0 | 0 | 0 | 0 | 0 | 0 | 0 | 0 | 1 | 0 | 0 | 0 | 0 |
| *Escherichia coli* | 0 | 0 | 0 | 0 | 2 | 0 | 0 | 0 | 0 | 0 | 0 | 0 | 0 | 1 | 1 | 0 | 0 | 0 |
| *Fimbriimonas ginsengisoli* | 1 | 0 | 5 | 2 | 4 | 4 | 0 | 0 | 0 | 0 | 0 | 0 | 0 | 0 | 0 | 0 | 0 | 0 |
| *Flavisolibacter tropicus* | 0 | 0 | 0 | 0 | 2 | 0 | 0 | 0 | 0 | 0 | 0 | 0 | 0 | 1 | 0 | 0 | 0 | 0 |
| *Fonticella tunisiensis* | 0 | 3 | 0 | 0 | 1 | 1 | 0 | 0 | 0 | 0 | 0 | 0 | 0 | 0 | 1 | 0 | 0 | 1 |
| *Gaiella occulta* | 0 | 0 | 0 | 0 | 0 | 0 | 0 | 0 | 0 | 0 | 0 | 0 | 0 | 0 | 0 | 0 | 0 | 0 |
| *Gardnerella vaginalis* | 1 | 0 | 0 | 0 | 2 | 1 | 0 | 0 | 0 | 0 | 0 | 0 | 0 | 0 | 0 | 0 | 0 | 0 |
| *Geminisphaera colitermitum* | 0 | 0 | 3 | 0 | 3 | 2 | 0 | 0 | 0 | 0 | 0 | 0 | 0 | 0 | 0 | 0 | 0 | 0 |
| *Gemmata massiliana* | 0 | 0 | 0 | 0 | 0 | 0 | 0 | 0 | 0 | 0 | 0 | 0 | 0 | 0 | 0 | 0 | 0 | 0 |
| *Gemmatimonas phototrophica* | 0 | 0 | 0 | 0 | 0 | 0 | 0 | 0 | 0 | 0 | 0 | 0 | 0 | 1 | 0 | 0 | 0 | 0 |
| *Geobacter pelophilus* | 0 | 0 | 0 | 0 | 0 | 0 | 0 | 0 | 0 | 0 | 0 | 0 | 0 | 1 | 0 | 0 | 0 | 1 |
| *Haliangium ochraceum* | 0 | 0 | 1 | 0 | 0 | 0 | 0 | 0 | 0 | 0 | 0 | 0 | 0 | 0 | 0 | 0 | 0 | 0 |
| *Klebsiella oxytoca* | 1 | 2 | 1 | 2 | 2 | 0 | 0 | 0 | 0 | 0 | 0 | 0 | 0 | 1 | 0 | 0 | 0 | 0 |
| *Klebsiella pneumoniae* | 1 | 2 | 1 | 1 | 1 | 0 | 0 | 0 | 0 | 0 | 0 | 0 | 0 | 1 | 0 | 0 | 0 | 0 |
| *Ktedonobacter racemifer* | 3 | 0 | 5 | 2 | 0 | 2 | 2 | 0 | 1 | 0 | 1 | 0 | 0 | 0 | 0 | 0 | 0 | 0 |
| *Lactococcus garvieae* | 1 | 0 | 0 | 0 | 0 | 0 | 0 | 0 | 0 | 0 | 0 | 0 | 0 | 0 | 0 | 0 | 0 | 0 |
| *Lactococcus lactis* | 0 | 0 | 1 | 0 | 1 | 0 | 0 | 0 | 0 | 0 | 0 | 0 | 0 | 0 | 0 | 0 | 0 | 0 |
| *Legionella pneumophila* | 0 | 0 | 0 | 0 | 0 | 0 | 1 | 0 | 0 | 0 | 0 | 0 | 0 | 1 | 0 | 0 | 0 | 0 |
| *Lentimicrobium saccharophilum* | 2 | 0 | 0 | 0 | 2 | 0 | 0 | 0 | 0 | 0 | 0 | 0 | 0 | 0 | 0 | 0 | 0 | 0 |
| *Limisphaera ngatamarikiensis* | 0 | 0 | 2 | 1 | 2 | 0 | 0 | 0 | 0 | 2 | 0 | 0 | 0 | 0 | 0 | 0 | 0 | 0 |
| *Listeria monocytogenes* | 1 | 0 | 0 | 0 | 0 | 1 | 0 | 0 | 0 | 0 | 0 | 0 | 0 | 0 | 0 | 0 | 0 | 0 |
| *Listeria seeligeri* | 1 | 1 | 0 | 0 | 0 | 1 | 0 | 0 | 0 | 0 | 0 | 0 | 0 | 0 | 0 | 0 | 0 | 0 |
| *Litorilinea aerophila* | 0 | 0 | 1 | 1 | 1 | 0 | 0 | 0 | 0 | 0 | 0 | 0 | 0 | 0 | 0 | 0 | 0 | 0 |
| *Longilinea arvoryzae* | 2 | 0 | 1 | 0 | 0 | 0 | 1 | 0 | 0 | 0 | 0 | 0 | 0 | 0 | 0 | 0 | 0 | 0 |
| *Luteitalea pratensis* | 0 | 0 | 0 | 0 | 1 | 0 | 0 | 0 | 0 | 1 | 0 | 0 | 0 | 1 | 0 | 0 | 0 | 0 |
| *Magnetospirillum* sp. XM-1 | 0 | 0 | 0 | 0 | 0 | 0 | 0 | 0 | 0 | 0 | 0 | 0 | 0 | 1 | 0 | 0 | 0 | 1 |
| *Microcystis aeruginosa* | 0 | 0 | 0 | 0 | 0 | 1 | 0 | 0 | 0 | 0 | 0 | 0 | 0 | 0 | 0 | 0 | 0 | 0 |
| *Microvirga flocculans* | 0 | 0 | 0 | 0 | 0 | 0 | 1 | 0 | 0 | 0 | 0 | 0 | 0 | 0 | 0 | 0 | 0 | 0 |
| *Minicystis rosea* | 2 | 0 | 0 | 0 | 0 | 0 | 0 | 0 | 0 | 0 | 1 | 0 | 0 | 1 | 0 | 0 | 1 | 0 |
| *Moraxella osloensis* | 0 | 0 | 0 | 0 | 0 | 0 | 0 | 0 | 0 | 0 | 0 | 0 | 0 | 0 | 0 | 0 | 0 | 0 |
| *Morganella morganii* | 0 | 0 | 0 | 0 | 1 | 0 | 0 | 0 | 0 | 0 | 0 | 0 | 0 | 0 | 0 | 0 | 0 | 0 |
| *Mycobacteroides abscessus* | 1 | 0 | 1 | 0 | 0 | 1 | 0 | 0 | 0 | 0 | 2 | 0 | 0 | 2 | 0 | 0 | 0 | 0 |
| *Nitrospira japonica* | 1 | 0 | 0 | 0 | 0 | 0 | 0 | 0 | 0 | 0 | 0 | 0 | 0 | 0 | 0 | 0 | 0 | 0 |
| *Novimethylophilus kurashikiensis* | 0 | 0 | 0 | 0 | 0 | 0 | 0 | 0 | 0 | 0 | 0 | 0 | 0 | 0 | 0 | 0 | 0 | 0 |
| *Novosphingobium rosa* | 2 | 0 | 1 | 3 | 1 | 0 | 0 | 0 | 0 | 0 | 0 | 0 | 0 | 1 | 0 | 0 | 0 | 0 |
| *Opitutus terrae* | 0 | 0 | 3 | 2 | 7 | 0 | 0 | 0 | 0 | 0 | 0 | 0 | 0 | 0 | 0 | 0 | 0 | 0 |
| *Oscillochloris trichoides* | 0 | 0 | 0 | 0 | 0 | 0 | 0 | 0 | 0 | 0 | 0 | 0 | 0 | 0 | 0 | 0 | 0 | 0 |
| *Paenarthrobacter nicotinovorans* | 2 | 0 | 2 | 5 | 1 | 1 | 0 | 0 | 0 | 2 | 0 | 0 | 0 | 2 | 0 | 0 | 0 | 0 |
| *Paenibacillus herberti* | 1 | 0 | 0 | 2 | 4 | 2 | 1 | 0 | 0 | 0 | 0 | 0 | 0 | 0 | 0 | 0 | 0 | 0 |
| *Paeniclostridium sordellii* | 0 | 0 | 0 | 1 | 1 | 0 | 0 | 0 | 0 | 0 | 0 | 0 | 0 | 0 | 0 | 0 | 0 | 0 |
| *Paludisphaera borealis* | 0 | 0 | 0 | 0 | 0 | 1 | 0 | 0 | 0 | 0 | 0 | 0 | 0 | 0 | 0 | 0 | 0 | 0 |
| *Pantoea ananatis* | 1 | 1 | 0 | 0 | 1 | 0 | 1 | 0 | 0 | 0 | 0 | 0 | 0 | 1 | 0 | 0 | 0 | 0 |
| *Paraburkholderia nodosa* | 0 | 0 | 0 | 2 | 0 | 0 | 0 | 0 | 0 | 0 | 0 | 0 | 0 | 5 | 0 | 0 | 0 | 0 |
| *Paraburkholderia susongensis* | 1 | 0 | 3 | 2 | 0 | 0 | 0 | 0 | 0 | 1 | 0 | 1 | 1 | 3 | 0 | 0 | 0 | 0 |
| *Paracoccus denitrificans* | 0 | 0 | 0 | 0 | 0 | 0 | 0 | 0 | 0 | 0 | 0 | 0 | 0 | 2 | 2 | 0 | 0 | 1 |
| *Paracoccus yeei* | 1 | 0 | 0 | 0 | 0 | 0 | 0 | 0 | 0 | 0 | 0 | 0 | 0 | 2 | 1 | 0 | 0 | 0 |
| *Parafilimonas terrae* | 0 | 0 | 4 | 0 | 5 | 0 | 1 | 0 | 0 | 0 | 0 | 0 | 0 | 1 | 0 | 0 | 0 | 0 |
| *Pararhodospirillum photometricum* | 0 | 0 | 0 | 0 | 0 | 0 | 0 | 0 | 0 | 0 | 0 | 0 | 0 | 1 | 0 | 0 | 0 | 1 |
| *Parvimonas micra* | 0 | 0 | 0 | 0 | 0 | 0 | 0 | 0 | 0 | 0 | 0 | 0 | 0 | 0 | 0 | 0 | 0 | 0 |
| *Petrimonas mucosa* | 0 | 0 | 0 | 0 | 6 | 1 | 0 | 0 | 0 | 0 | 0 | 0 | 0 | 0 | 0 | 0 | 0 | 0 |
| *Pirellula staleyi* | 0 | 0 | 0 | 0 | 0 | 0 | 0 | 0 | 0 | 0 | 0 | 0 | 0 | 0 | 0 | 0 | 0 | 0 |
| *Proteiniphilum saccharofermentans* | 0 | 0 | 1 | 1 | 13 | 0 | 0 | 0 | 0 | 0 | 0 | 0 | 0 | 0 | 0 | 0 | 0 | 0 |
| *Pseudoclostridium thermosuccinogenes* | 1 | 0 | 4 | 1 | 2 | 5 | 0 | 0 | 0 | 0 | 0 | 0 | 0 | 0 | 0 | 0 | 0 | 0 |
| *Pseudoduganella eburnea* | 1 | 0 | 3 | 4 | 3 | 0 | 0 | 0 | 0 | 0 | 0 | 0 | 0 | 1 | 0 | 0 | 0 | 1 |
| *Pseudoflavonifractor capillosus* | 0 | 0 | 0 | 1 | 4 | 0 | 0 | 0 | 0 | 0 | 0 | 0 | 0 | 0 | 0 | 0 | 0 | 1 |
| *Pseudomonas flexibilis* | 0 | 0 | 0 | 0 | 0 | 0 | 0 | 0 | 0 | 0 | 0 | 0 | 0 | 0 | 0 | 0 | 0 | 1 |
| *Pseudomonas lini* | 0 | 0 | 0 | 0 | 0 | 0 | 0 | 0 | 0 | 0 | 0 | 0 | 0 | 2 | 0 | 0 | 0 | 0 |
| *Reyranella soli* | 1 | 0 | 0 | 0 | 0 | 0 | 0 | 0 | 0 | 0 | 1 | 0 | 0 | 0 | 0 | 0 | 0 | 0 |
| *Rhodobacter* sp. LPB0142 | 2 | 0 | 0 | 0 | 0 | 0 | 0 | 0 | 0 | 0 | 0 | 0 | 0 | 0 | 0 | 0 | 0 | 1 |
| *Rhodopila globiformis* | 0 | 0 | 0 | 0 | 0 | 0 | 1 | 0 | 0 | 0 | 0 | 0 | 0 | 1 | 0 | 0 | 0 | 0 |
| *Rhodovulum sulfidophilum* | 0 | 0 | 0 | 0 | 0 | 0 | 1 | 0 | 0 | 0 | 0 | 0 | 0 | 0 | 4 | 0 | 0 | 1 |
| *Rickettsia prowazekii* | 0 | 0 | 0 | 0 | 0 | 0 | 0 | 0 | 0 | 0 | 0 | 0 | 0 | 0 | 0 | 0 | 0 | 0 |
| *Roseiarcus fermentans* | 2 | 0 | 0 | 0 | 0 | 0 | 1 | 0 | 0 | 0 | 0 | 0 | 0 | 1 | 0 | 0 | 0 | 0 |
| *Rudaea cellulosilytica* | 1 | 0 | 4 | 1 | 0 | 0 | 0 | 0 | 0 | 0 | 0 | 0 | 0 | 1 | 0 | 0 | 0 | 0 |
| *Rugosibacter aromaticivorans* | 0 | 0 | 0 | 0 | 0 | 0 | 0 | 0 | 0 | 0 | 0 | 0 | 0 | 1 | 0 | 0 | 1 | 0 |
| *Ruminiclostridium cellobioparum* | 0 | 0 | 2 | 2 | 6 | 3 | 1 | 0 | 0 | 0 | 0 | 0 | 0 | 0 | 0 | 0 | 0 | 0 |
| *Ruminococcus gnavus* | 3 | 0 | 1 | 2 | 7 | 0 | 0 | 0 | 0 | 0 | 0 | 0 | 0 | 0 | 0 | 0 | 0 | 0 |
| *Salmonella enterica* | 0 | 0 | 0 | 0 | 0 | 0 | 0 | 0 | 0 | 0 | 0 | 0 | 0 | 1 | 1 | 0 | 0 | 0 |
| *Sedimentibacter saalensis* | 0 | 0 | 0 | 0 | 0 | 0 | 0 | 0 | 0 | 0 | 0 | 0 | 0 | 0 | 2 | 0 | 0 | 1 |
| *Shewanella benthica* | 0 | 0 | 0 | 0 | 0 | 0 | 0 | 0 | 0 | 0 | 0 | 0 | 0 | 0 | 0 | 0 | 0 | 0 |
| *Singulisphaera acidiphila* | 0 | 0 | 0 | 0 | 0 | 0 | 0 | 0 | 0 | 0 | 0 | 0 | 0 | 0 | 0 | 0 | 0 | 0 |
| *Sinosporangium album* | 1 | 0 | 0 | 0 | 0 | 0 | 0 | 0 | 0 | 0 | 0 | 0 | 0 | 0 | 0 | 0 | 0 | 0 |
| *Solitalea koreensis* | 1 | 0 | 1 | 2 | 2 | 0 | 0 | 0 | 0 | 0 | 0 | 0 | 0 | 0 | 0 | 0 | 0 | 0 |
| *Sorangium cellulosum* | 1 | 0 | 0 | 0 | 0 | 0 | 3 | 0 | 1 | 2 | 1 | 0 | 0 | 0 | 0 | 0 | 0 | 0 |
| *Sphingorhabdus contaminans* | 0 | 0 | 1 | 1 | 0 | 0 | 0 | 0 | 0 | 0 | 0 | 0 | 0 | 0 | 0 | 0 | 0 | 0 |
| *Sporomusa malonica* | 0 | 0 | 0 | 0 | 0 | 0 | 0 | 0 | 0 | 0 | 0 | 0 | 0 | 0 | 1 | 0 | 0 | 1 |
| *Staphylococcus aureus* | 0 | 0 | 0 | 0 | 0 | 0 | 0 | 0 | 0 | 0 | 0 | 0 | 0 | 0 | 0 | 0 | 0 | 0 |
| *Stenotrophomonas maltophilia* | 1 | 0 | 0 | 0 | 0 | 0 | 0 | 0 | 0 | 0 | 0 | 0 | 0 | 1 | 0 | 0 | 0 | 0 |
| *Streptococcus pneumoniae* | 0 | 0 | 2 | 1 | 1 | 0 | 0 | 0 | 0 | 0 | 0 | 0 | 0 | 0 | 0 | 0 | 0 | 0 |
| *Streptococcus ruminantium* | 0 | 0 | 2 | 1 | 0 | 0 | 0 | 0 | 0 | 0 | 0 | 0 | 0 | 0 | 0 | 0 | 0 | 0 |
| *Streptococcus thermophilus* | 0 | 0 | 0 | 0 | 1 | 0 | 0 | 0 | 0 | 0 | 0 | 0 | 0 | 0 | 0 | 0 | 0 | 0 |
| *Streptomyces cinnamoneus* | 1 | 0 | 0 | 0 | 0 | 1 | 0 | 0 | 0 | 0 | 0 | 0 | 0 | 1 | 0 | 0 | 0 | 0 |
| *Streptomyces himastatinicus* | 3 | 0 | 3 | 5 | 7 | 1 | 0 | 0 | 1 | 2 | 0 | 0 | 0 | 3 | 0 | 0 | 0 | 0 |
| *Sutterella* sp. CAG:521 | 0 | 0 | 0 | 0 | 0 | 0 | 0 | 0 | 0 | 0 | 0 | 0 | 0 | 1 | 0 | 0 | 0 | 0 |
| *Synechococcus* sp. WH 8109 | 0 | 0 | 0 | 0 | 0 | 1 | 0 | 0 | 0 | 0 | 0 | 0 | 0 | 0 | 0 | 0 | 0 | 0 |
| *Syntrophobacter* sp. SbD1 | 0 | 0 | 0 | 0 | 0 | 0 | 0 | 0 | 0 | 0 | 0 | 0 | 0 | 0 | 0 | 0 | 0 | 0 |
| *Syntrophus aciditrophicus* | 0 | 0 | 0 | 0 | 0 | 0 | 0 | 0 | 0 | 0 | 0 | 0 | 0 | 0 | 0 | 0 | 0 | 0 |
| *Taylorella asinigenitalis* | 0 | 0 | 0 | 0 | 0 | 0 | 0 | 0 | 0 | 0 | 0 | 0 | 0 | 0 | 0 | 0 | 0 | 1 |
| *Telmatospirillum siberiense* | 1 | 0 | 0 | 0 | 0 | 0 | 0 | 0 | 0 | 0 | 0 | 0 | 0 | 3 | 0 | 0 | 0 | 0 |
| *Terrimicrobium sacchariphilum* | 1 | 0 | 5 | 4 | 6 | 6 | 0 | 0 | 0 | 0 | 0 | 0 | 0 | 1 | 0 | 0 | 0 | 0 |
| *Thermotalea metallivorans* | 1 | 0 | 0 | 0 | 0 | 0 | 0 | 0 | 0 | 0 | 0 | 0 | 0 | 0 | 0 | 0 | 0 | 0 |
| *Thermus thermophilus* | 2 | 0 | 1 | 0 | 0 | 0 | 0 | 0 | 0 | 0 | 0 | 0 | 0 | 0 | 0 | 0 | 0 | 0 |
| *Thiobacillus thioparus* | 0 | 0 | 0 | 0 | 0 | 0 | 0 | 0 | 0 | 0 | 0 | 0 | 0 | 0 | 0 | 0 | 0 | 0 |
| *Tissierella praeacuta* | 0 | 0 | 0 | 0 | 0 | 0 | 0 | 0 | 0 | 0 | 0 | 0 | 0 | 0 | 0 | 0 | 0 | 0 |
| *Vibrio parahaemolyticus* | 0 | 0 | 1 | 0 | 1 | 0 | 0 | 0 | 0 | 0 | 0 | 0 | 0 | 0 | 0 | 0 | 0 | 1 |
| *Virgibacillus profundi* | 1 | 0 | 0 | 1 | 1 | 2 | 0 | 0 | 0 | 0 | 0 | 0 | 0 | 0 | 0 | 0 | 0 | 0 |
| *Vulgatibacter incomptus* | 0 | 0 | 0 | 0 | 0 | 0 | 0 | 0 | 0 | 0 | 0 | 0 | 0 | 0 | 0 | 0 | 0 | 0 |
| *Xanthomonas citri* | 1 | 0 | 0 | 1 | 3 | 0 | 0 | 0 | 0 | 2 | 0 | 0 | 0 | 1 | 0 | 0 | 0 | 0 |
| *Yangia pacifica* | 1 | 0 | 0 | 0 | 0 | 0 | 0 | 0 | 0 | 0 | 0 | 0 | 0 | 0 | 1 | 0 | 0 | 3 |
| *Zavarzinella formosa* | 0 | 0 | 0 | 0 | 0 | 0 | 0 | 0 | 1 | 0 | 0 | 0 | 0 | 0 | 0 | 0 | 0 | 0 |

**Supplementary Table S11: The functional profiles of the enriched soil bacterial species related to phosphorus uptake and scavenging.** Only the enriched species with available genomes were displayed.

| **Species** | **PhoR/PhoB two-component system** | | | | **Phosphate specific transport (Pst) system** | | | | **C-P lyase pathway** | | | | | | | | **Phytase** | **Phosphatase** | | | |
| --- | --- | --- | --- | --- | --- | --- | --- | --- | --- | --- | --- | --- | --- | --- | --- | --- | --- | --- | --- | --- | --- |
|  | **phoU** | **phoR** | **phoB** | **phoE** | **pstS** | **pstC** | **pstB** | **pstA** | **phnP** | **phnM** | **phnL** | **phnJ** | **phnI** | **phnH** | **phnG** | **phnX** | **appA** | **phoD** | **phoAB** | **PHO** | **phoN** |
| *Acetobacter pomorum* | 1 | 0 | 1 | 0 | 2 | 1 | 1 | 1 | 1 | 0 | 0 | 0 | 0 | 0 | 0 | 0 | 0 | 0 | 0 | 0 | 1 |
| *Acetobacteroides hydrogenigenes* | 1 | 0 | 0 | 0 | 2 | 1 | 1 | 1 | 1 | 0 | 0 | 0 | 0 | 0 | 0 | 0 | 0 | 0 | 1 | 0 | 0 |
| *Acidisphaera rubrifaciens* | 1 | 1 | 1 | 0 | 3 | 1 | 1 | 1 | 1 | 0 | 0 | 0 | 0 | 0 | 0 | 0 | 0 | 0 | 0 | 0 | 0 |
| *Acinetobacter baumannii* | 1 | 1 | 1 | 0 | 1 | 1 | 1 | 1 | 0 | 0 | 0 | 0 | 0 | 0 | 0 | 1 | 0 | 1 | 0 | 0 | 0 |
| *Alkanindiges illinoisensis* | 1 | 1 | 1 | 0 | 1 | 1 | 1 | 1 | 0 | 0 | 0 | 0 | 0 | 0 | 0 | 0 | 0 | 0 | 0 | 0 | 0 |
| *Anaerocolumna aminovalerica* | 1 | 1 | 0 | 0 | 1 | 1 | 1 | 1 | 0 | 0 | 0 | 0 | 0 | 0 | 0 | 0 | 0 | 0 | 0 | 0 | 0 |
| *Anaerolinea thermophila* | 1 | 1 | 0 | 0 | 2 | 2 | 2 | 2 | 0 | 0 | 0 | 0 | 0 | 0 | 0 | 0 | 0 | 0 | 0 | 0 | 0 |
| *Anaeromyxobacter dehalogenans* | 1 | 0 | 0 | 0 | 1 | 1 | 1 | 1 | 0 | 0 | 0 | 0 | 0 | 0 | 0 | 0 | 0 | 0 | 2 | 0 | 0 |
| *Aquicella siphonis* | 0 | 0 | 0 | 0 | 0 | 0 | 0 | 0 | 0 | 0 | 0 | 0 | 0 | 0 | 0 | 1 | 0 | 0 | 0 | 0 | 0 |
| *Aquisphaera giovannonii* | 2 | 1 | 0 | 0 | 1 | 1 | 1 | 1 | 1 | 0 | 0 | 0 | 0 | 0 | 0 | 1 | 0 | 1 | 1 | 0 | 0 |
| *Azospirillum* sp. CAG:260 | 0 | 0 | 1 | 0 | 0 | 0 | 0 | 0 | 1 | 0 | 0 | 0 | 0 | 0 | 0 | 0 | 0 | 0 | 0 | 0 | 0 |
| *Bacillus cereus* | 1 | 3 | 0 | 0 | 2 | 2 | 1 | 2 | 0 | 0 | 0 | 0 | 0 | 0 | 0 | 1 | 0 | 0 | 2 | 0 | 0 |
| *Bacillus thuringiensis* | 1 | 3 | 0 | 0 | 2 | 2 | 1 | 2 | 0 | 0 | 0 | 0 | 0 | 0 | 0 | 1 | 0 | 0 | 2 | 0 | 0 |
| *Bradyrhizobium erythrophlei* | 1 | 1 | 1 | 0 | 1 | 1 | 1 | 1 | 1 | 0 | 0 | 0 | 0 | 0 | 0 | 0 | 0 | 1 | 0 | 0 | 0 |
| *Brevibacillus fluminis* | 1 | 3 | 0 | 0 | 3 | 2 | 3 | 2 | 0 | 0 | 0 | 0 | 0 | 0 | 0 | 2 | 0 | 0 | 1 | 0 | 0 |
| *Brucella suis* | 1 | 1 | 1 | 0 | 1 | 1 | 1 | 1 | 1 | 1 | 0 | 0 | 0 | 0 | 0 | 0 | 0 | 0 | 1 | 0 | 0 |
| *Burkholderia cenocepacia* | 1 | 1 | 1 | 0 | 1 | 1 | 1 | 1 | 0 | 0 | 0 | 0 | 0 | 0 | 0 | 0 | 0 | 0 | 2 | 0 | 0 |
| *Burkholderia cepacia* | 1 | 1 | 1 | 0 | 1 | 1 | 1 | 1 | 0 | 0 | 0 | 0 | 0 | 0 | 0 | 0 | 0 | 1 | 2 | 0 | 0 |
| *Burkholderia pseudomallei* | 1 | 1 | 1 | 0 | 1 | 1 | 1 | 1 | 0 | 1 | 1 | 1 | 1 | 1 | 1 | 0 | 0 | 0 | 2 | 0 | 0 |
| *Burkholderia pseudomultivorans* | 1 | 1 | 1 | 0 | 1 | 1 | 1 | 1 | 0 | 0 | 0 | 0 | 0 | 0 | 0 | 1 | 0 | 0 | 0 | 0 | 0 |
| *Bythopirellula goksoyri* | 1 | 1 | 0 | 0 | 2 | 1 | 1 | 1 | 1 | 0 | 0 | 0 | 0 | 0 | 0 | 0 | 0 | 1 | 2 | 0 | 0 |
| *Candidatus* Giovannonibacteria bacterium RIFCSPHIGHO2_12_FULL_44_42 | 0 | 0 | 0 | 0 | 0 | 0 | 0 | 0 | 0 | 0 | 0 | 0 | 0 | 0 | 0 | 0 | 0 | 0 | 0 | 0 | 0 |
| *Candidatus Koribacter versatilis* | 1 | 1 | 0 | 0 | 1 | 1 | 1 | 1 | 1 | 0 | 0 | 0 | 0 | 0 | 0 | 0 | 0 | 1 | 0 | 0 | 0 |
| *Candidatus* Levybacteria bacterium GW2011_GWB1_41_21 | 0 | 0 | 0 | 0 | 0 | 0 | 0 | 0 | 0 | 0 | 0 | 0 | 0 | 0 | 0 | 0 | 0 | 0 | 0 | 0 | 0 |
| *Candidatus* Liptonbacteria bacterium CG11_big_fil_rev_8_21_14_0_20_35_14 | 0 | 0 | 0 | 0 | 0 | 0 | 0 | 0 | 0 | 0 | 0 | 0 | 0 | 0 | 0 | 0 | 0 | 0 | 0 | 0 | 0 |
| *Candidatus* Rokubacteria bacterium | 1 | 2 | 1 | 0 | 2 | 1 | 1 | 1 | 0 | 0 | 0 | 0 | 0 | 0 | 0 | 0 | 0 | 1 | 0 | 0 | 0 |
| *Candidatus Solibacter usitatus* | 0 | 0 | 0 | 0 | 0 | 0 | 0 | 0 | 1 | 0 | 0 | 0 | 0 | 0 | 0 | 0 | 0 | 0 | 0 | 0 | 0 |
| *Candidatus Sulfopaludibacter* sp. SbA4 | 1 | 1 | 0 | 0 | 1 | 0 | 1 | 1 | 1 | 0 | 0 | 0 | 0 | 0 | 0 | 1 | 0 | 0 | 0 | 0 | 0 |
| *Candidatus Sulfotelmatobacter kueseliae* | 3 | 2 | 0 | 0 | 1 | 1 | 1 | 1 | 1 | 0 | 0 | 0 | 0 | 0 | 0 | 0 | 0 | 0 | 0 | 0 | 0 |
| *Candidatus* Woesebacteria bacterium RBG_16_36_11 | 0 | 0 | 0 | 0 | 0 | 0 | 0 | 0 | 0 | 0 | 0 | 0 | 0 | 0 | 0 | 0 | 0 | 0 | 0 | 0 | 0 |
| *Candidatus* Zambryskibacteria bacterium CG10_big_fil_rev_8_21_14_0_10_42_12 | 0 | 0 | 0 | 0 | 0 | 0 | 0 | 0 | 0 | 0 | 0 | 0 | 0 | 0 | 0 | 0 | 0 | 0 | 0 | 0 | 0 |
| *Carboxydocella* sp. JDF658 | 1 | 1 | 1 | 0 | 1 | 1 | 1 | 1 | 0 | 0 | 0 | 0 | 0 | 0 | 0 | 0 | 0 | 0 | 0 | 0 | 0 |
| *Chitinispirillum alkaliphilum* | 1 | 1 | 0 | 0 | 1 | 1 | 1 | 1 | 1 | 0 | 0 | 0 | 0 | 0 | 0 | 0 | 0 | 0 | 0 | 0 | 0 |
| *Chlamydia trachomatis* | 0 | 0 | 0 | 0 | 0 | 0 | 0 | 0 | 1 | 0 | 0 | 0 | 0 | 0 | 0 | 0 | 0 | 0 | 0 | 0 | 0 |
| *Chromobacterium* sp. ATCC 53434 | 1 | 1 | 2 | 0 | 3 | 1 | 1 | 1 | 1 | 1 | 1 | 1 | 1 | 1 | 1 | 0 | 0 | 1 | 2 | 0 | 0 |
| *Chthoniobacter flavus* | 1 | 1 | 0 | 0 | 2 | 2 | 2 | 1 | 1 | 0 | 0 | 0 | 0 | 0 | 0 | 0 | 0 | 1 | 1 | 0 | 0 |
| *Clostridioides difficile* | 1 | 1 | 0 | 0 | 1 | 1 | 1 | 1 | 1 | 1 | 1 | 1 | 1 | 1 | 1 | 1 | 0 | 0 | 0 | 0 | 0 |
| *Clostridium aurantibutyricum* | 1 | 1 | 0 | 0 | 1 | 1 | 1 | 1 | 0 | 0 | 0 | 0 | 0 | 0 | 0 | 0 | 0 | 0 | 0 | 0 | 0 |
| *Clostridium bolteae* | 1 | 0 | 0 | 0 | 2 | 2 | 2 | 1 | 1 | 0 | 0 | 0 | 0 | 0 | 0 | 1 | 0 | 0 | 0 | 0 | 0 |
| *Clostridium botulinum* | 1 | 1 | 0 | 0 | 2 | 2 | 2 | 2 | 0 | 0 | 0 | 0 | 0 | 0 | 0 | 0 | 0 | 0 | 0 | 0 | 0 |
| *Clostridium cadaveris* | 1 | 1 | 0 | 0 | 1 | 1 | 1 | 1 | 0 | 0 | 0 | 0 | 0 | 0 | 0 | 0 | 0 | 0 | 0 | 0 | 0 |
| *Clostridium cellulosi* | 0 | 0 | 0 | 0 | 0 | 0 | 0 | 0 | 0 | 0 | 0 | 0 | 0 | 0 | 0 | 0 | 0 | 0 | 0 | 0 | 0 |
| *Clostridium leptum* | 1 | 1 | 0 | 0 | 1 | 1 | 1 | 1 | 0 | 0 | 0 | 0 | 0 | 0 | 0 | 0 | 0 | 0 | 0 | 0 | 0 |
| *Clostridium* sp. BL8 | 1 | 1 | 1 | 0 | 1 | 1 | 2 | 1 | 2 | 0 | 0 | 0 | 0 | 0 | 0 | 0 | 0 | 0 | 0 | 0 | 0 |
| *Comamonas kerstersii* | 1 | 1 | 1 | 0 | 1 | 1 | 1 | 1 | 0 | 0 | 0 | 0 | 0 | 0 | 0 | 0 | 0 | 0 | 0 | 0 | 0 |
| *Comamonas testosteroni* | 1 | 1 | 1 | 0 | 1 | 1 | 1 | 1 | 0 | 0 | 0 | 0 | 0 | 0 | 0 | 1 | 0 | 1 | 0 | 0 | 0 |
| *Crenothrix polyspora* | 2 | 1 | 1 | 0 | 1 | 1 | 1 | 1 | 0 | 0 | 0 | 0 | 0 | 0 | 0 | 0 | 0 | 0 | 2 | 0 | 0 |
| *Cyanobium gracile* | 0 | 0 | 0 | 0 | 3 | 1 | 1 | 1 | 0 | 0 | 0 | 0 | 0 | 0 | 0 | 0 | 0 | 1 | 1 | 0 | 0 |
| *Delftia tsuruhatensis* | 1 | 1 | 1 | 0 | 1 | 1 | 1 | 1 | 0 | 0 | 0 | 0 | 0 | 0 | 0 | 1 | 0 | 2 | 2 | 0 | 0 |
| *Desulfomonile tiedjei* | 3 | 1 | 1 | 0 | 8 | 2 | 2 | 2 | 1 | 0 | 0 | 0 | 0 | 0 | 0 | 0 | 0 | 0 | 1 | 0 | 0 |
| *Desulfonauticus* sp. 38_4375 | 1 | 0 | 0 | 0 | 2 | 1 | 2 | 1 | 0 | 0 | 0 | 0 | 0 | 0 | 0 | 0 | 0 | 0 | 1 | 0 | 0 |
| *Desulfovibrio putealis* | 2 | 1 | 0 | 0 | 3 | 1 | 1 | 1 | 0 | 0 | 0 | 0 | 0 | 0 | 0 | 0 | 0 | 0 | 1 | 0 | 3 |
| *Dethiobacter alkaliphilus* | 1 | 2 | 0 | 0 | 0 | 0 | 0 | 0 | 0 | 0 | 0 | 0 | 0 | 0 | 0 | 0 | 0 | 0 | 0 | 0 | 0 |
| *Dictyobacter aurantiacus* | 1 | 0 | 0 | 0 | 2 | 1 | 2 | 0 | 0 | 0 | 0 | 0 | 0 | 0 | 0 | 0 | 0 | 2 | 0 | 0 | 0 |
| *Dinghuibacter silviterrae* | 1 | 0 | 0 | 0 | 4 | 2 | 2 | 1 | 1 | 2 | 1 | 1 | 1 | 1 | 1 | 1 | 0 | 0 | 0 | 0 | 0 |
| *Diplorickettsia massiliensis* | 0 | 0 | 0 | 0 | 0 | 0 | 0 | 0 | 0 | 0 | 0 | 0 | 0 | 0 | 0 | 0 | 0 | 0 | 0 | 0 | 0 |
| *Dorea longicatena* | 1 | 0 | 0 | 0 | 2 | 2 | 2 | 1 | 0 | 0 | 0 | 0 | 0 | 0 | 0 | 0 | 0 | 0 | 0 | 0 | 0 |
| *Duganella ginsengisoli* | 1 | 1 | 1 | 0 | 1 | 1 | 1 | 1 | 0 | 0 | 0 | 0 | 0 | 0 | 0 | 0 | 0 | 4 | 2 | 0 | 0 |
| *Dyella japonica* | 1 | 1 | 1 | 0 | 2 | 1 | 1 | 1 | 0 | 1 | 0 | 0 | 0 | 0 | 0 | 0 | 0 | 0 | 1 | 0 | 3 |
| *Dyella marensis* | 1 | 1 | 1 | 0 | 3 | 1 | 1 | 1 | 0 | 0 | 0 | 0 | 0 | 0 | 0 | 0 | 1 | 0 | 1 | 0 | 0 |
| *Elizabethkingia anophelis* | 0 | 0 | 0 | 0 | 1 | 0 | 0 | 0 | 1 | 0 | 0 | 0 | 0 | 0 | 0 | 1 | 0 | 0 | 2 | 0 | 0 |
| *Endomicrobium proavitum* | 2 | 0 | 0 | 0 | 2 | 2 | 3 | 2 | 0 | 0 | 0 | 0 | 0 | 0 | 0 | 0 | 0 | 0 | 0 | 0 | 0 |
| *Enterobacter cancerogenus* | 1 | 1 | 1 | 1 | 1 | 1 | 1 | 1 | 1 | 1 | 1 | 1 | 1 | 1 | 1 | 0 | 0 | 0 | 1 | 0 | 0 |
| *Enterococcus faecalis* | 1 | 2 | 0 | 0 | 2 | 1 | 2 | 1 | 1 | 0 | 0 | 0 | 0 | 0 | 0 | 0 | 0 | 0 | 1 | 0 | 0 |
| *Erythrobacter luteus* | 1 | 0 | 1 | 0 | 1 | 1 | 1 | 1 | 1 | 0 | 0 | 0 | 0 | 0 | 0 | 0 | 0 | 0 | 0 | 0 | 0 |
| *Escherichia coli* | 1 | 1 | 1 | 1 | 3 | 1 | 1 | 1 | 1 | 1 | 1 | 1 | 1 | 1 | 1 | 0 | 1 | 0 | 1 | 0 | 0 |
| *Fimbriimonas ginsengisoli* | 1 | 0 | 0 | 0 | 1 | 1 | 1 | 1 | 2 | 0 | 0 | 0 | 0 | 0 | 0 | 0 | 0 | 0 | 0 | 0 | 0 |
| *Flavisolibacter tropicus* | 0 | 0 | 0 | 0 | 1 | 0 | 0 | 0 | 1 | 0 | 0 | 0 | 0 | 0 | 0 | 0 | 0 | 0 | 0 | 0 | 0 |
| *Fonticella tunisiensis* | 2 | 1 | 0 | 0 | 2 | 1 | 1 | 1 | 0 | 0 | 0 | 0 | 0 | 0 | 0 | 0 | 0 | 0 | 1 | 0 | 0 |
| *Gaiella occulta* | 1 | 0 | 0 | 0 | 1 | 1 | 1 | 1 | 0 | 0 | 0 | 0 | 0 | 0 | 0 | 0 | 0 | 0 | 0 | 0 | 0 |
| *Gardnerella vaginalis* | 1 | 0 | 0 | 0 | 1 | 1 | 1 | 1 | 0 | 0 | 0 | 0 | 0 | 0 | 0 | 0 | 0 | 0 | 0 | 0 | 0 |
| *Geminisphaera colitermitum* | 1 | 1 | 0 | 0 | 3 | 1 | 1 | 1 | 1 | 0 | 0 | 0 | 0 | 0 | 0 | 0 | 0 | 0 | 1 | 0 | 0 |
| *Gemmata massiliana* | 0 | 1 | 0 | 0 | 1 | 1 | 1 | 1 | 1 | 0 | 0 | 0 | 0 | 0 | 0 | 1 | 0 | 2 | 0 | 0 | 0 |
| *Gemmatimonas phototrophica* | 0 | 1 | 0 | 0 | 2 | 1 | 2 | 1 | 1 | 0 | 0 | 0 | 0 | 0 | 0 | 0 | 0 | 2 | 0 | 0 | 0 |
| *Geobacter pelophilus* | 1 | 1 | 0 | 0 | 6 | 1 | 1 | 1 | 1 | 0 | 0 | 0 | 0 | 0 | 0 | 0 | 0 | 0 | 0 | 0 | 0 |
| *Haliangium ochraceum* | 1 | 1 | 0 | 0 | 2 | 1 | 1 | 1 | 0 | 0 | 0 | 0 | 0 | 0 | 0 | 0 | 0 | 4 | 0 | 0 | 0 |
| *Klebsiella oxytoca* | 1 | 1 | 1 | 1 | 1 | 1 | 1 | 1 | 2 | 1 | 1 | 1 | 1 | 1 | 1 | 1 | 0 | 0 | 1 | 0 | 1 |
| *Klebsiella pneumoniae* | 1 | 1 | 1 | 1 | 1 | 1 | 1 | 1 | 1 | 1 | 1 | 1 | 1 | 1 | 1 | 1 | 1 | 0 | 1 | 0 | 1 |
| *Ktedonobacter racemifer* | 2 | 0 | 1 | 0 | 3 | 1 | 3 | 1 | 0 | 2 | 1 | 1 | 1 | 1 | 1 | 0 | 0 | 1 | 0 | 0 | 0 |
| *Lactococcus garvieae* | 1 | 0 | 0 | 0 | 2 | 1 | 2 | 1 | 0 | 0 | 0 | 0 | 0 | 0 | 0 | 0 | 0 | 0 | 0 | 0 | 0 |
| *Lactococcus lactis* | 1 | 0 | 0 | 0 | 2 | 1 | 2 | 1 | 0 | 0 | 0 | 0 | 0 | 0 | 0 | 0 | 0 | 0 | 0 | 0 | 0 |
| *Legionella pneumophila* | 0 | 0 | 0 | 0 | 0 | 0 | 0 | 0 | 0 | 0 | 0 | 0 | 0 | 0 | 0 | 0 | 0 | 0 | 0 | 0 | 0 |
| *Lentimicrobium saccharophilum* | 0 | 0 | 0 | 0 | 1 | 1 | 1 | 0 | 1 | 0 | 0 | 0 | 0 | 0 | 0 | 0 | 0 | 0 | 2 | 0 | 0 |
| *Limisphaera ngatamarikiensis* | 2 | 1 | 0 | 0 | 2 | 2 | 3 | 2 | 1 | 0 | 0 | 0 | 0 | 0 | 0 | 0 | 0 | 0 | 0 | 0 | 0 |
| *Listeria monocytogenes* | 1 | 1 | 0 | 0 | 1 | 1 | 2 | 1 | 0 | 0 | 0 | 0 | 0 | 0 | 0 | 0 | 0 | 0 | 0 | 0 | 0 |
| *Listeria seeligeri* | 1 | 1 | 0 | 0 | 1 | 1 | 2 | 1 | 0 | 0 | 0 | 0 | 0 | 0 | 0 | 0 | 0 | 0 | 0 | 0 | 0 |
| *Litorilinea aerophila* | 2 | 1 | 0 | 0 | 2 | 1 | 1 | 1 | 1 | 0 | 0 | 0 | 0 | 0 | 0 | 0 | 0 | 0 | 1 | 0 | 0 |
| *Longilinea arvoryzae* | 1 | 1 | 0 | 0 | 2 | 2 | 2 | 2 | 0 | 0 | 0 | 0 | 0 | 0 | 0 | 0 | 0 | 0 | 0 | 0 | 0 |
| *Luteitalea pratensis* | 2 | 1 | 0 | 0 | 2 | 2 | 3 | 2 | 1 | 0 | 0 | 0 | 0 | 0 | 0 | 0 | 0 | 1 | 2 | 0 | 0 |
| *Magnetospirillum* sp. XM-1 | 2 | 1 | 1 | 0 | 3 | 2 | 3 | 1 | 1 | 0 | 0 | 0 | 0 | 0 | 0 | 0 | 0 | 0 | 0 | 0 | 0 |
| *Microcystis aeruginosa* | 1 | 0 | 0 | 0 | 6 | 3 | 4 | 3 | 0 | 0 | 0 | 0 | 0 | 0 | 0 | 0 | 0 | 0 | 0 | 0 | 0 |
| *Microvirga flocculans* | 1 | 1 | 1 | 0 | 1 | 1 | 1 | 1 | 1 | 1 | 1 | 1 | 1 | 1 | 1 | 0 | 0 | 0 | 0 | 0 | 0 |
| *Minicystis rosea* | 1 | 1 | 2 | 0 | 2 | 1 | 1 | 1 | 0 | 0 | 0 | 0 | 0 | 0 | 0 | 1 | 0 | 3 | 0 | 0 | 0 |
| *Moraxella osloensis* | 1 | 1 | 1 | 0 | 2 | 1 | 1 | 1 | 0 | 0 | 0 | 0 | 0 | 0 | 0 | 0 | 0 | 0 | 0 | 0 | 0 |
| *Morganella morganii* | 1 | 1 | 1 | 0 | 1 | 1 | 1 | 1 | 0 | 0 | 0 | 0 | 0 | 0 | 0 | 0 | 0 | 0 | 1 | 0 | 1 |
| *Mycobacteroides abscessus* | 2 | 0 | 0 | 0 | 1 | 1 | 1 | 1 | 0 | 0 | 0 | 0 | 0 | 0 | 0 | 0 | 0 | 1 | 1 | 0 | 0 |
| *Nitrospira japonica* | 1 | 1 | 0 | 0 | 3 | 1 | 1 | 1 | 0 | 0 | 0 | 0 | 0 | 0 | 0 | 0 | 0 | 2 | 0 | 0 | 0 |
| *Novimethylophilus kurashikiensis* | 1 | 1 | 1 | 0 | 1 | 1 | 1 | 1 | 1 | 0 | 0 | 0 | 0 | 0 | 0 | 0 | 0 | 0 | 0 | 0 | 0 |
| *Novosphingobium rosa* | 2 | 0 | 1 | 0 | 2 | 2 | 2 | 1 | 1 | 0 | 0 | 0 | 0 | 0 | 0 | 1 | 2 | 2 | 0 | 0 | 3 |
| *Opitutus terrae* | 1 | 1 | 0 | 0 | 2 | 1 | 1 | 1 | 1 | 0 | 0 | 0 | 0 | 0 | 0 | 0 | 0 | 0 | 0 | 0 | 0 |
| *Oscillochloris trichoides* | 1 | 0 | 0 | 0 | 2 | 1 | 1 | 1 | 1 | 1 | 1 | 1 | 1 | 1 | 1 | 0 | 0 | 0 | 0 | 0 | 0 |
| *Paenarthrobacter nicotinovorans* | 1 | 0 | 0 | 0 | 1 | 1 | 1 | 1 | 0 | 0 | 0 | 0 | 0 | 0 | 0 | 0 | 0 | 2 | 0 | 0 | 0 |
| *Paenibacillus herberti* | 1 | 3 | 0 | 0 | 1 | 1 | 2 | 1 | 1 | 0 | 0 | 0 | 0 | 0 | 0 | 0 | 0 | 0 | 1 | 0 | 0 |
| *Paeniclostridium sordellii* | 1 | 1 | 0 | 0 | 2 | 1 | 1 | 1 | 1 | 0 | 0 | 0 | 0 | 0 | 0 | 0 | 0 | 0 | 0 | 0 | 0 |
| *Paludisphaera borealis* | 2 | 1 | 0 | 0 | 1 | 1 | 1 | 1 | 1 | 0 | 0 | 0 | 0 | 0 | 0 | 0 | 0 | 1 | 1 | 0 | 0 |
| *Pantoea ananatis* | 1 | 1 | 1 | 1 | 2 | 2 | 2 | 2 | 1 | 1 | 1 | 1 | 1 | 1 | 1 | 0 | 1 | 0 | 1 | 0 | 0 |
| *Paraburkholderia nodosa* | 2 | 1 | 1 | 0 | 5 | 1 | 1 | 1 | 0 | 1 | 1 | 1 | 1 | 1 | 1 | 1 | 0 | 0 | 0 | 0 | 0 |
| *Paraburkholderia susongensis* | 1 | 1 | 1 | 0 | 1 | 1 | 1 | 1 | 0 | 1 | 1 | 1 | 0 | 1 | 1 | 1 | 0 | 0 | 0 | 0 | 0 |
| *Paracoccus denitrificans* | 1 | 1 | 1 | 0 | 1 | 1 | 1 | 1 | 1 | 2 | 1 | 1 | 1 | 1 | 1 | 0 | 0 | 0 | 0 | 0 | 0 |
| *Paracoccus yeei* | 1 | 1 | 1 | 0 | 1 | 1 | 1 | 1 | 1 | 2 | 0 | 0 | 0 | 0 | 0 | 0 | 0 | 0 | 1 | 0 | 0 |
| *Parafilimonas terrae* | 0 | 0 | 0 | 0 | 2 | 1 | 1 | 0 | 1 | 0 | 0 | 0 | 0 | 0 | 0 | 0 | 0 | 0 | 0 | 0 | 0 |
| *Pararhodospirillum photometricum* | 2 | 1 | 1 | 0 | 1 | 1 | 1 | 1 | 1 | 0 | 0 | 0 | 0 | 0 | 0 | 1 | 0 | 0 | 0 | 0 | 0 |
| *Parvimonas micra* | 0 | 0 | 0 | 0 | 1 | 1 | 0 | 0 | 0 | 0 | 0 | 0 | 0 | 0 | 0 | 0 | 0 | 0 | 0 | 0 | 0 |
| *Petrimonas mucosa* | 0 | 0 | 0 | 0 | 0 | 0 | 0 | 0 | 1 | 0 | 0 | 0 | 0 | 0 | 0 | 0 | 0 | 0 | 5 | 0 | 0 |
| *Pirellula staleyi* | 1 | 1 | 0 | 0 | 1 | 1 | 2 | 1 | 1 | 0 | 0 | 0 | 0 | 0 | 0 | 1 | 0 | 4 | 0 | 0 | 0 |
| *Proteiniphilum saccharofermentans* | 1 | 0 | 0 | 0 | 2 | 1 | 1 | 1 | 1 | 0 | 0 | 0 | 0 | 0 | 0 | 0 | 0 | 0 | 2 | 0 | 0 |
| *Pseudoclostridium thermosuccinogenes* | 1 | 2 | 0 | 0 | 1 | 1 | 1 | 1 | 2 | 0 | 0 | 0 | 0 | 0 | 0 | 0 | 0 | 0 | 0 | 0 | 0 |
| *Pseudoduganella eburnea* | 1 | 1 | 1 | 0 | 2 | 1 | 1 | 1 | 0 | 0 | 0 | 0 | 0 | 0 | 0 | 0 | 0 | 2 | 2 | 0 | 0 |
| *Pseudoflavonifractor capillosus* | 1 | 2 | 0 | 0 | 1 | 1 | 1 | 1 | 0 | 0 | 0 | 0 | 0 | 0 | 0 | 0 | 0 | 0 | 1 | 0 | 0 |
| *Pseudomonas flexibilis* | 1 | 1 | 1 | 0 | 2 | 1 | 1 | 1 | 0 | 0 | 0 | 0 | 0 | 0 | 0 | 0 | 0 | 1 | 0 | 0 | 0 |
| *Pseudomonas lini* | 1 | 1 | 1 | 0 | 2 | 1 | 1 | 1 | 0 | 0 | 0 | 0 | 0 | 0 | 0 | 1 | 0 | 3 | 0 | 0 | 0 |
| *Reyranella soli* | 1 | 1 | 1 | 0 | 2 | 1 | 2 | 1 | 1 | 1 | 1 | 1 | 1 | 1 | 1 | 0 | 1 | 2 | 1 | 0 | 1 |
| *Rhodobacter* sp. LPB0142 | 1 | 1 | 1 | 0 | 1 | 1 | 1 | 1 | 1 | 0 | 0 | 0 | 0 | 0 | 0 | 0 | 0 | 0 | 0 | 0 | 0 |
| *Rhodopila globiformis* | 1 | 1 | 1 | 0 | 1 | 1 | 1 | 1 | 1 | 2 | 1 | 1 | 1 | 1 | 1 | 0 | 0 | 0 | 0 | 0 | 0 |
| *Rhodovulum sulfidophilum* | 1 | 1 | 1 | 0 | 2 | 1 | 1 | 1 | 1 | 2 | 0 | 0 | 0 | 0 | 0 | 0 | 0 | 0 | 2 | 0 | 0 |
| *Rickettsia prowazekii* | 0 | 0 | 0 | 0 | 0 | 0 | 0 | 0 | 1 | 0 | 0 | 0 | 0 | 0 | 0 | 0 | 0 | 0 | 0 | 0 | 0 |
| *Roseiarcus fermentans* | 2 | 0 | 1 | 0 | 3 | 1 | 2 | 1 | 1 | 0 | 0 | 0 | 0 | 0 | 0 | 1 | 0 | 0 | 0 | 0 | 0 |
| *Rudaea cellulosilytica* | 1 | 1 | 1 | 0 | 7 | 1 | 1 | 1 | 0 | 0 | 0 | 0 | 0 | 0 | 0 | 0 | 0 | 1 | 1 | 0 | 1 |
| *Rugosibacter aromaticivorans* | 1 | 1 | 1 | 0 | 1 | 1 | 1 | 0 | 0 | 0 | 0 | 0 | 0 | 0 | 0 | 0 | 0 | 0 | 0 | 0 | 0 |
| *Ruminiclostridium cellobioparum* | 1 | 2 | 0 | 0 | 2 | 2 | 1 | 2 | 1 | 0 | 0 | 0 | 0 | 0 | 0 | 0 | 0 | 0 | 1 | 0 | 0 |
| *Ruminococcus gnavus* | 1 | 0 | 0 | 0 | 1 | 1 | 1 | 1 | 0 | 0 | 0 | 0 | 0 | 0 | 0 | 1 | 0 | 0 | 0 | 0 | 0 |
| *Salmonella enterica* | 1 | 1 | 1 | 1 | 2 | 1 | 1 | 1 | 0 | 0 | 0 | 0 | 0 | 0 | 0 | 1 | 0 | 0 | 0 | 0 | 1 |
| *Sedimentibacter saalensis* | 1 | 2 | 0 | 0 | 3 | 2 | 2 | 1 | 0 | 0 | 0 | 0 | 0 | 0 | 0 | 0 | 0 | 0 | 1 | 0 | 0 |
| *Shewanella benthica* | 1 | 0 | 1 | 0 | 1 | 1 | 1 | 1 | 0 | 0 | 0 | 0 | 0 | 0 | 0 | 0 | 0 | 0 | 2 | 0 | 0 |
| *Singulisphaera acidiphila* | 1 | 1 | 0 | 0 | 1 | 1 | 1 | 1 | 1 | 0 | 0 | 0 | 0 | 0 | 0 | 1 | 0 | 2 | 1 | 0 | 0 |
| *Sinosporangium album* | 1 | 0 | 0 | 0 | 1 | 1 | 1 | 1 | 0 | 0 | 0 | 0 | 0 | 0 | 0 | 0 | 0 | 3 | 0 | 0 | 0 |
| *Solitalea koreensis* | 1 | 0 | 0 | 0 | 2 | 1 | 1 | 1 | 1 | 0 | 0 | 0 | 0 | 0 | 0 | 0 | 0 | 0 | 0 | 0 | 0 |
| *Sorangium cellulosum* | 1 | 2 | 1 | 0 | 1 | 1 | 1 | 1 | 0 | 0 | 0 | 0 | 0 | 0 | 0 | 0 | 0 | 4 | 0 | 0 | 0 |
| *Sphingorhabdus contaminans* | 1 | 0 | 1 | 0 | 1 | 1 | 1 | 1 | 0 | 0 | 0 | 0 | 0 | 0 | 0 | 0 | 0 | 2 | 1 | 0 | 0 |
| *Sporomusa malonica* | 2 | 1 | 0 | 0 | 2 | 1 | 1 | 1 | 1 | 0 | 0 | 0 | 0 | 0 | 0 | 1 | 0 | 0 | 3 | 0 | 0 |
| *Staphylococcus aureus* | 1 | 1 | 0 | 0 | 1 | 1 | 1 | 1 | 0 | 0 | 0 | 0 | 0 | 0 | 0 | 0 | 0 | 0 | 1 | 0 | 0 |
| *Stenotrophomonas maltophilia* | 1 | 0 | 1 | 0 | 3 | 1 | 1 | 1 | 0 | 0 | 0 | 0 | 0 | 0 | 0 | 0 | 0 | 2 | 2 | 0 | 1 |
| *Streptococcus pneumoniae* | 2 | 0 | 0 | 0 | 2 | 2 | 3 | 2 | 0 | 0 | 0 | 0 | 0 | 0 | 0 | 0 | 0 | 0 | 0 | 0 | 0 |
| *Streptococcus ruminantium* | 1 | 0 | 0 | 0 | 1 | 1 | 2 | 1 | 0 | 0 | 0 | 0 | 0 | 0 | 0 | 0 | 0 | 0 | 0 | 0 | 0 |
| *Streptococcus thermophilus* | 1 | 0 | 0 | 0 | 1 | 1 | 2 | 1 | 0 | 0 | 0 | 0 | 0 | 0 | 0 | 0 | 0 | 0 | 0 | 0 | 0 |
| *Streptomyces cinnamoneus* | 1 | 0 | 0 | 0 | 1 | 1 | 1 | 0 | 0 | 0 | 0 | 0 | 0 | 0 | 0 | 0 | 0 | 4 | 2 | 0 | 0 |
| *Streptomyces himastatinicus* | 1 | 0 | 0 | 0 | 2 | 1 | 1 | 1 | 0 | 0 | 0 | 0 | 0 | 0 | 0 | 0 | 0 | 1 | 0 | 0 | 0 |
| *Sutterella* sp. CAG:521 | 1 | 1 | 0 | 0 | 1 | 1 | 1 | 0 | 0 | 0 | 0 | 0 | 0 | 0 | 0 | 1 | 0 | 0 | 1 | 0 | 0 |
| *Synechococcus* sp. WH 8109 | 0 | 0 | 0 | 0 | 2 | 1 | 1 | 1 | 0 | 0 | 0 | 0 | 0 | 0 | 0 | 0 | 0 | 0 | 0 | 0 | 0 |
| *Syntrophobacter* sp. SbD1 | 2 | 0 | 0 | 0 | 4 | 1 | 1 | 2 | 0 | 0 | 0 | 0 | 0 | 0 | 0 | 0 | 0 | 0 | 0 | 0 | 0 |
| *Syntrophus aciditrophicus* | 1 | 1 | 1 | 0 | 2 | 1 | 2 | 1 | 0 | 0 | 0 | 0 | 0 | 0 | 0 | 0 | 0 | 0 | 0 | 0 | 0 |
| *Taylorella asinigenitalis* | 1 | 0 | 1 | 0 | 1 | 1 | 1 | 1 | 0 | 0 | 0 | 0 | 0 | 0 | 0 | 0 | 0 | 0 | 1 | 0 | 0 |
| *Telmatospirillum siberiense* | 2 | 1 | 1 | 0 | 5 | 1 | 2 | 1 | 1 | 2 | 1 | 1 | 1 | 1 | 1 | 0 | 1 | 0 | 0 | 0 | 4 |
| *Terrimicrobium sacchariphilum* | 1 | 1 | 0 | 0 | 1 | 1 | 1 | 1 | 1 | 0 | 0 | 0 | 0 | 0 | 0 | 0 | 0 | 0 | 1 | 0 | 1 |
| *Thermotalea metallivorans* | 2 | 2 | 0 | 0 | 2 | 2 | 2 | 2 | 0 | 0 | 0 | 0 | 0 | 0 | 0 | 0 | 0 | 0 | 0 | 0 | 0 |
| *Thermus thermophilus* | 1 | 0 | 0 | 0 | 1 | 1 | 1 | 1 | 0 | 0 | 0 | 0 | 0 | 0 | 0 | 0 | 0 | 0 | 1 | 0 | 0 |
| *Thiobacillus thioparus* | 1 | 1 | 1 | 0 | 1 | 1 | 1 | 1 | 0 | 0 | 0 | 0 | 0 | 0 | 0 | 0 | 0 | 0 | 0 | 0 | 0 |
| *Tissierella praeacuta* | 1 | 1 | 0 | 0 | 2 | 2 | 2 | 1 | 0 | 0 | 0 | 0 | 0 | 0 | 0 | 0 | 0 | 0 | 1 | 0 | 0 |
| *Vibrio parahaemolyticus* | 1 | 1 | 1 | 0 | 3 | 2 | 2 | 2 | 0 | 0 | 0 | 0 | 0 | 0 | 0 | 1 | 0 | 2 | 2 | 0 | 0 |
| *Virgibacillus profundi* | 1 | 1 | 0 | 0 | 1 | 1 | 1 | 1 | 1 | 0 | 0 | 0 | 0 | 0 | 0 | 0 | 0 | 0 | 1 | 0 | 0 |
| *Vulgatibacter incomptus* | 1 | 1 | 0 | 0 | 1 | 1 | 1 | 1 | 0 | 0 | 0 | 0 | 0 | 0 | 0 | 0 | 0 | 1 | 2 | 0 | 0 |
| *Xanthomonas citri* | 1 | 0 | 1 | 0 | 2 | 1 | 1 | 1 | 0 | 0 | 0 | 0 | 0 | 0 | 0 | 0 | 2 | 2 | 1 | 0 | 1 |
| *Yangia pacifica* | 1 | 1 | 1 | 0 | 2 | 1 | 1 | 1 | 1 | 3 | 1 | 1 | 1 | 1 | 1 | 0 | 0 | 0 | 1 | 0 | 0 |
| *Zavarzinella formosa* | 1 | 1 | 0 | 0 | 2 | 1 | 1 | 1 | 1 | 0 | 0 | 0 | 0 | 0 | 0 | 1 | 0 | 3 | 0 | 0 | 0 |

**Supplementary Table S12**: The functional genes related to nitrogen, sulphur, carbon, and phosphorus metabolisms and their KEGG ortholog numbers

| **Metabolic process** | **Gene symbol** | **Key enzyme** | **KEGG ortholog(s)** |
| --- | --- | --- | --- |
| **A. *Nitrogen biogeochemical pathway*** | | | |
| Nitrogen fixation | nifDKH | molybdenum-mediated nitrogenase | K02586 (nifD), K02591 (nifK), K02588 (nifH), K00531 (anfG) |
|  | vnfDKGH | vanadium-mediated nitrogenase | K22896 (vnfD), K22897 (vnfK), K22898 (vnfG), K22899 (vnfH) |
| Assimilatory nitrate reduction | narB | ferredoxin-nitrate reductase | K00367 (narB) |
|  | NR | NAD(P)H-nitrate reductase | K10534 (NR) |
|  | nasAB | assimilatory nitrate reductase | K00372 (nasA), K00360 (nasB) |
| Assimilatory nitrite reduction | nit-6 | NAD(P)H-nitrite reductase | K17877 (nit-6) |
|  | nirA | ferredoxin-nitrite reductase | K00366 (nirA) |
| Dissimilatory nitrate reduction | narGHI | nitrate reductase / nitrite oxidoreductase | K00370 (narG), K00371 (narH), K00374 (narI) |
|  | napAB | cytochrome-nitrate reductase | K02567 (napA), K02568 (napB) |
| Dissimilatory nitrite reduction | nirBD | NADH-nitrite reductase | K00362 (nirB), K00363 (nirD) |
|  | nrfAH | cytochrome c-552 nitrite reductase | K03385 (nrfA), K15876 (nrfH) |
| Denitrification  NO_2_^-^ → NO | nirS | nitrite reductase (NO-forming) | K15864 (nirS) |
|  | nirK | nitrite reductase (NO-forming) | K00368 (nirK) |
| Denitrification  NO → N_2_O | norBC | nitric oxide reductase | K04561 (norB), K02305 (norC) |
| Denitrification  N_2_O → N_2_ | nosZ | nitrous-oxide reductase | K00376 (nosZ) |
| Nitrification  NH_4_^+^ → NH_2_OH | pmo-amo | methane/ammonia monooxygenase | K10944 (pmoA-amoA), K10945 (pmoB-amoB), K10946 (pmoC-amoC) |
| Nitrification  NH_2_OH -> NO_2_^-^ | hao | hydroxylamine dehydrogenase | K10535 (hao) |
| **B. *Sulphur biogeochemical pathway*** | | | |
| Assimilatory sulfate reduction SO4 → APS | sat | sulfate adenylyltransferase | K00958 (sat) |
|  | cysNCD | bifunctional enzyme CysN/CysC | K00955 (cysNC), K00956 (cysN), K00957 (cysD) |
| Assimilatory sulfate reduction APS → PAPS | cysC | adenylylsulfate kinase | K00860 (cysC) |
|  | PAPSS | 3'-phosphoadenosine 5'-phosphosulfate synthase | K13811 (PAPSS) |
| Assimilatory sulfate reduction PAPS → Sulfite | cysH | phosphoadenosine phosphosulfate reductase | K00390 (cysH) |
| Assimilatory sulfate reduction  Sulfite → Sulfide | cysJI | NADPH-sulfite reductase | K00380 (cysJ), K00381 (cysI) |
|  | sir | ferredoxin-sulfite reductase | K00392 (sir) |
| Dissimilatory sulfate reduction and oxidation  H_2_S → S2O3 | dsrAB | dissimilatory sulfite reductase | K11180 (dsrA), K11181 (dsrB) |
| Dissimilatory sulfate reduction and oxidation  S_2_O_3_ → APS | aprAB | adenylylsulfate reductase | K00394 (aprA), K00395 (aprB) |
| SOX system Sulfide/Thiosulfate → Sox Y-S-* | soxAX | L-cysteine S-thiosulfotransferase | K17222 (soxA), K17223 (soxX) |
| SOX system SoxYZ-S-* → Sulfate | soxB | S-sulfosulfanyl-L-cysteine sulfohydrolase | K17224 (soxB) |
| SOX system SoxYZ-S-SH ←→ SoxYZ-S-SO_3_ | soxCD | sulfane dehydrogenase | K17225 (soxC), K22622 (soxD) |
| Sulfite detoxification | soeABC | quinone-dependent sulfite dehydrogenase | K21307 (soeA), K21308 (soeB), K21309 (soeC) |
|  | suox | sulfite oxidase | K00387 (SUOX) |
| Sulfur oxidation to sulfide | fccAB | sulfide dehydrogenase | K17229 (fccB), K17230 (fccA) |
|  | sqr | Sulfide-quinone oxidoreductase | K17218 (sqr) |
| Anaerobic sulfate reduction | asrABC | anaerobic sulfite reductase | K16950 (asrA), K16951 (asrB), K00385 (asrC) |
|  | fsr | coenzyme F420-dependent sulfite reductase | K21816 (fsr) |
| **C. *Carbohydrates and aromatic compound utilisation*** | | | |
| Carbohydrate utilisation (Alpha-glucosidases) | malZ | alpha-glucosidase | K01187 (malZ) |
| Carbohydrate utilisation (Beta-glucosidases) | glvA | maltose-6'-phosphate glucosidase | K01232 (glvA) |
| Carbohydrate utilisation (Alpha-galactosidase) | galA | alpha-galactosidase | K07407 (galA) |
| Carbohydrate utilisation (Beta-galactosidase) | lacA | Beta-galactosidase | K12308 (lacA) |
|  | lacZ | Beta-galactosidase | K01190 (lacZ) |
| Carbohydrate utilisation (Alpha-mannosidase) | MAN2C1 | alpha-mannosidase | K01191 (MAN2C1) |
| Carbohydrate utilisation (Glucoamylase) | sga1 | glucoamylase | K01178 (sga1) |
| Carbohydrate utilisation (Cellobiohydrolase) | cbh1 | cellulose 1,4-beta-cellobiosidase | K01225 (cbh1) |
|  | cbhA | cellulose 1,4-beta-cellobiosidase | K19668 (cbhA) |
| Carbohydrate utilisation (Pectate lyase) | pel | pectate lyase | K01728 (pel) |
| Aromatic compound utilisation (Aromatic ring hydroxylase) | ethA | FAD-containing monooxygenase | K10215 (ethA) |
|  | nagH | salicylate 5-hydroxylase | K18243 (nagH) |
|  | nagG | salicylate 5-hydroxylase | K18242 (nagG) |
| Aromatic compound utilisation (TRAP C4 dicarboxylate transporter) | dctA | aerobic C4-dicarboxylate transport protein | K11103 (dctA) |
|  | dctP | TRAP-type transport system periplasmic protein | K21395 (dctP) |
| Aromatic compound utilisation (Extradiol dioxygenase) | phdF | extradiol dioxygenase | K11945 (phdF) |
| Aromatic compound utilisation (Hydroxychromene-carboxylate) | nahD | 2-hydroxychromene-2-carboxylate isomerase | K14584 (nahD) |
| Aromatic compound utilisation (TRAP mannitol/chloroaromatic) | dctM | C4-dicarboxylate transporter | K11690 (dctM) |
| **D. *Phosphorus uptake and scavenging*** | | | |
| PhoR/PhoB two-component system | phoU | PhoR/PhoB Inhibitor Protein | K02039 (phoU) |
|  | phoR | Phosphate Regulon Sensor Histidine Kinase | K07636 (phoR) |
|  | phoB | Phosphate Regulon Response Regulator | K07657 (phoB) |
|  | phoE | Outer Membrane Pore Protein E | K11929 (phoE) |
| Phosphate specific transport (Pst) system | pstS | Phosphate-Specific Transport System Subunit | K02040 (pstS) |
|  | pstC | Phosphate-Specific Transport System Subunit | K02037 (pstC) |
|  | pstB | Phosphate-Specific Transport System Subunit | K02036 (pstB) |
|  | pstA | Phosphate-Specific Transport System Subunit | K02038 (pstA) |
| C-P lyase pathway | phnP | C-P Lyase Subunit | K06167 (phnP) |
|  | phnM | C-P Lyase Subunit | K06162 (phnM) |
|  | phnL | C-P Lyase Subunit | K05780 (phnL) |
|  | phnJ | C-P Lyase Subunit | K06163 (phnJ) |
|  | phnI | C-P Lyase Subunit | K06164 (phnI) |
|  | phnH | C-P Lyase Subunit | K06165 (phnH) |
|  | phnG | C-P Lyase Subunit | K06166 (phnG) |
|  | phnX | Phosphonatase | K05306 (phnX) |
| Phytase | appA | Phytase | K01093 (appA) |
| Phosphatase | phoD | Alkaline phosphatase | K01113 (phoD) |
|  | phoAB | Alkaline phosphatase | K01077 (phoAB) |
|  | PHO | Acid phosphatase | K01078 (PHO) |
|  | phoN | Acid phosphatase | K09474 (phoN) |
